# Supplementary figures and images for: GI-16 lineage (624/I or Q1), there and back again: The history of one of the major threats for poultry farming of our era
Source: PLoS One. 2018 Dec 20;13(12):e0203513. doi: 10.1371/journal.pone.0203513 (PMC6301571; doi:10.1371/journal.pone.0203513)

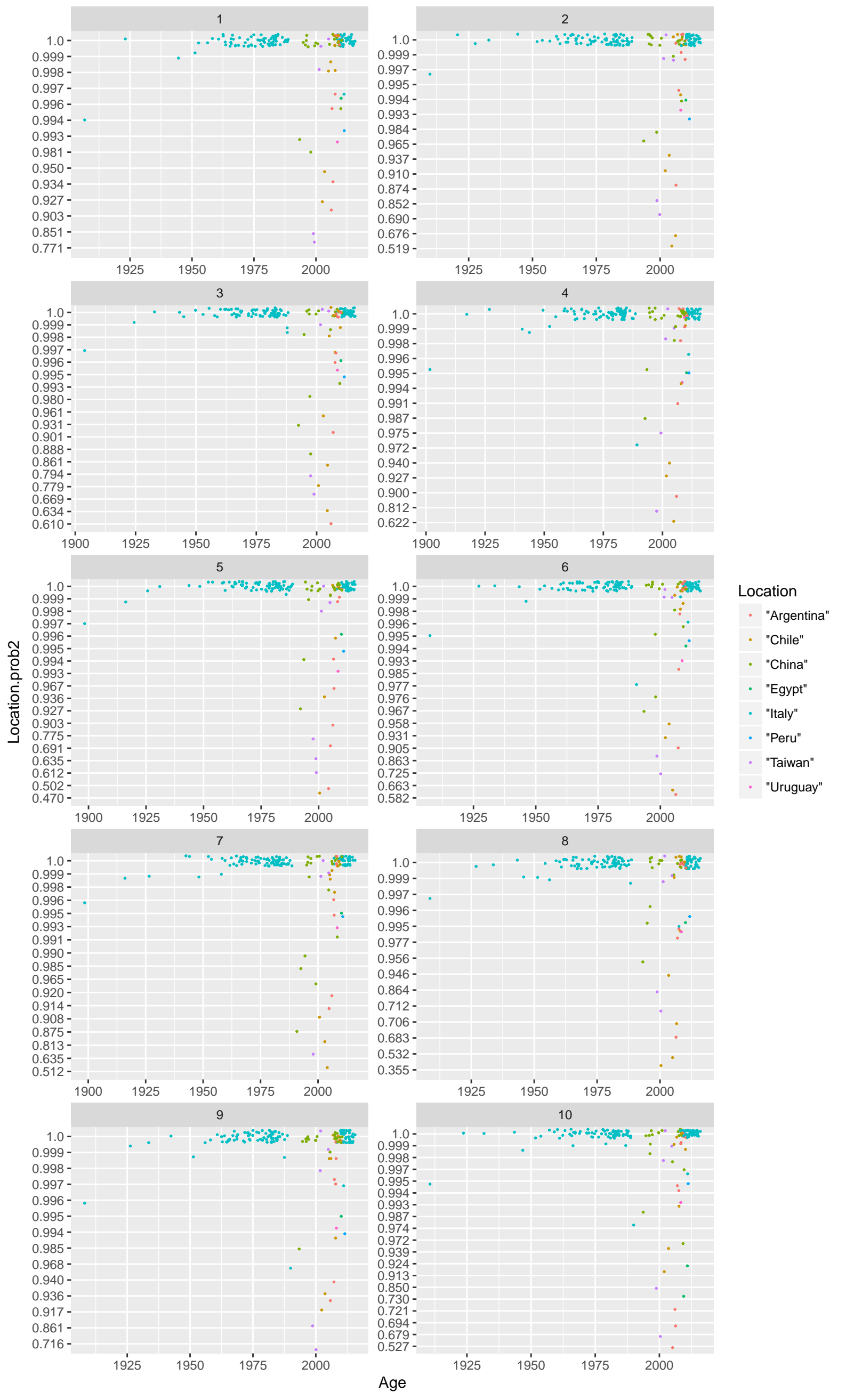

Supplement: S1 Fig — Scatterplot representing the posterior probability of each ancestral location (color-coded) prediction over time. The results of ten independent BEAST run are reported. (PDF) [file pone.0203513.s001.pdf]

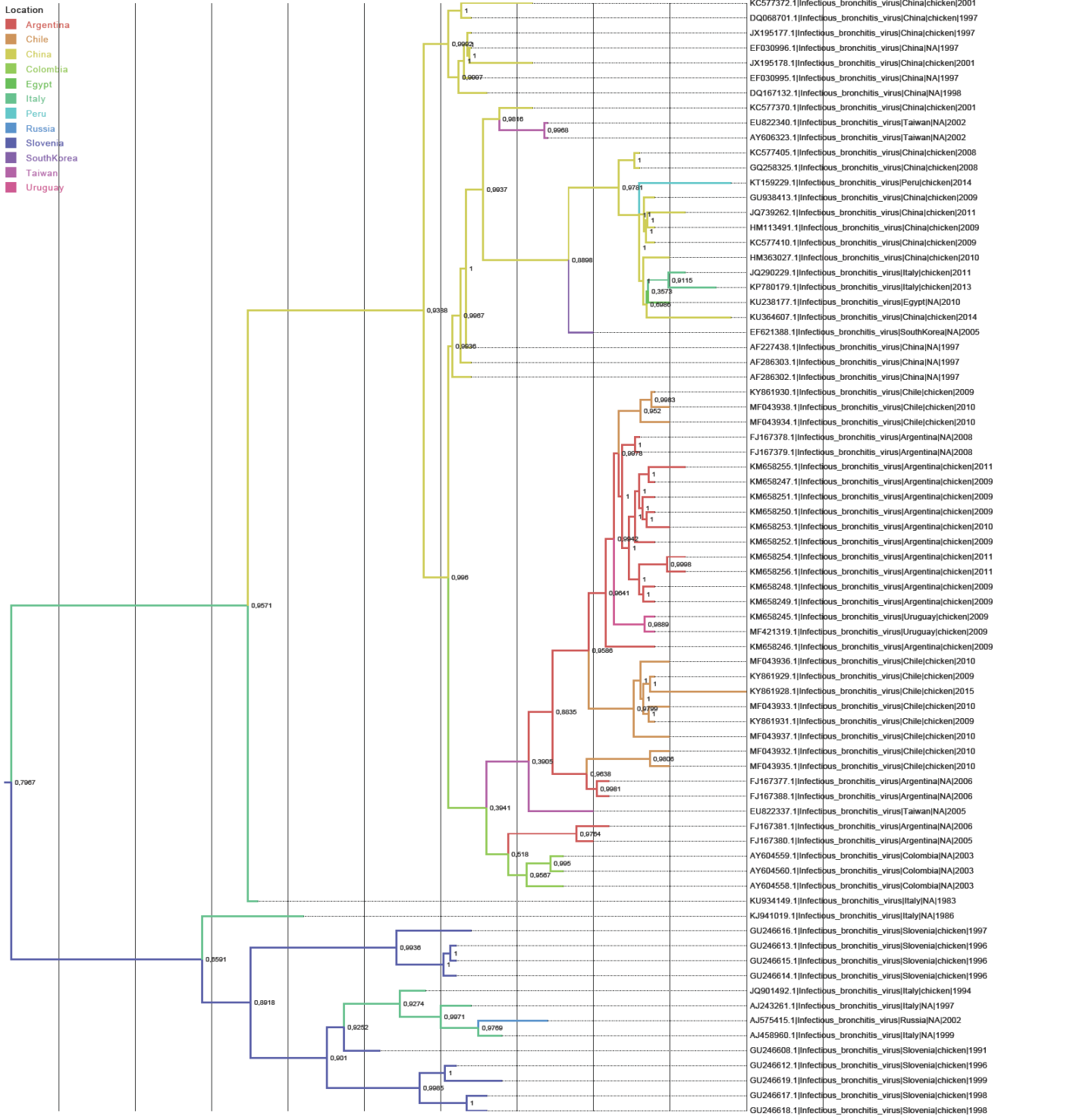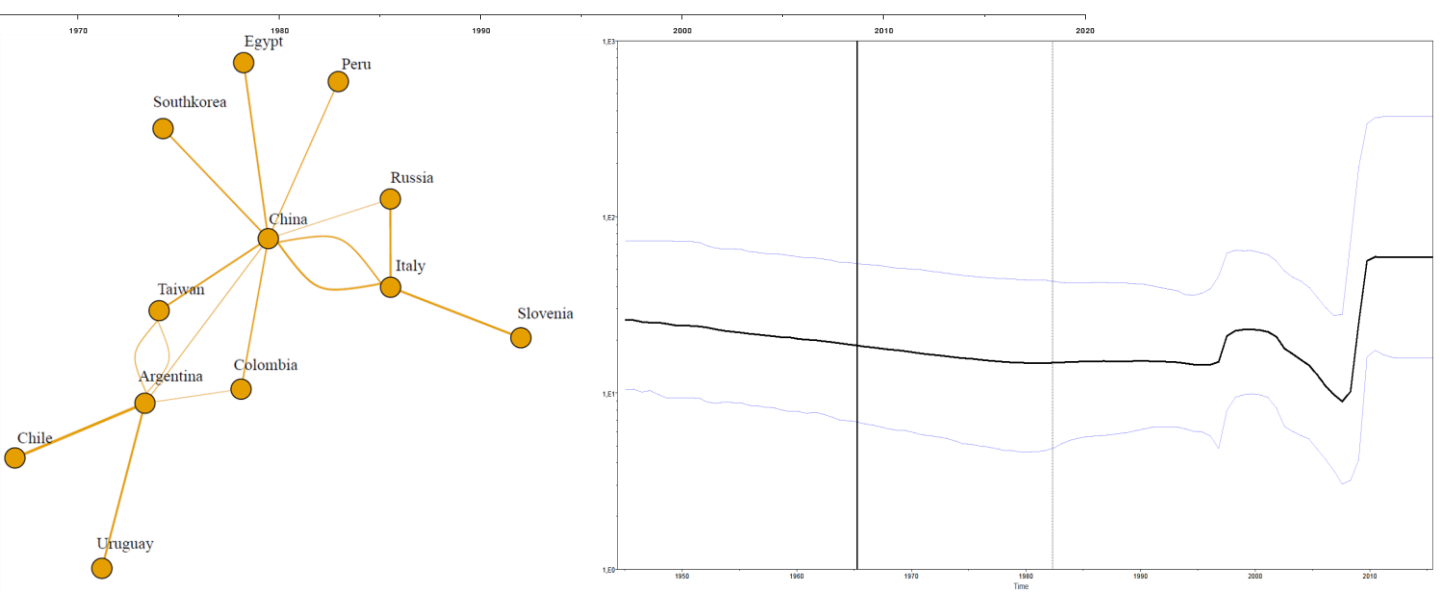

Supplement: S2 Fig — Time calibrated phylogentic trees calculated using the HVR12. The tree branches have been color-coded according to the location predicted with the highest posterior probability (reported near the respective node). Left insert: Network reporting the well supported (BF>3) GI-16 spreading path among different countries (the arrows size is proportional to the BF value). Right insert: Mean and 95HPD relative genetic diversity (Ne x t) of the worldwide GI-16 population over time. (PDF) [file pone.0203513.s002.pdf]
