# Supplementary material for: GI-16 lineage (624/I or Q1), there and back again: The history of one of the major threats for poultry farming of our era
Source: PLoS One. 2018 Dec 20;13(12):e0203513. doi: 10.1371/journal.pone.0203513 (PMC6301571; doi:10.1371/journal.pone.0203513)

1

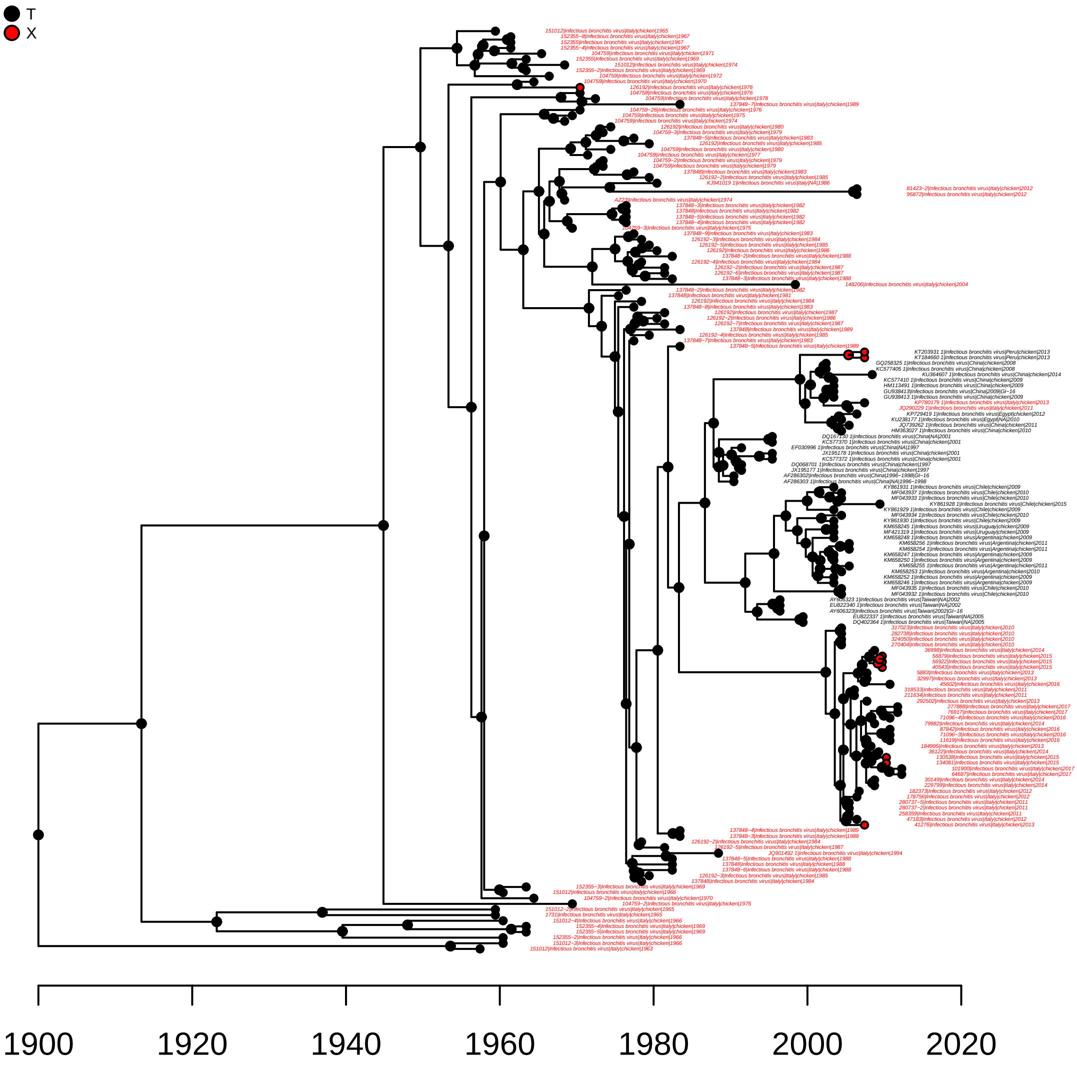

2

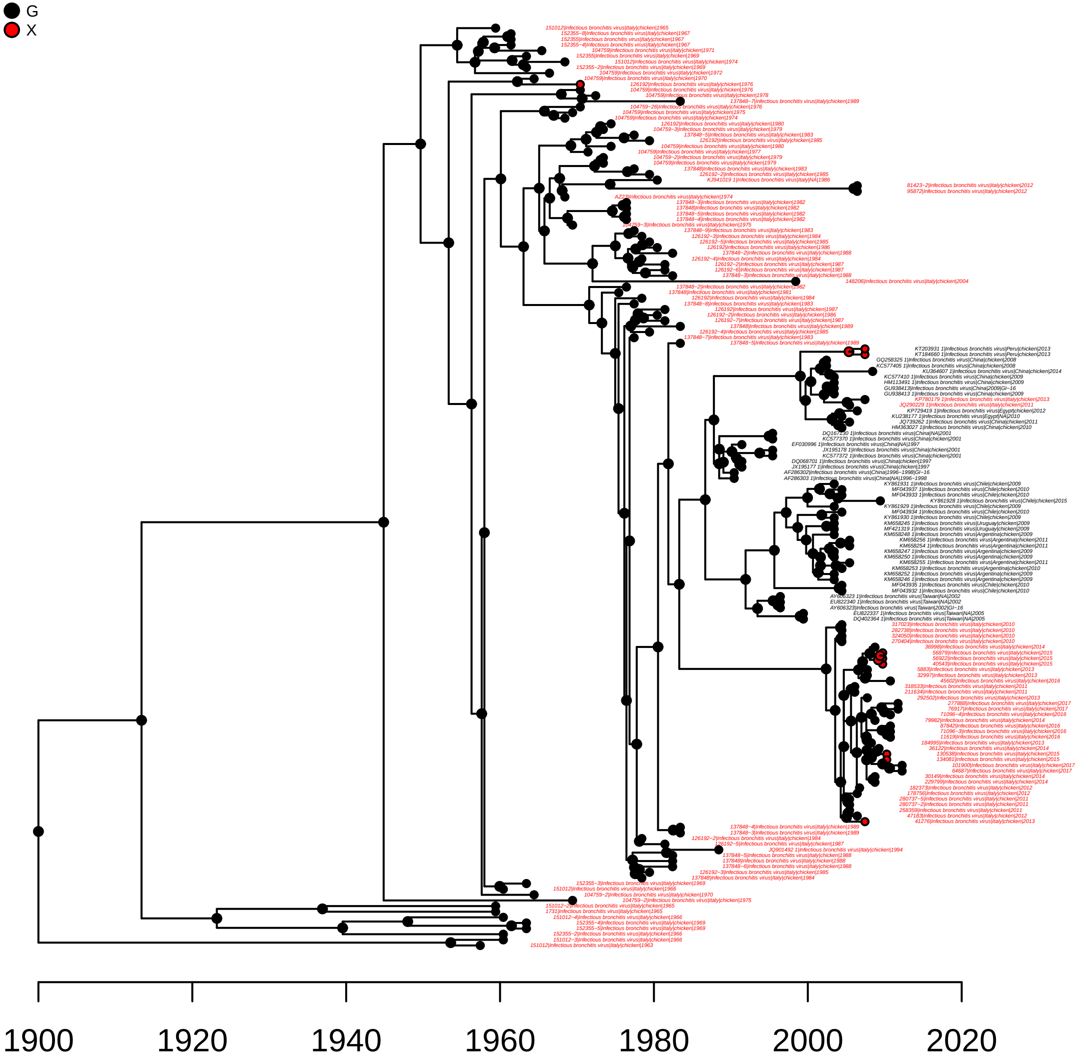

3

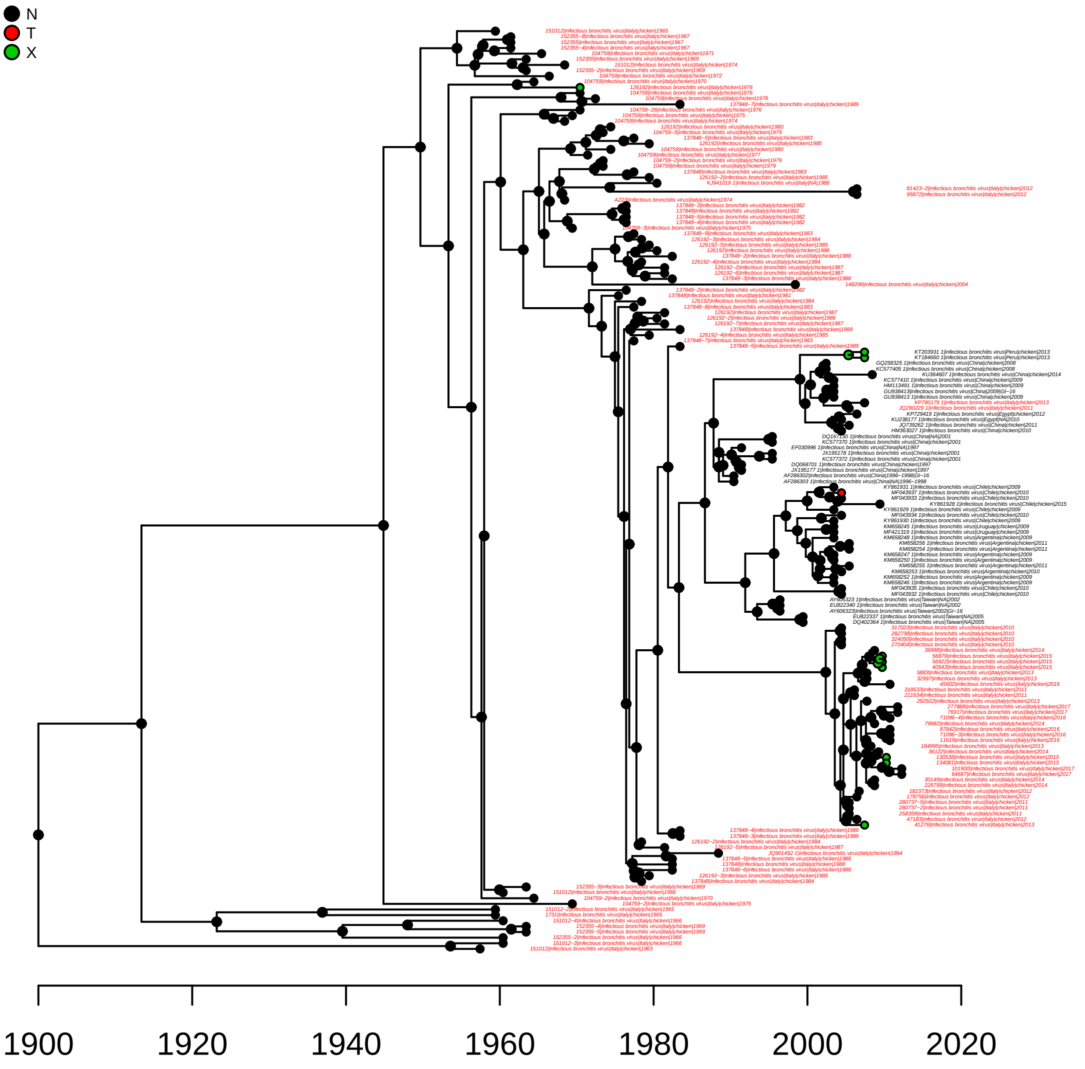

4

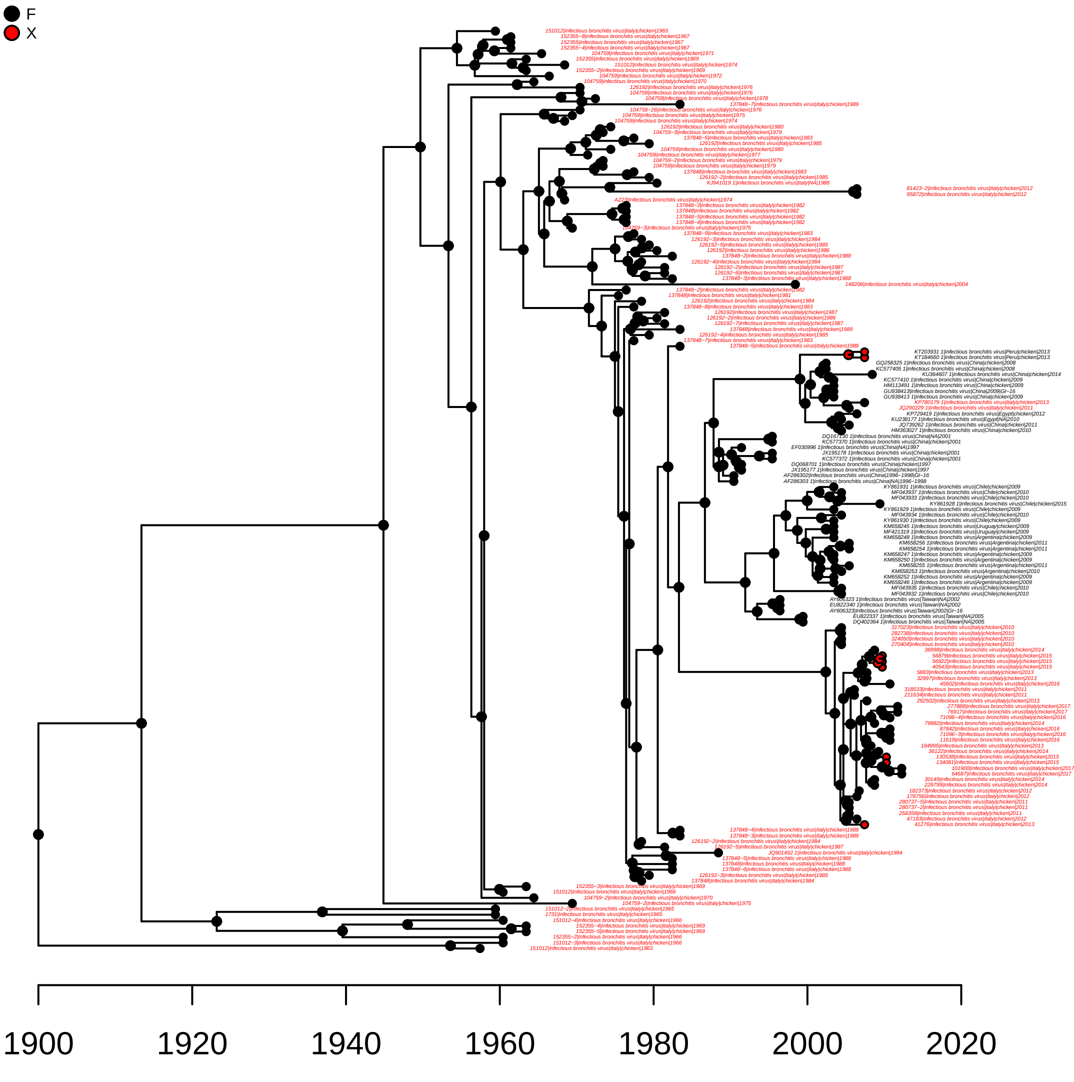

5

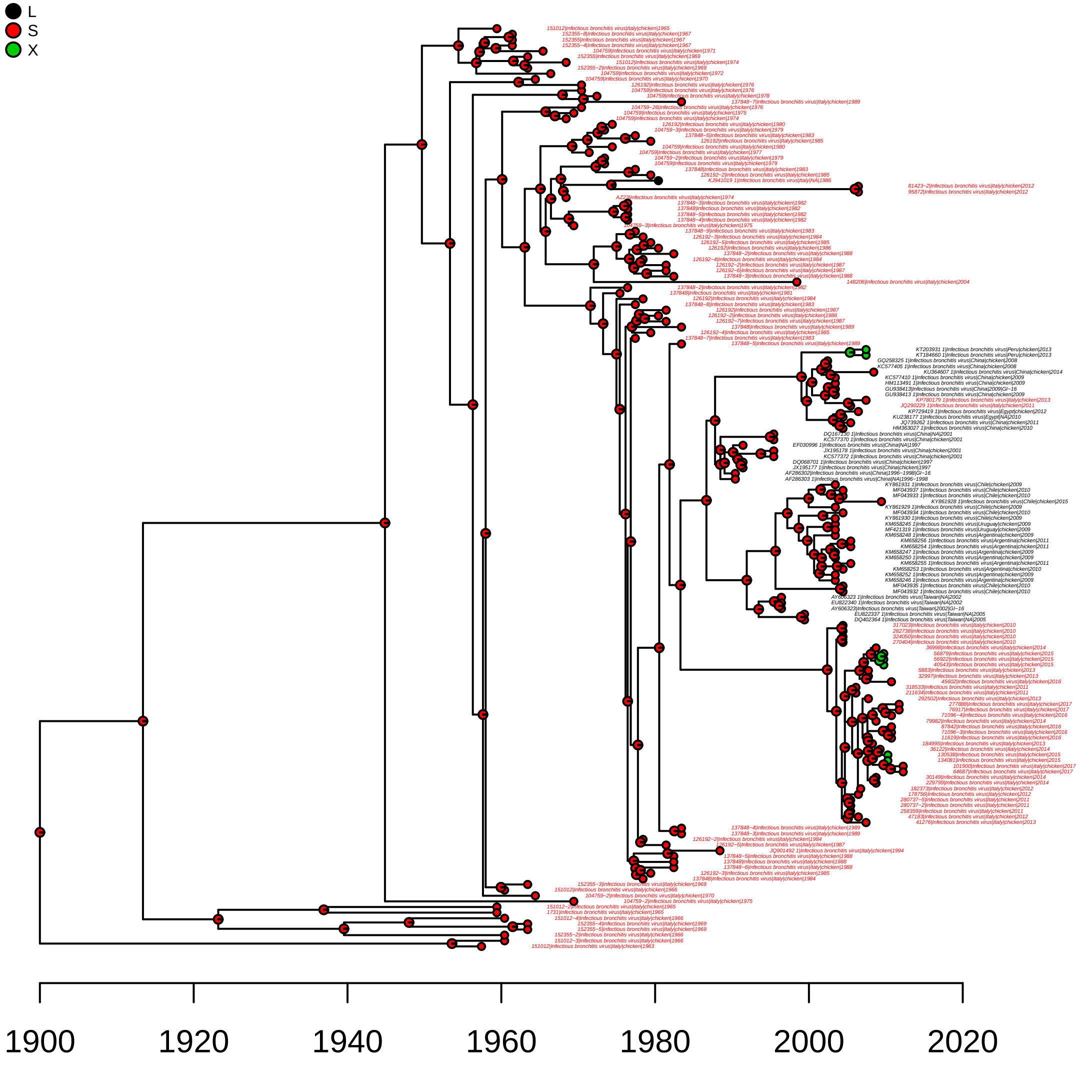

6

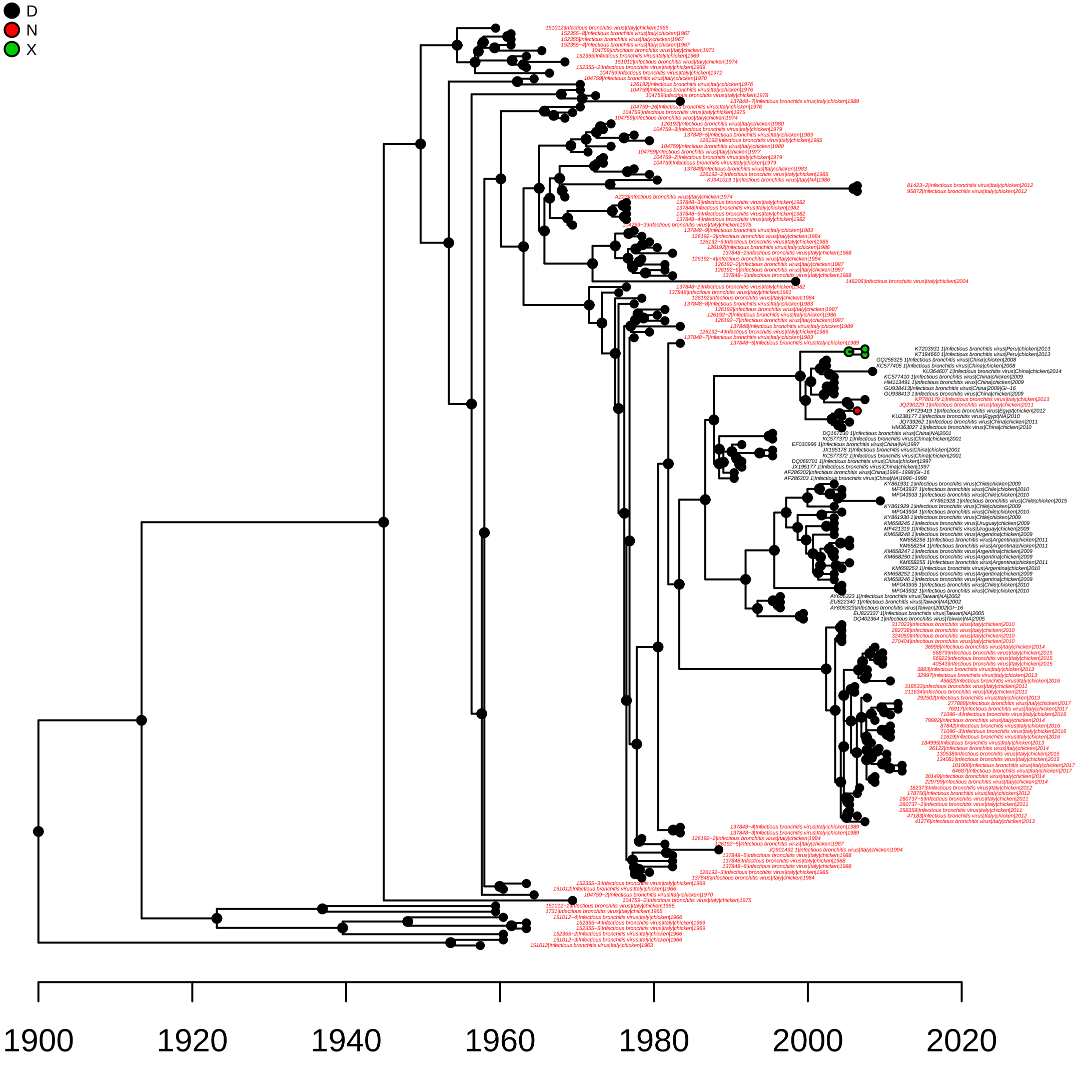

7

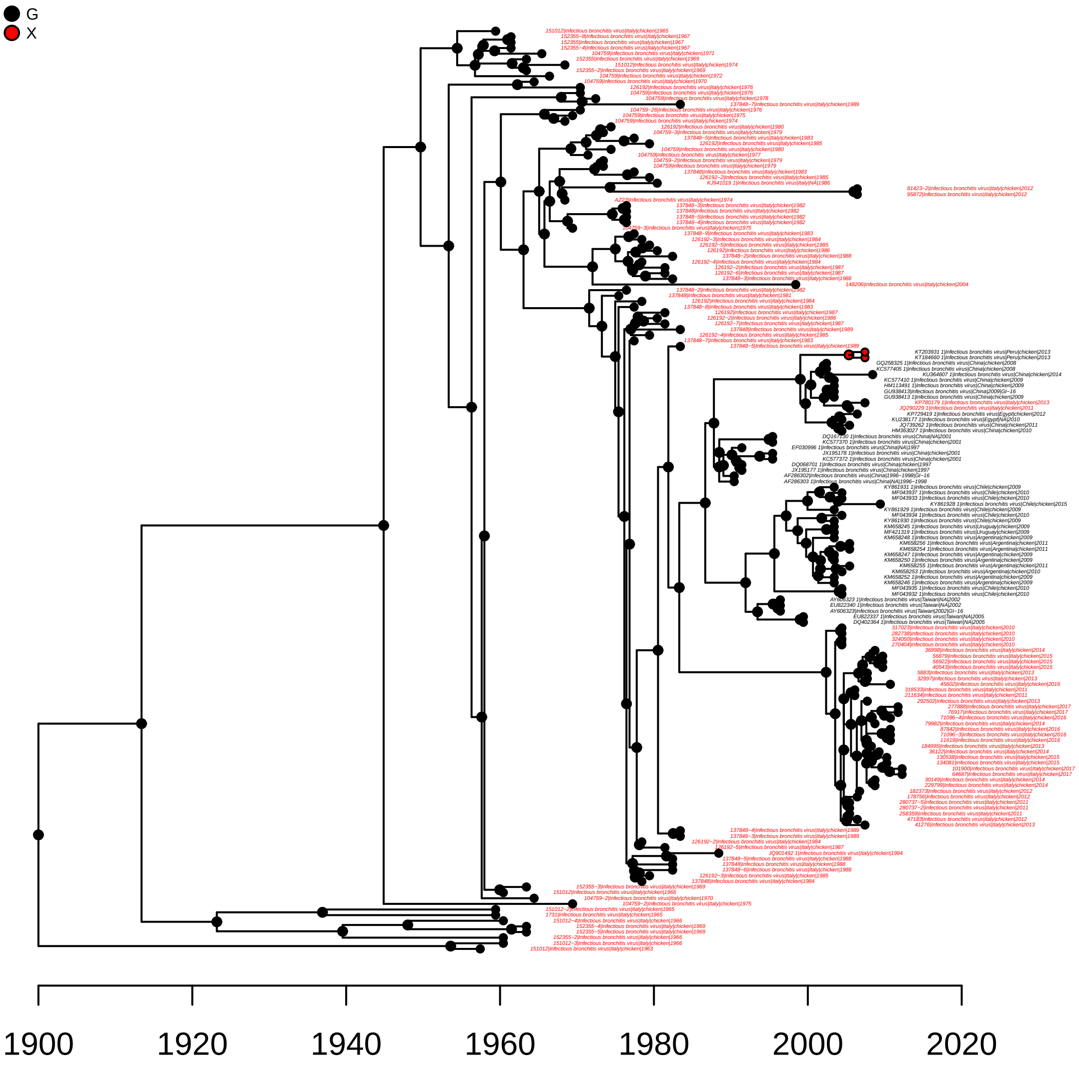

8

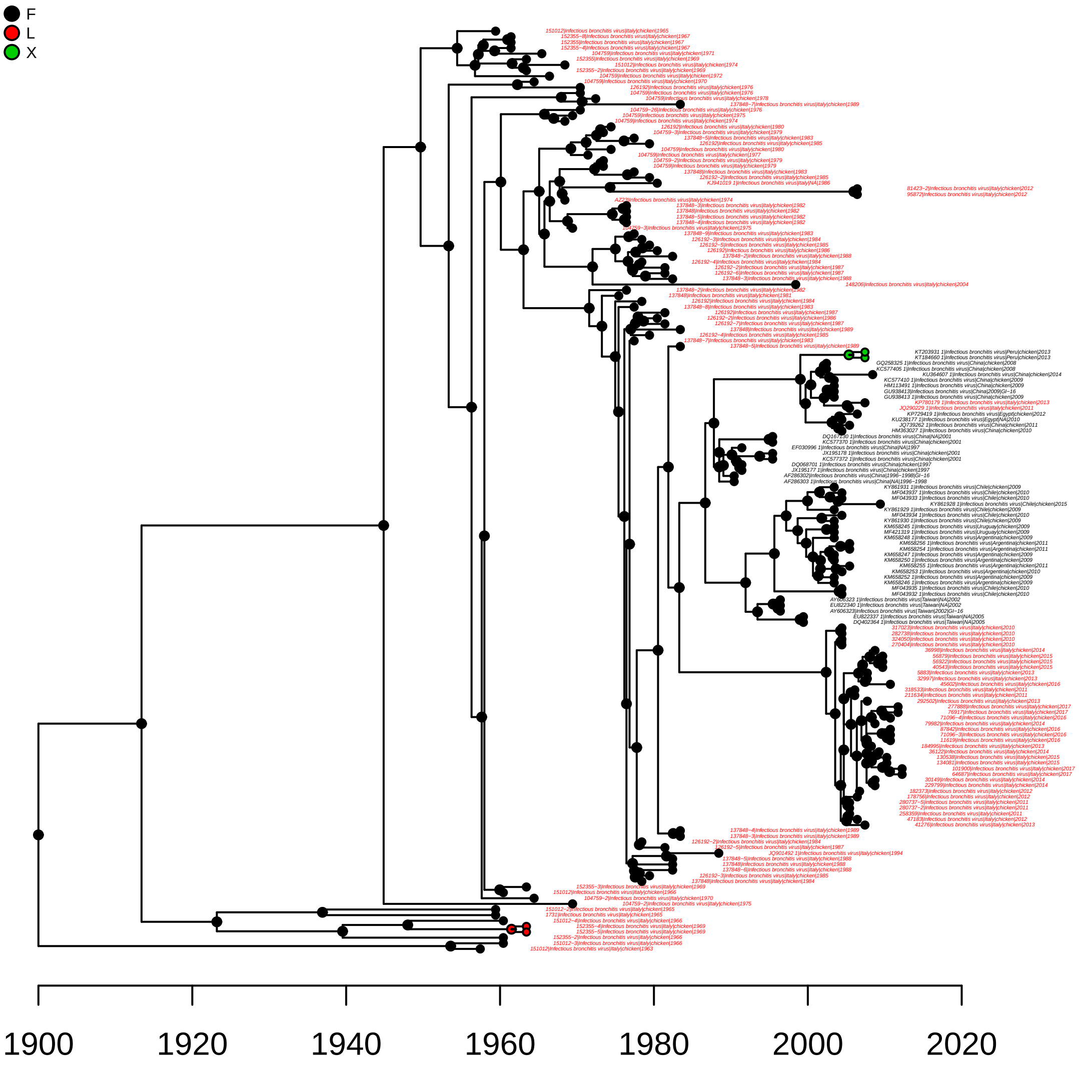

9

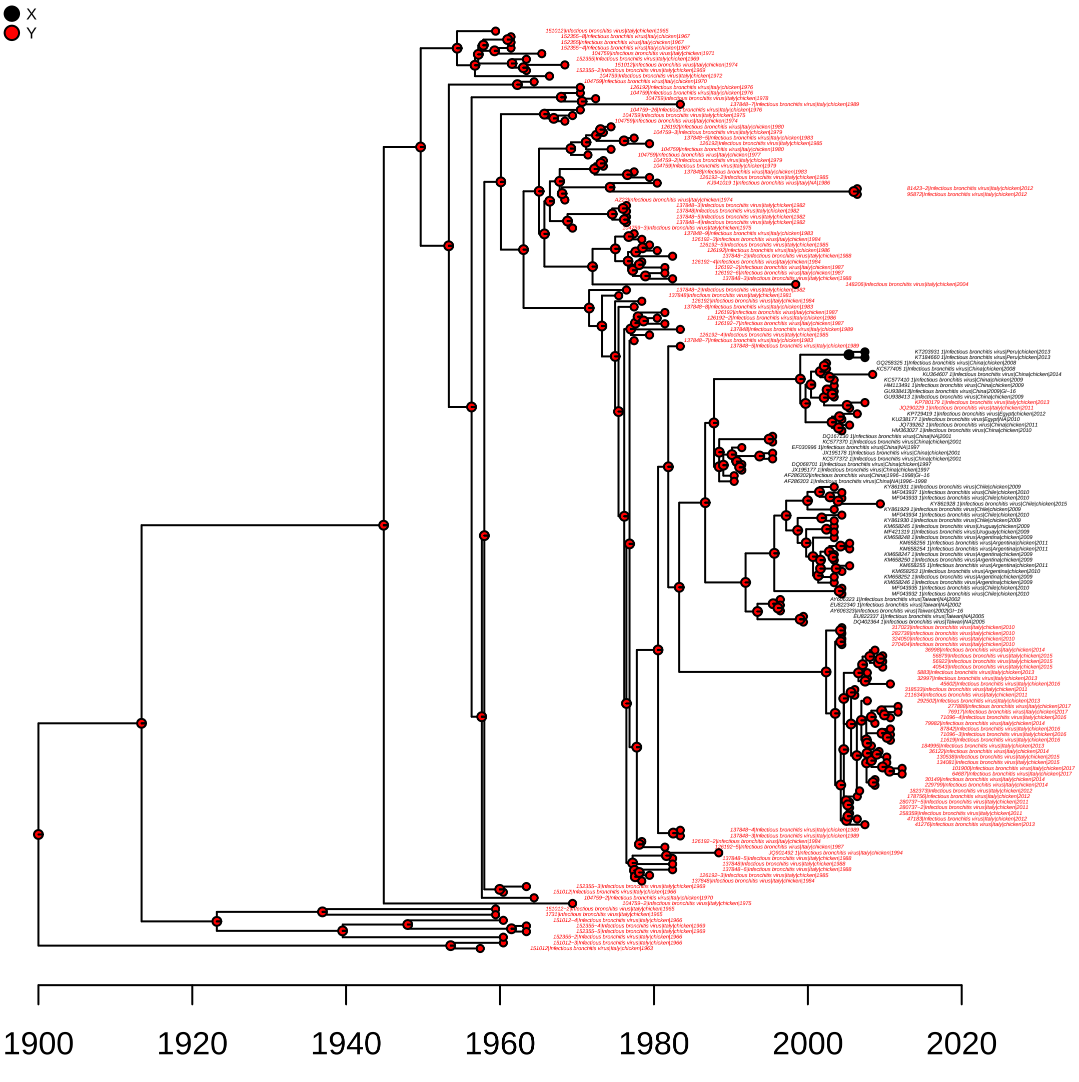

10

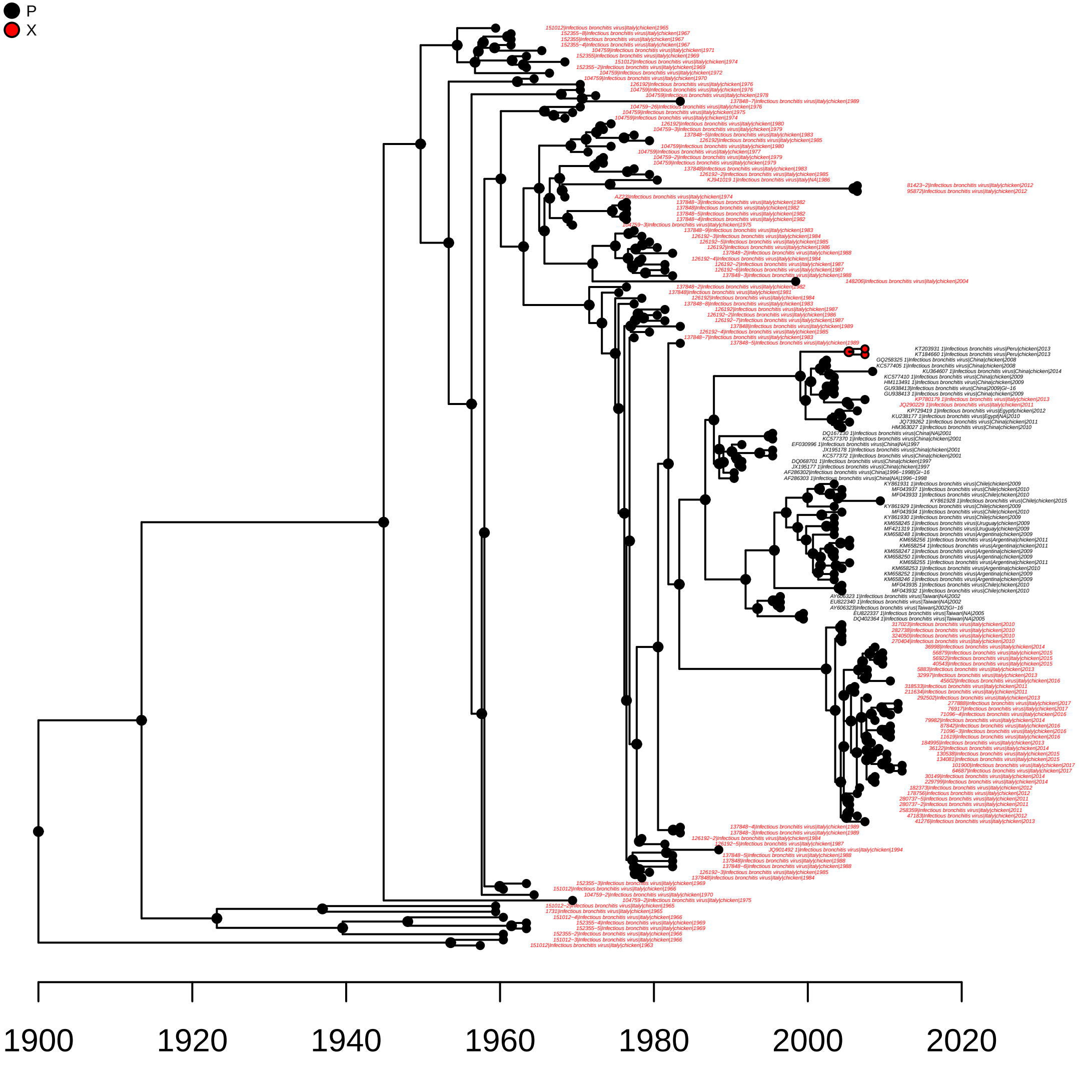

11

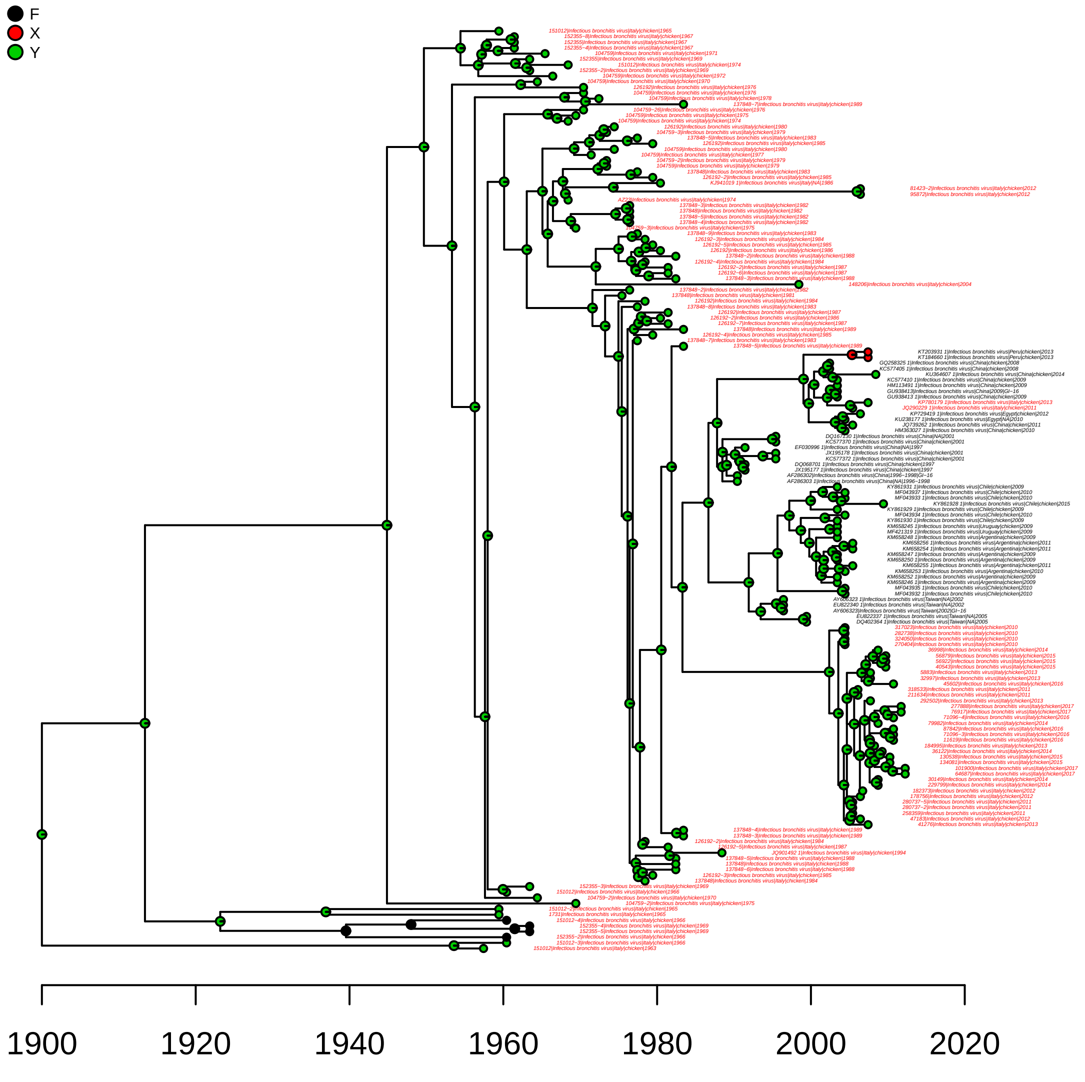

12

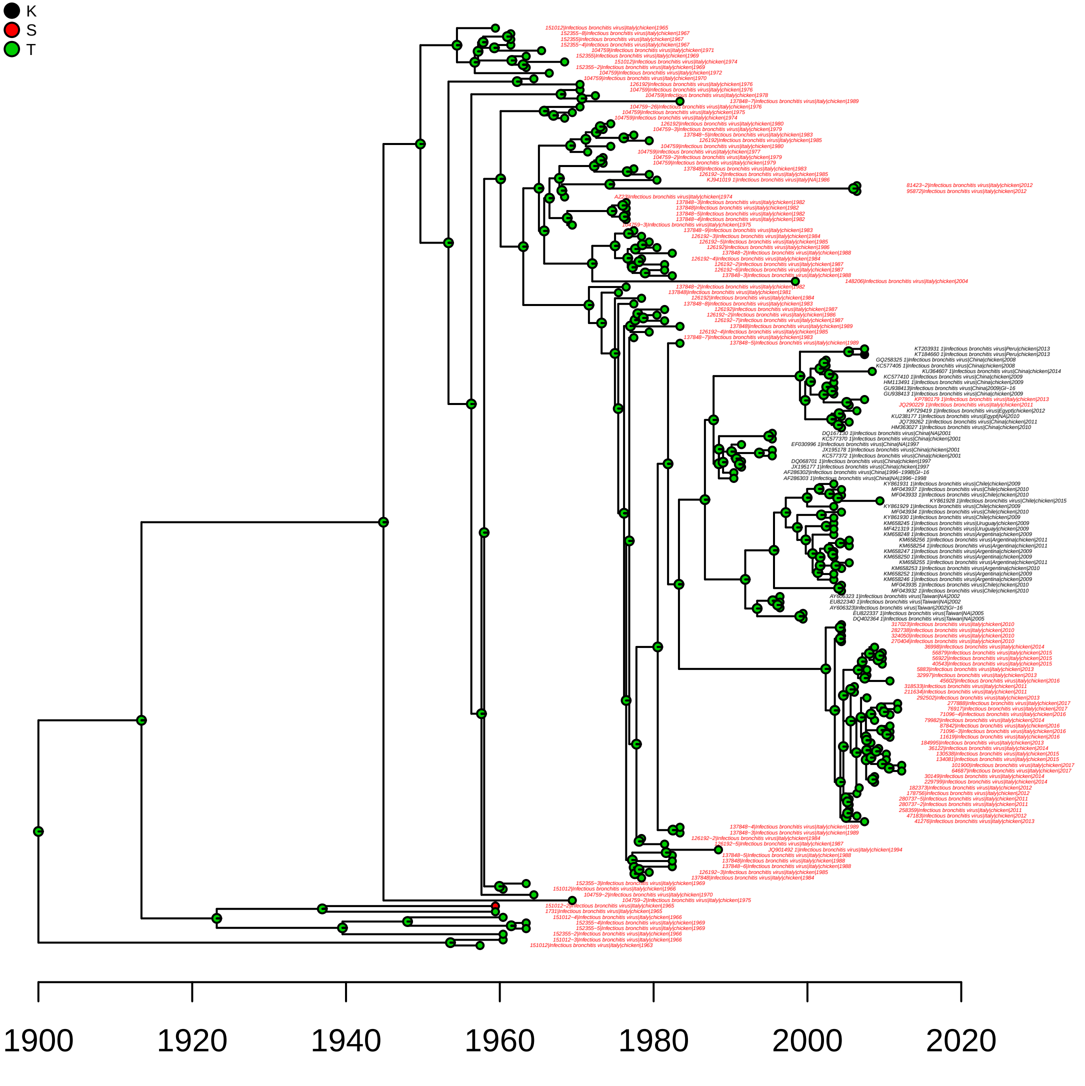

15

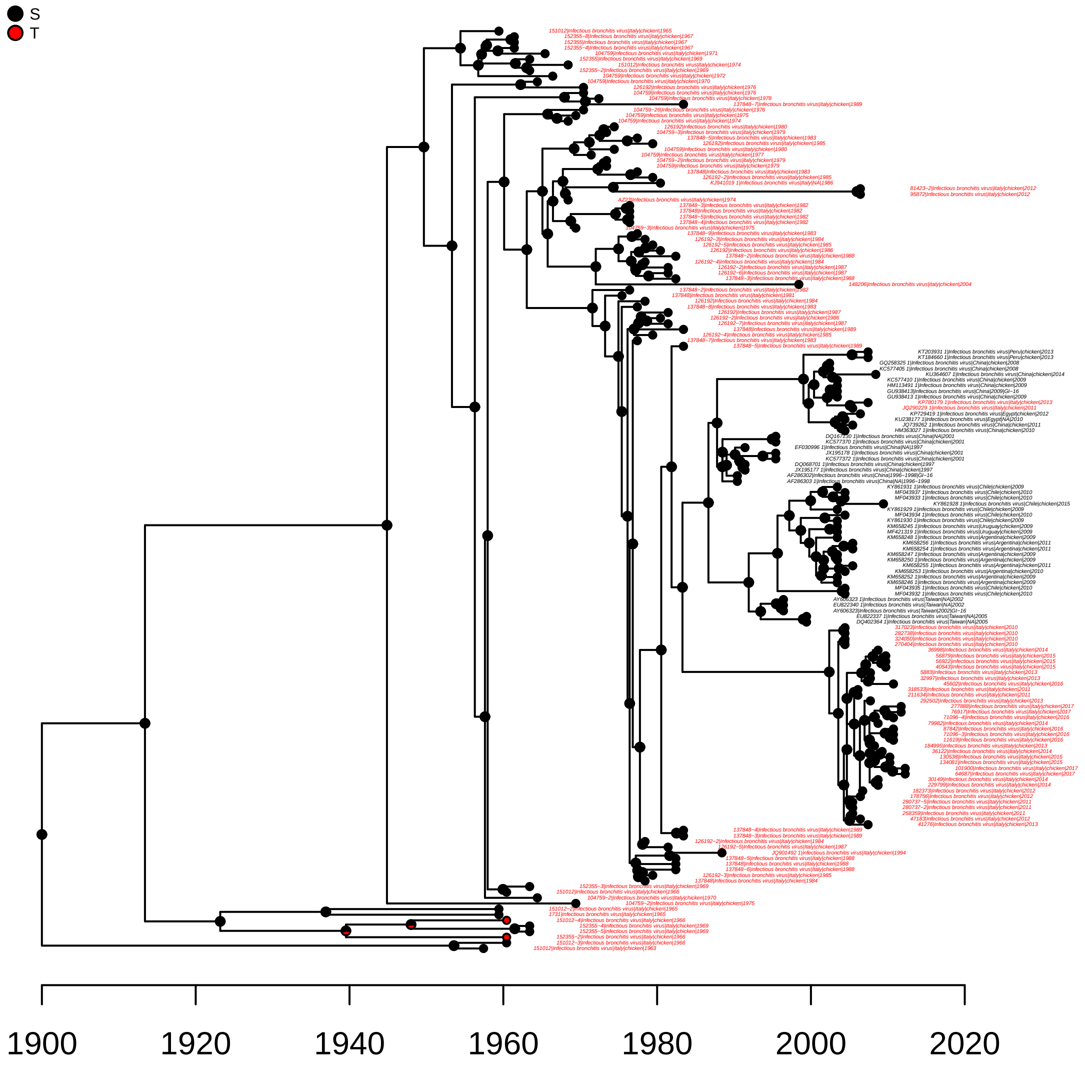

16

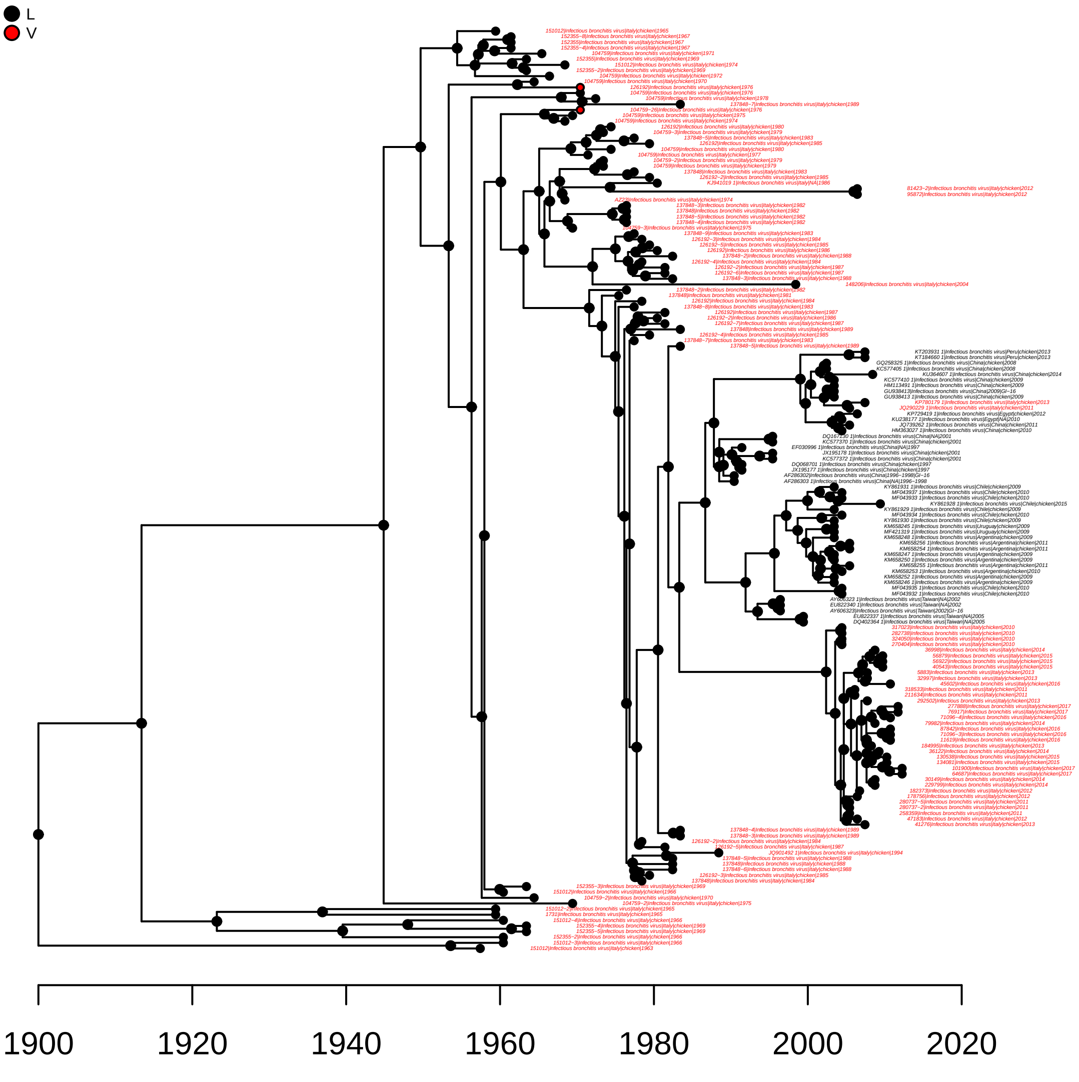

17

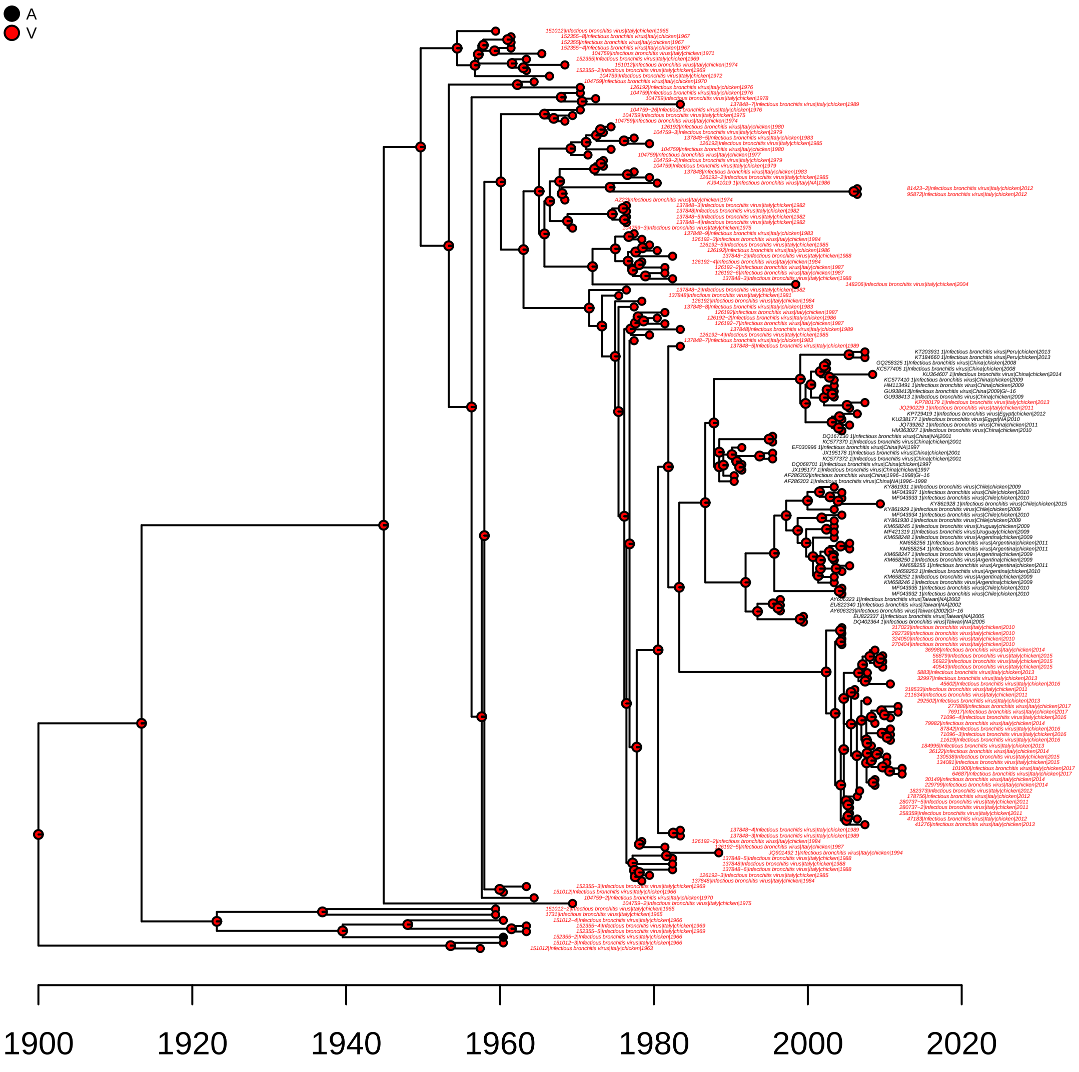

18

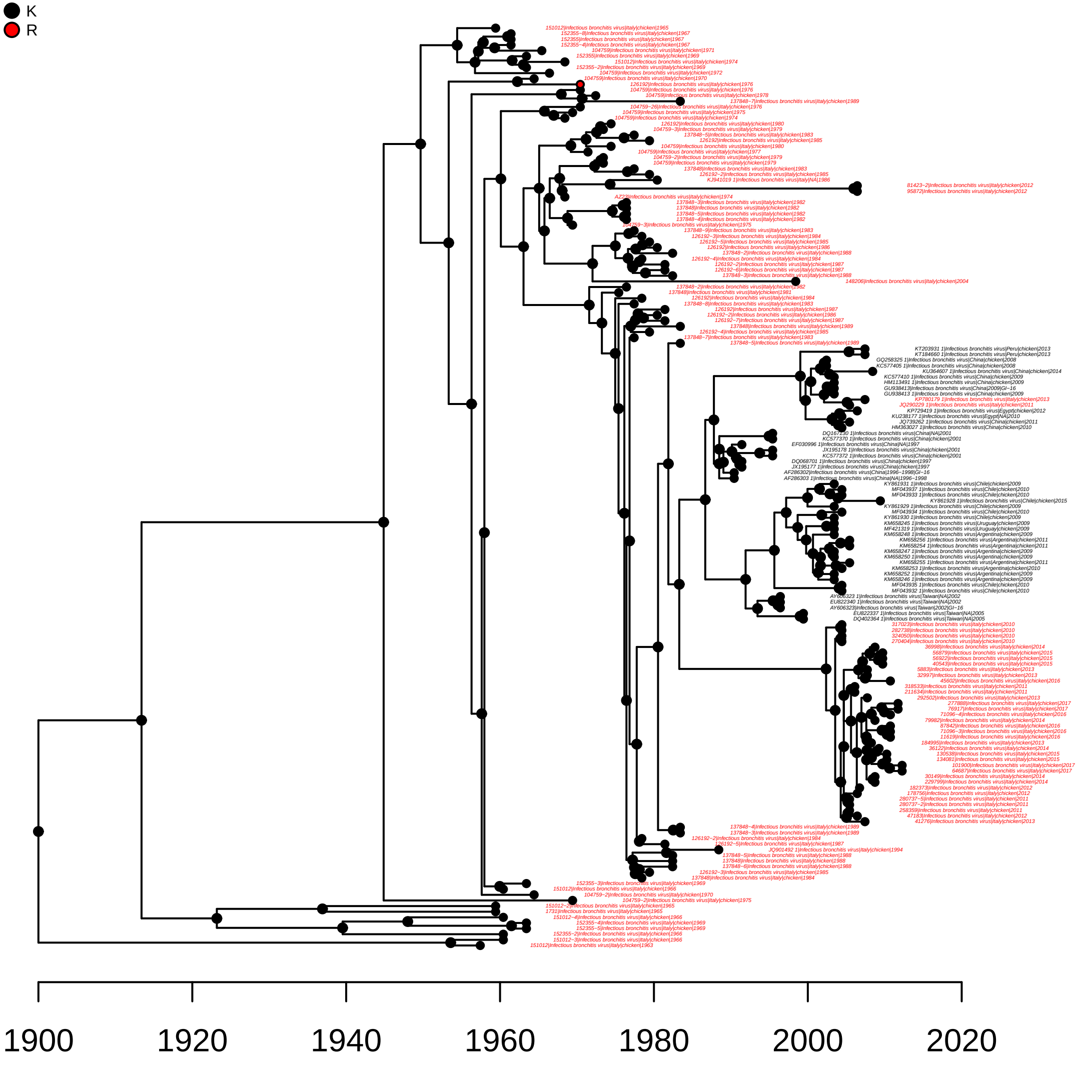

19

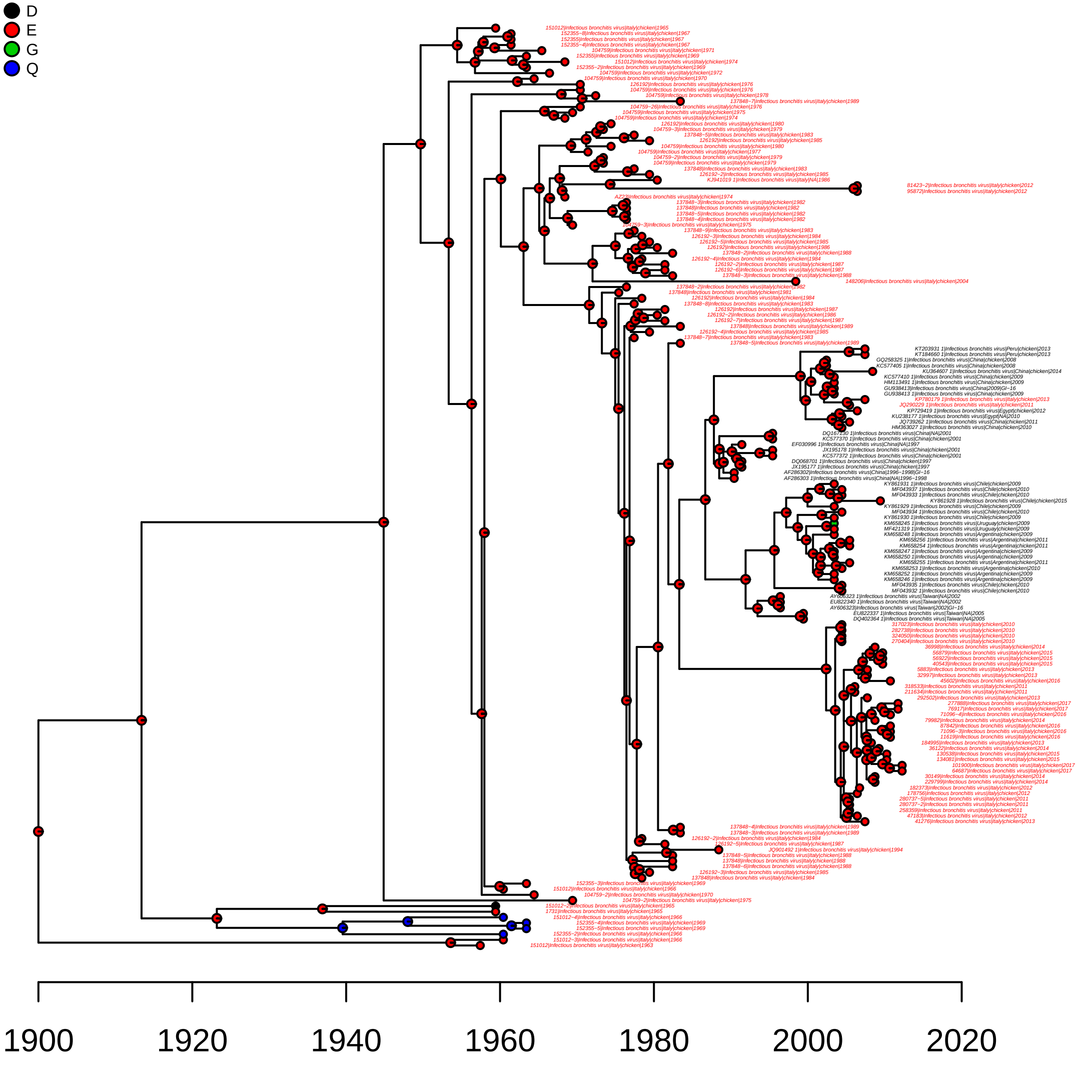

20

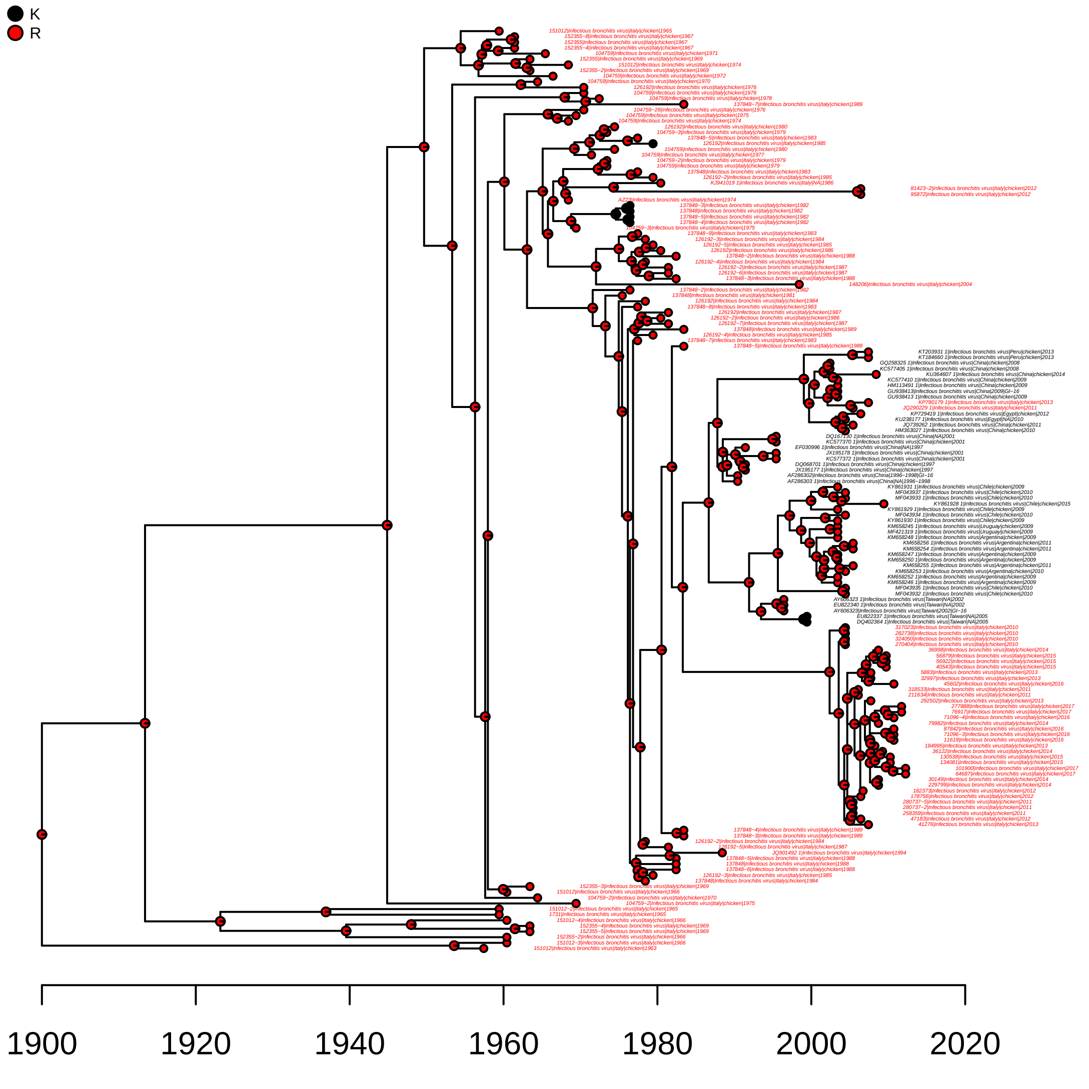

22

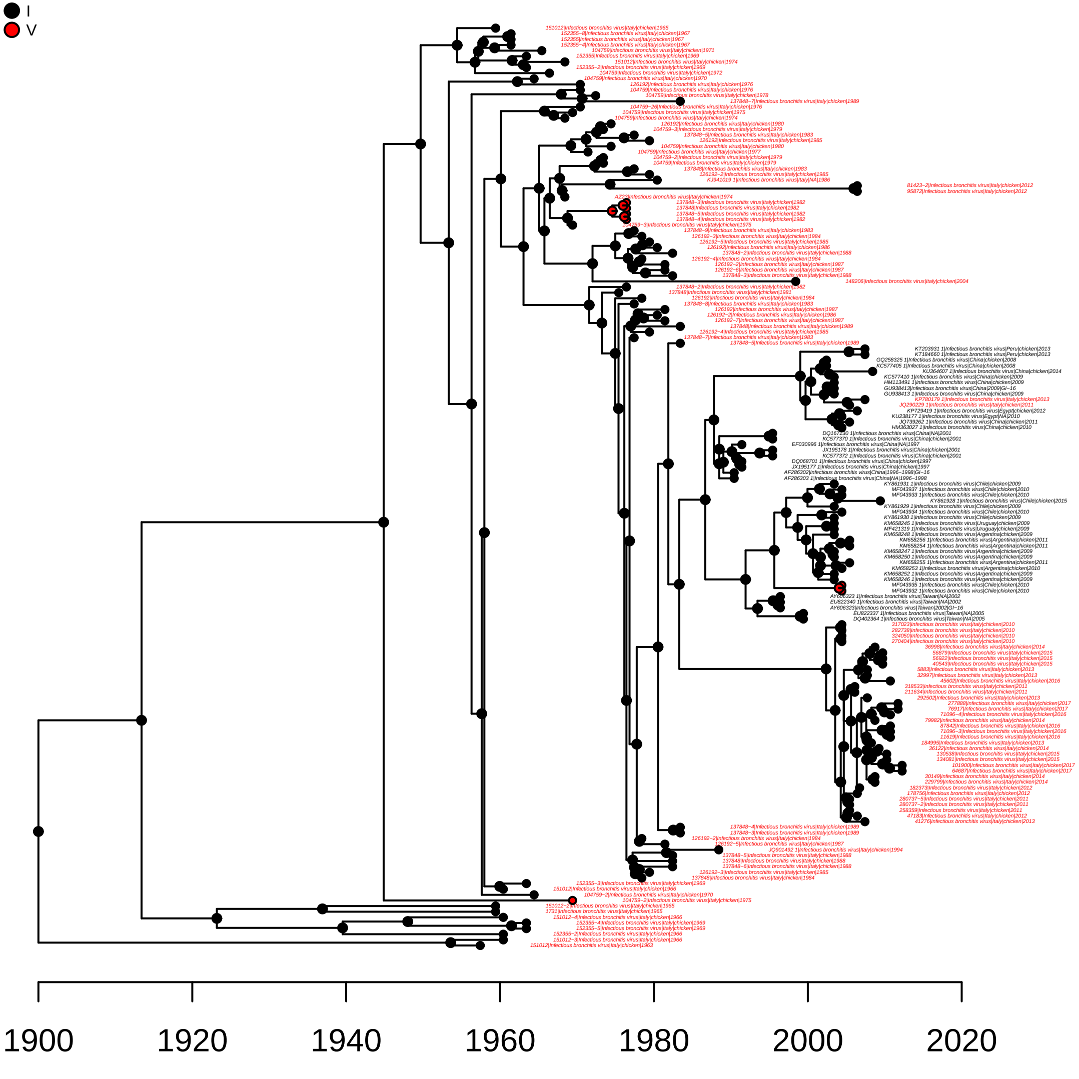

24

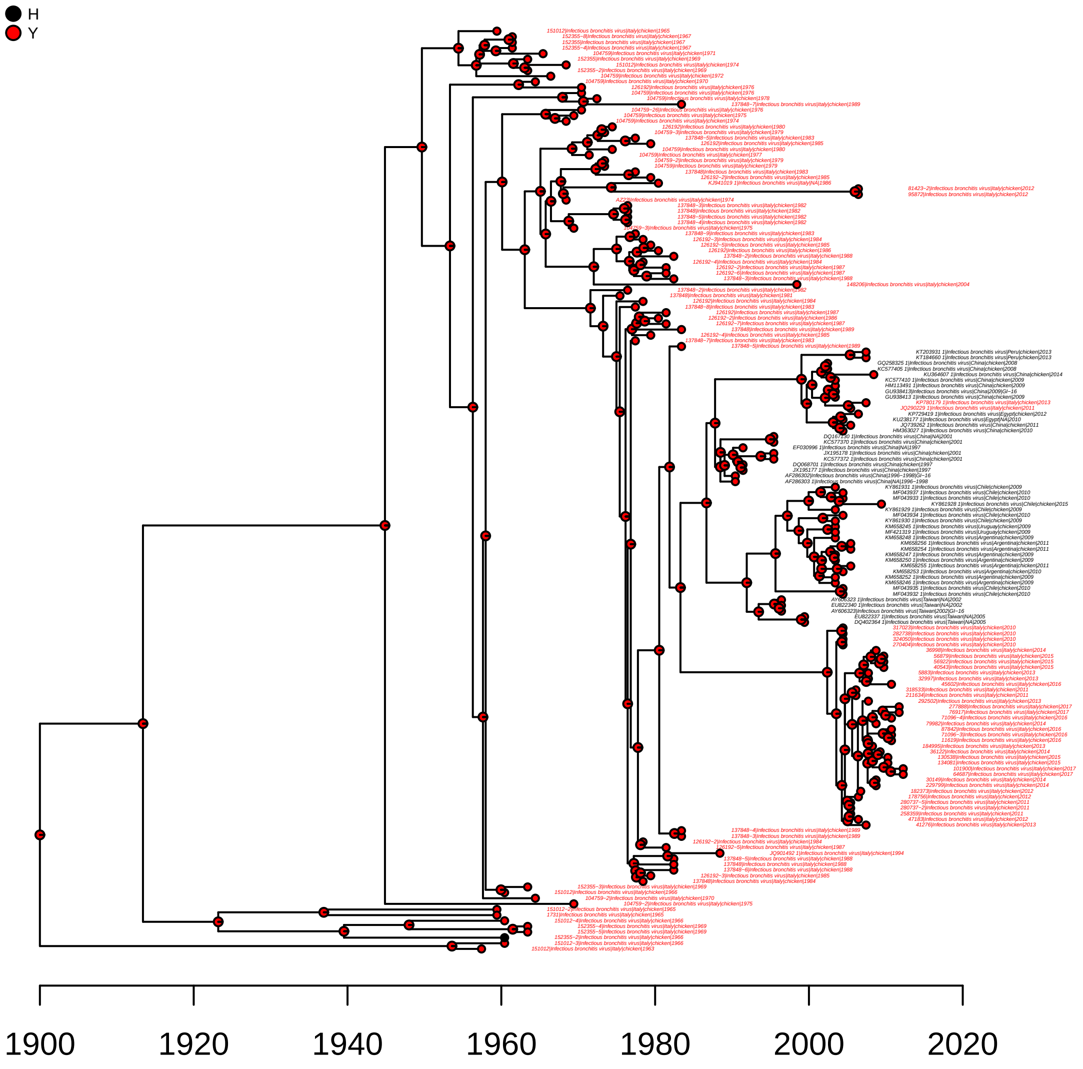

27

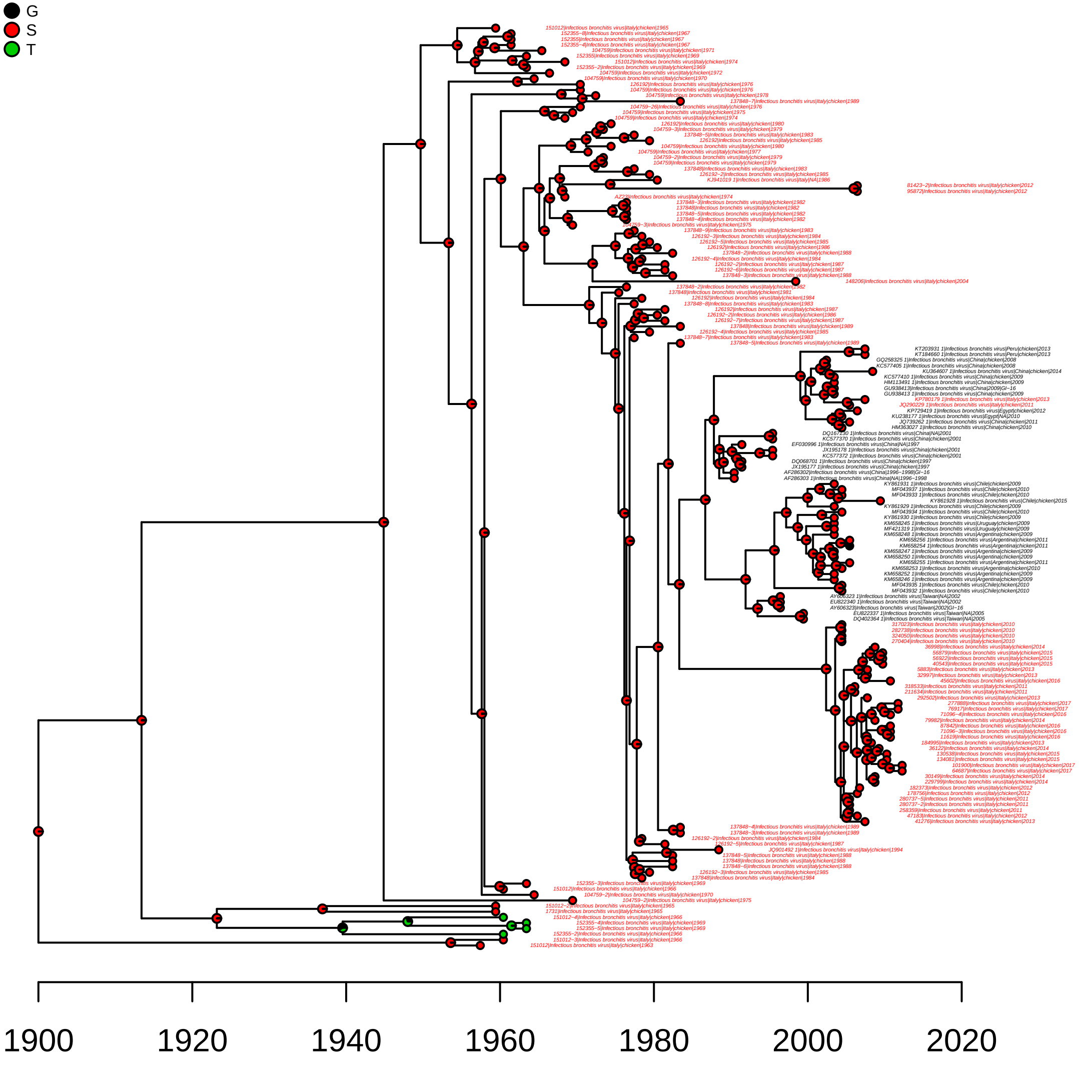

28

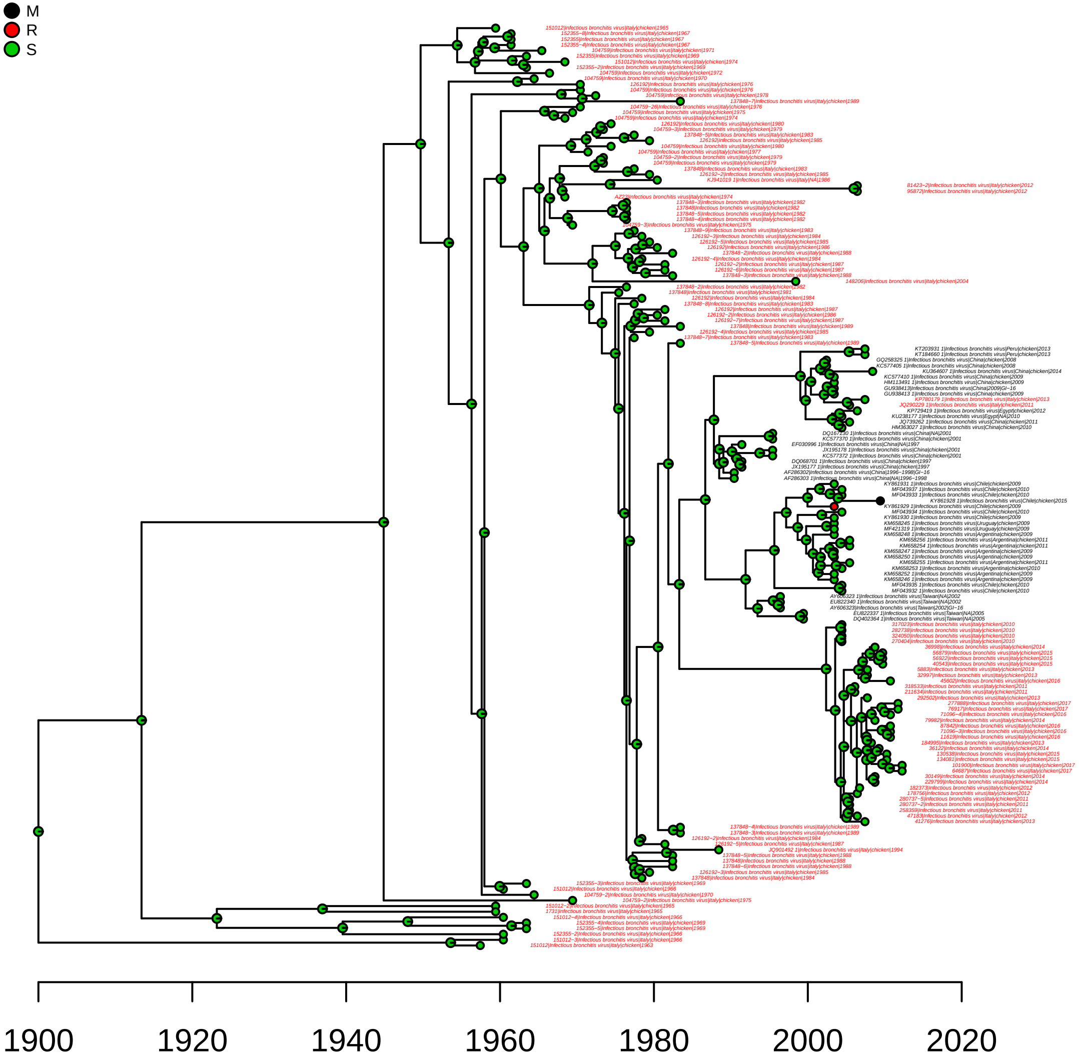

29

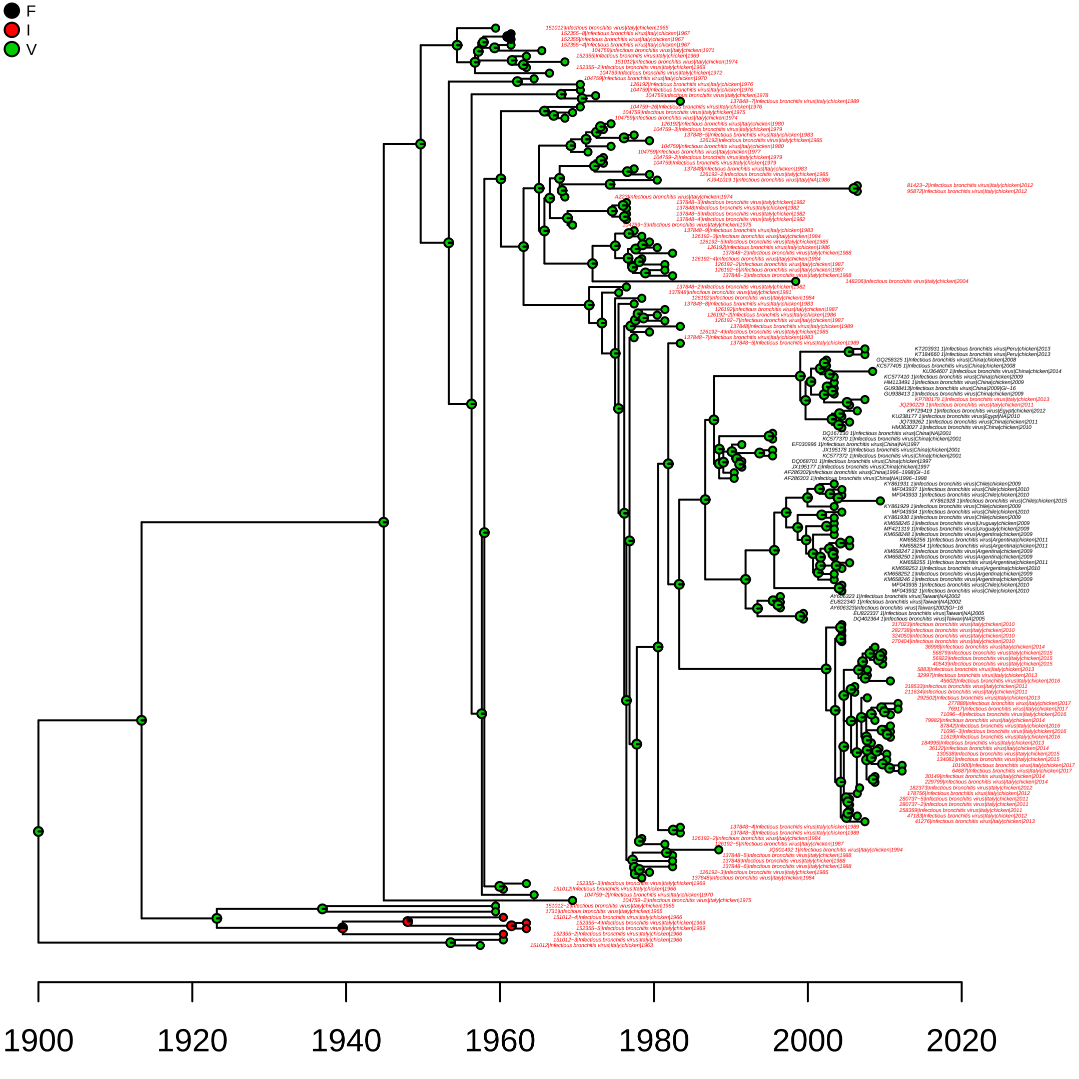

30

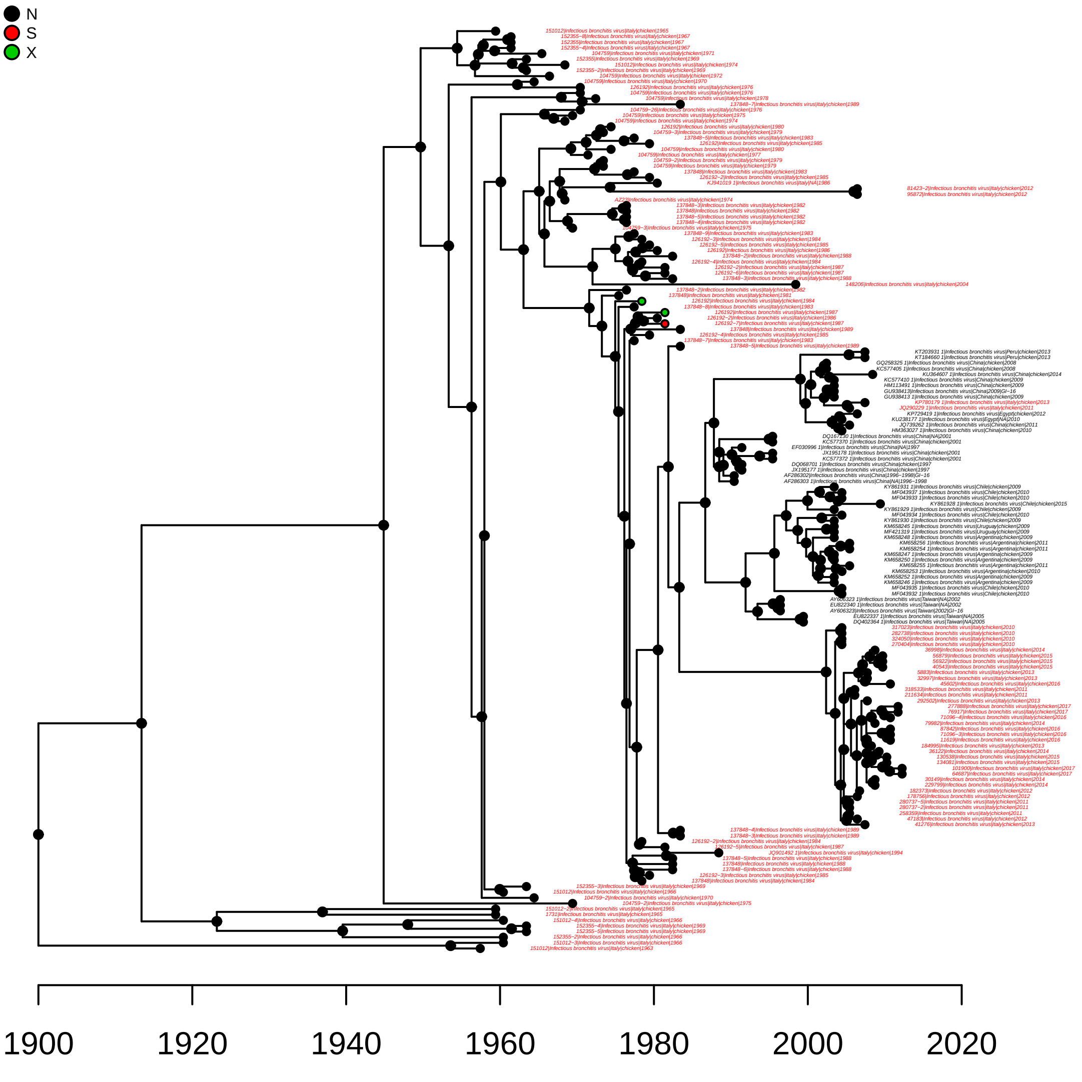

32

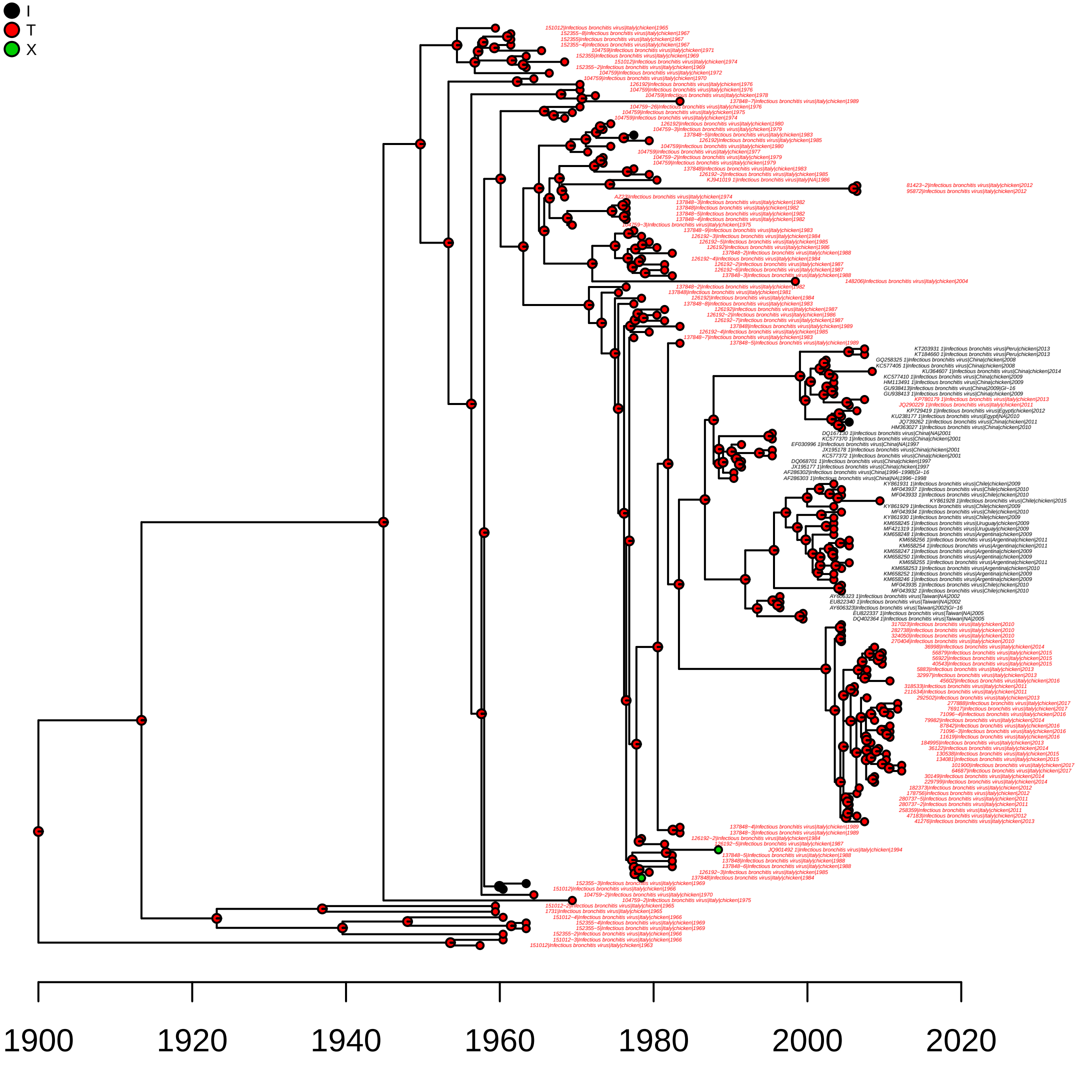

34

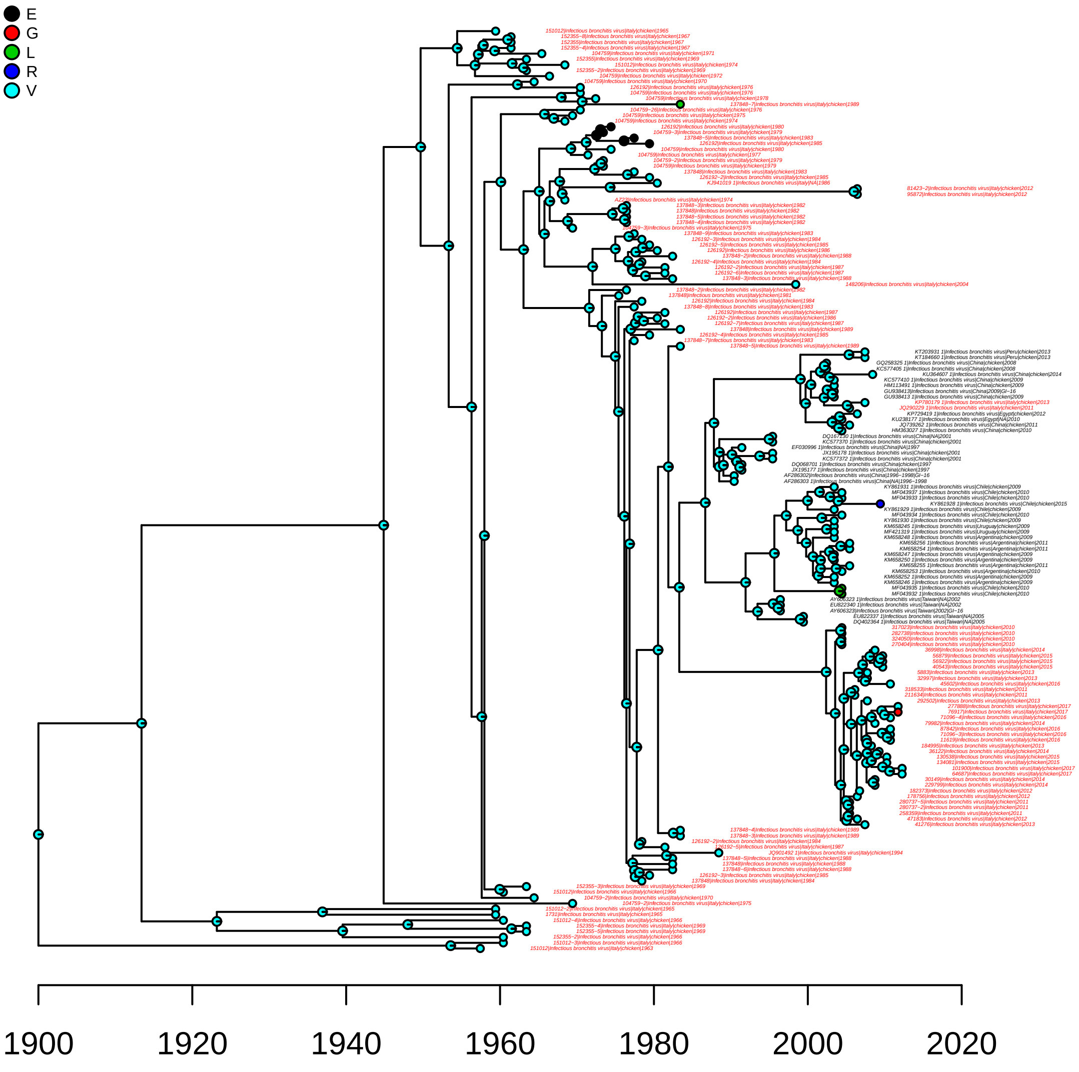

35

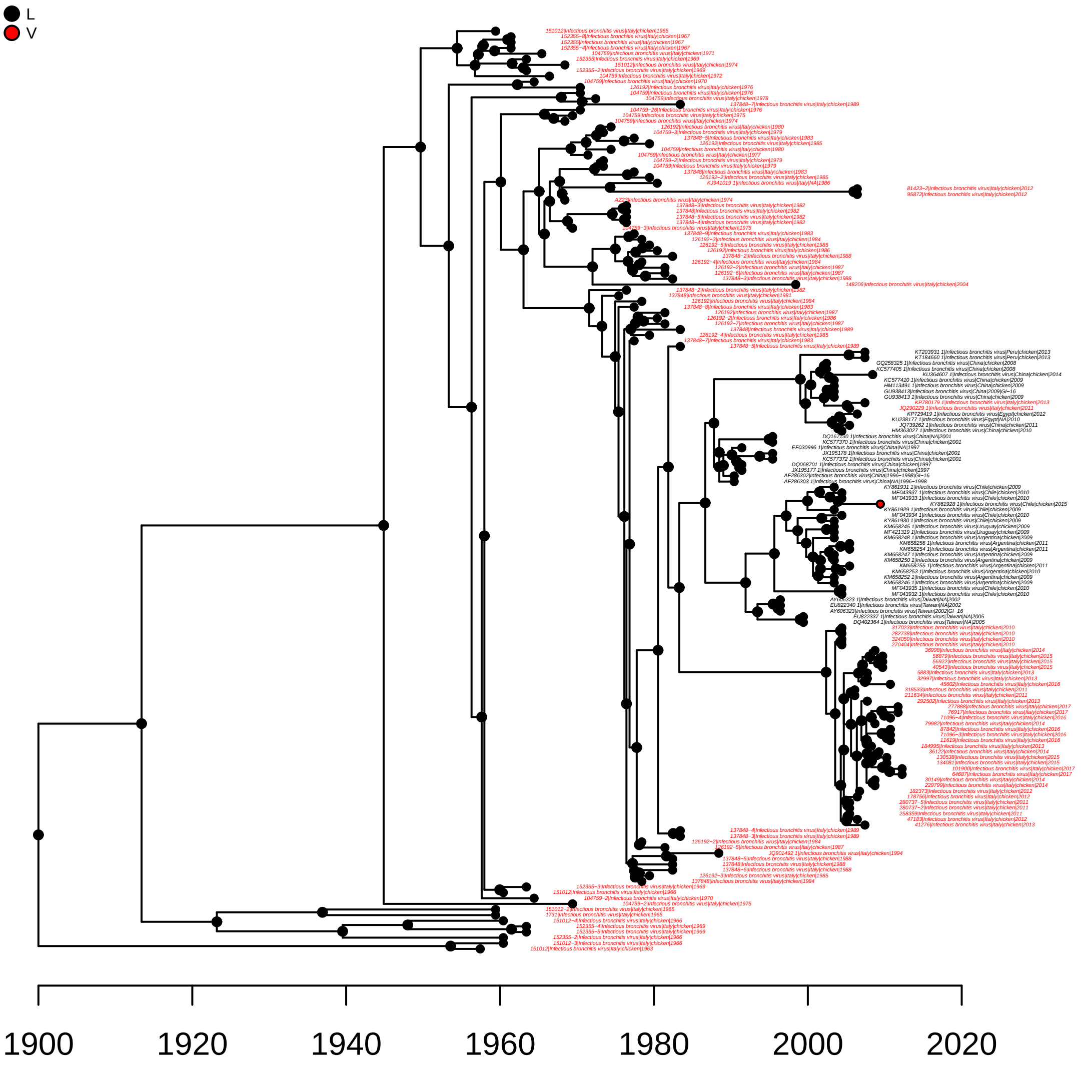

36

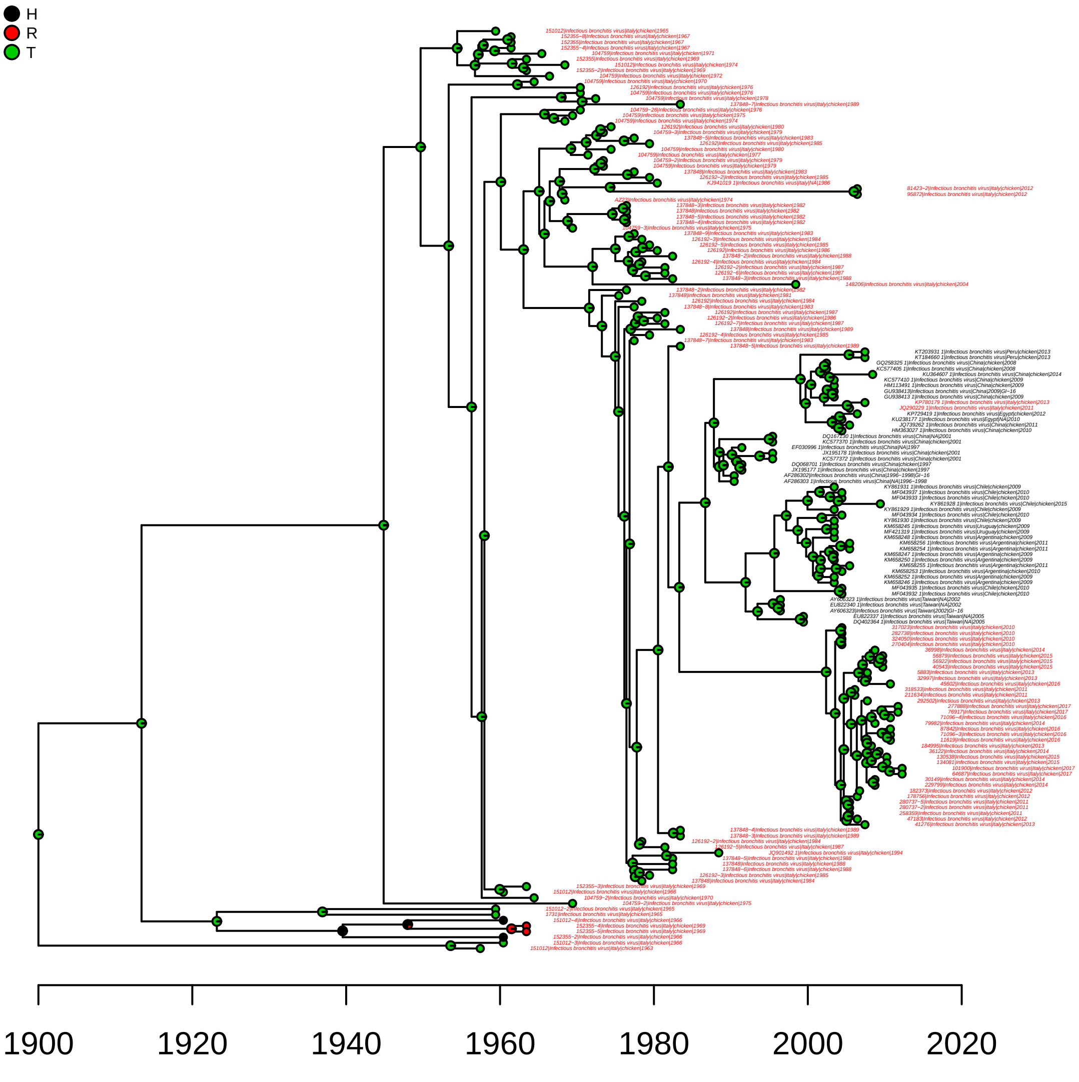

38

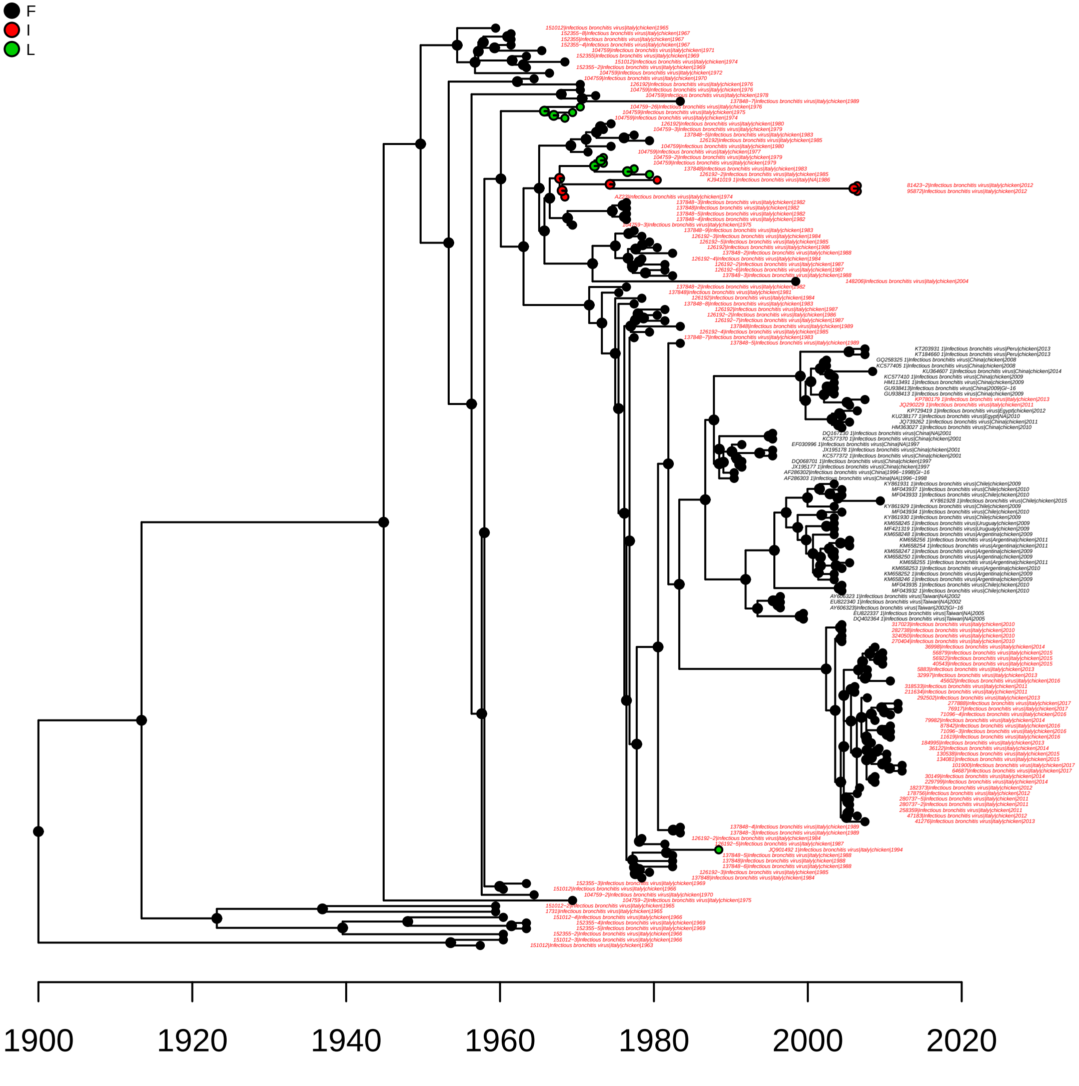

43

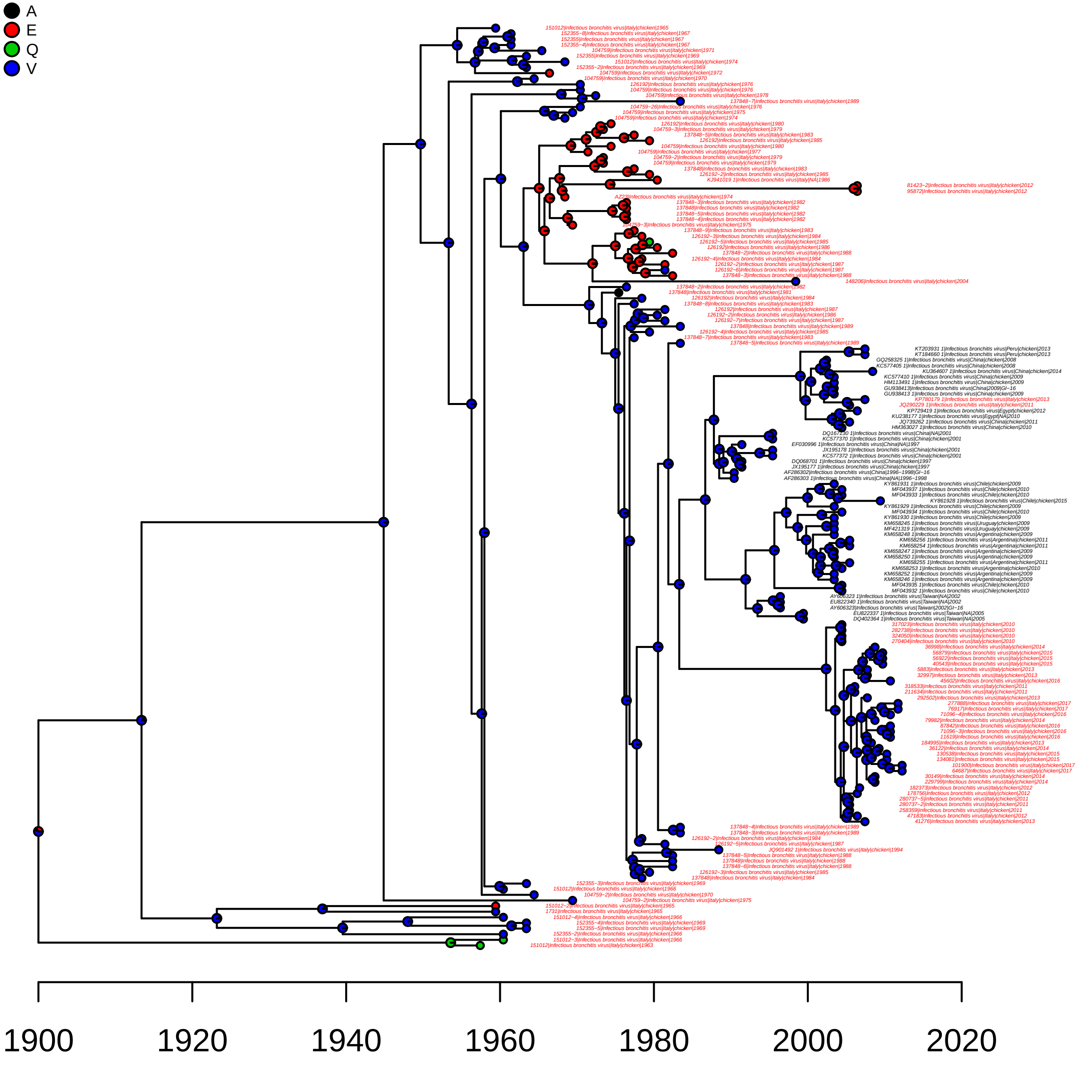

44

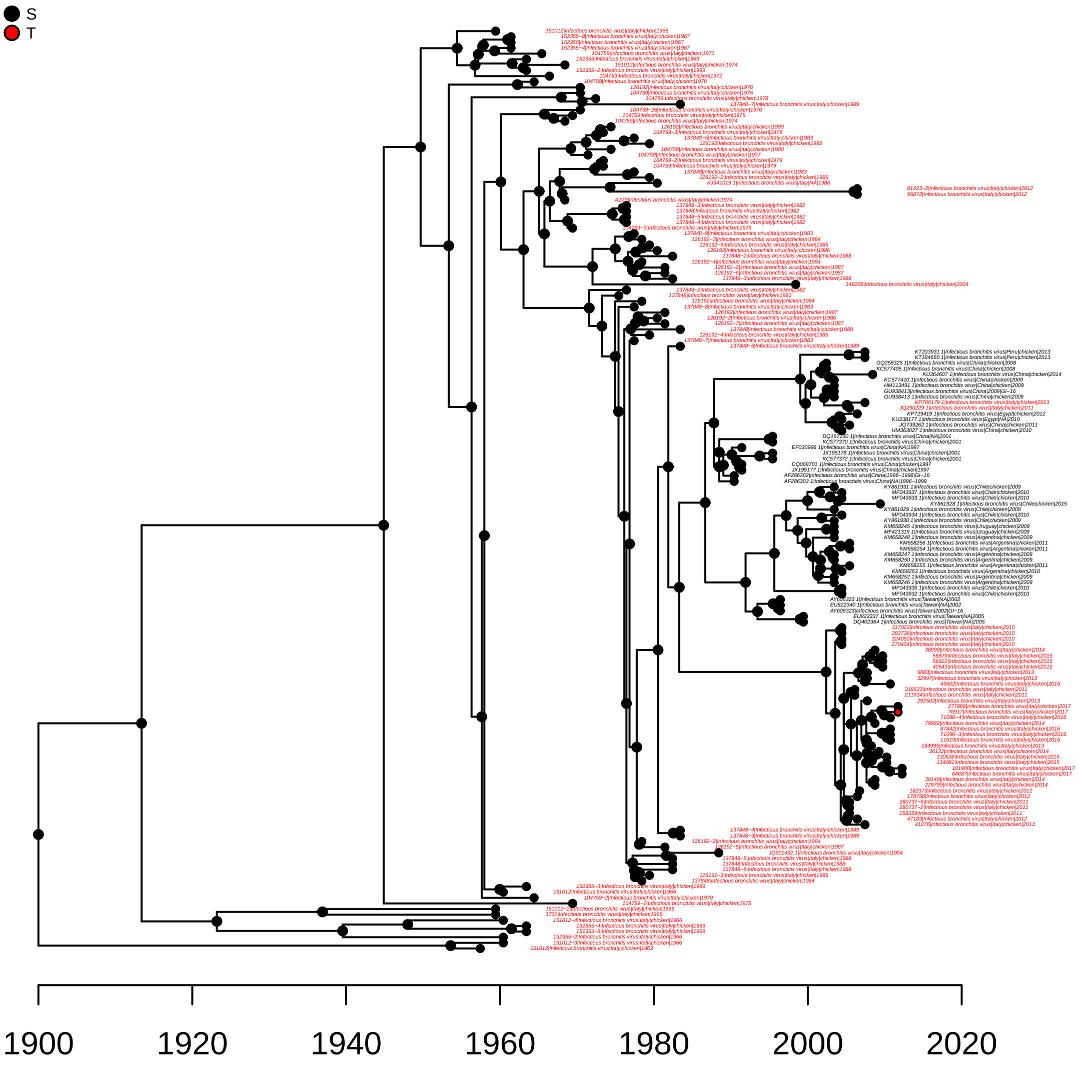

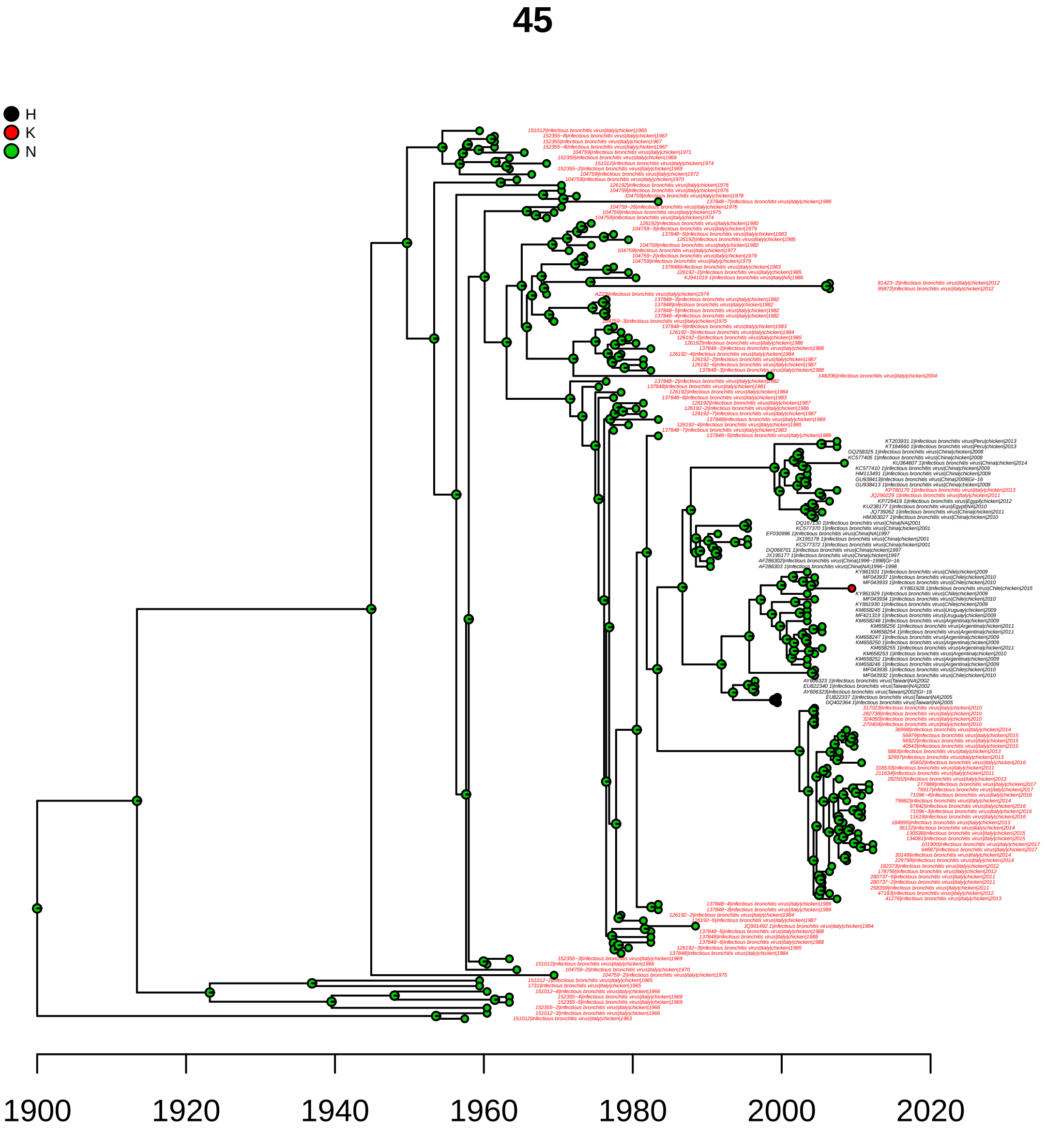
45
H
K
N
1900
1920
1940
1960
1980
2000
2020

46

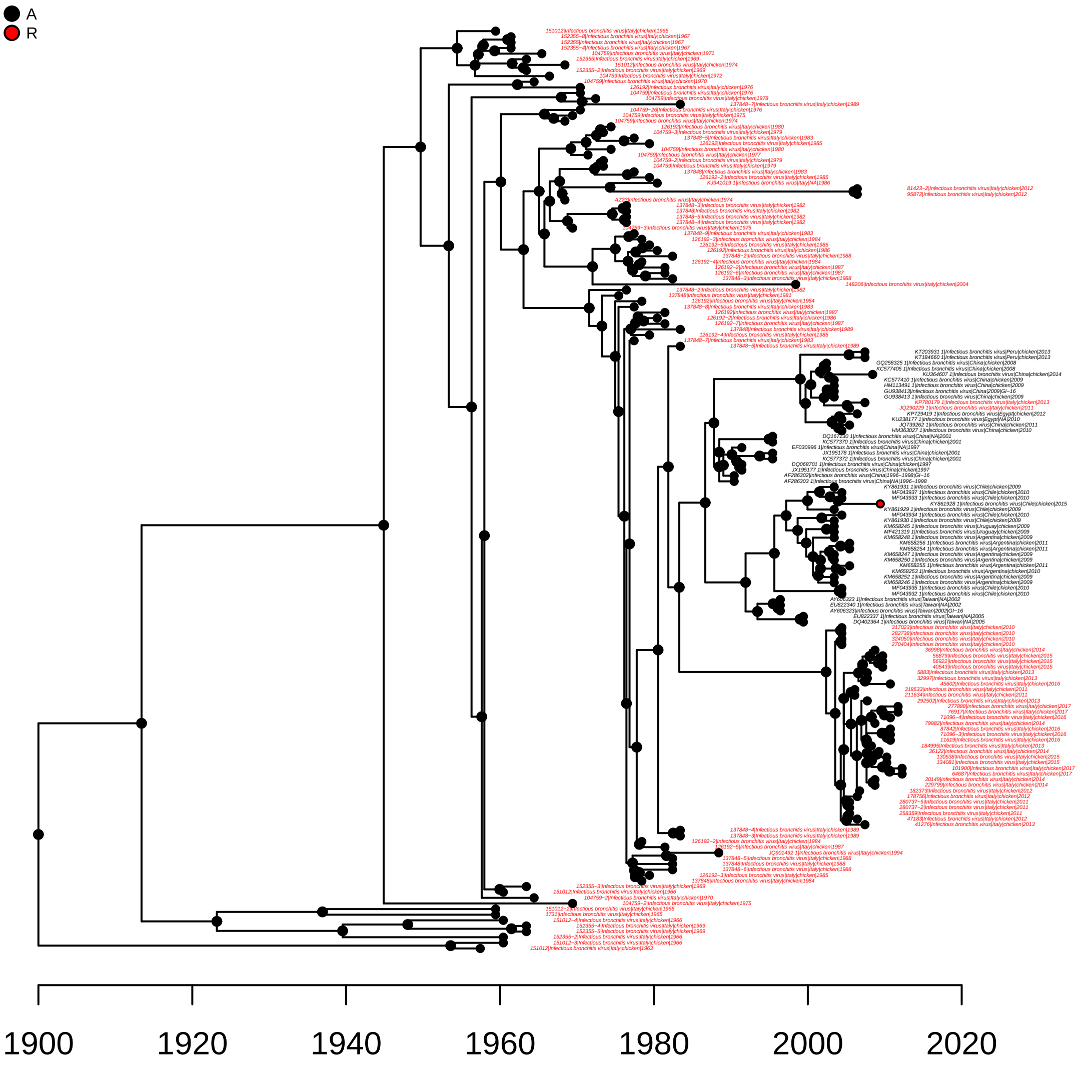

47

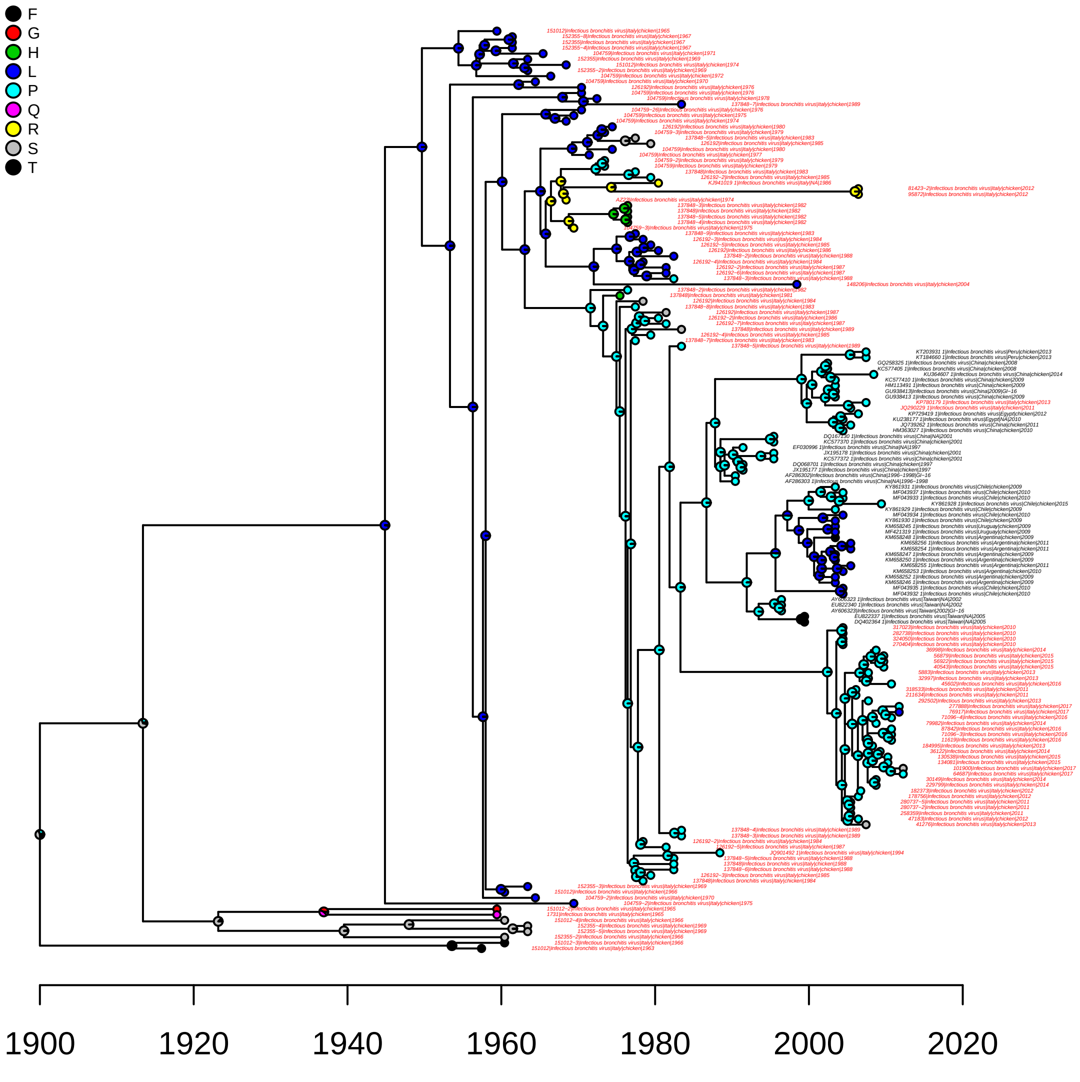

48

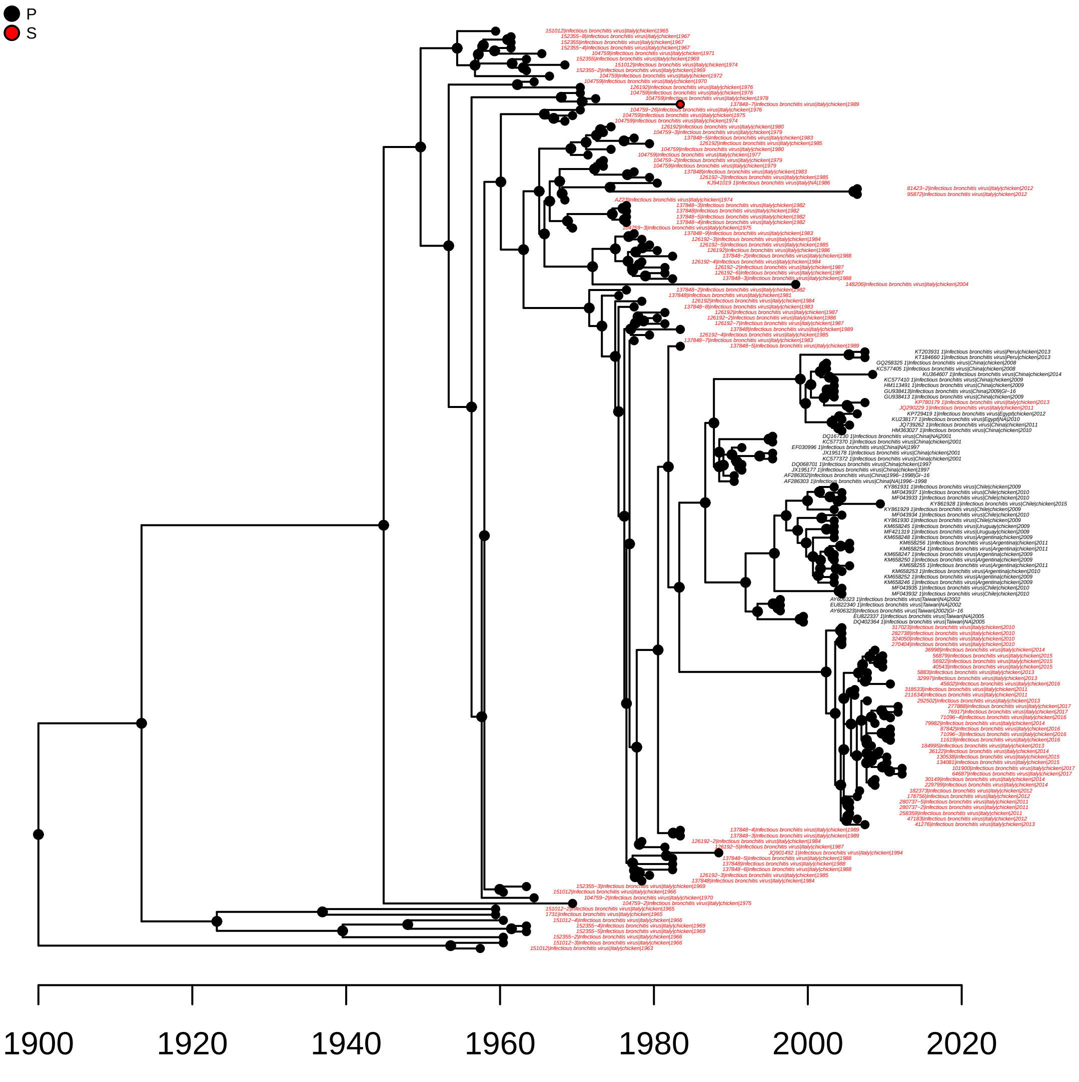

49

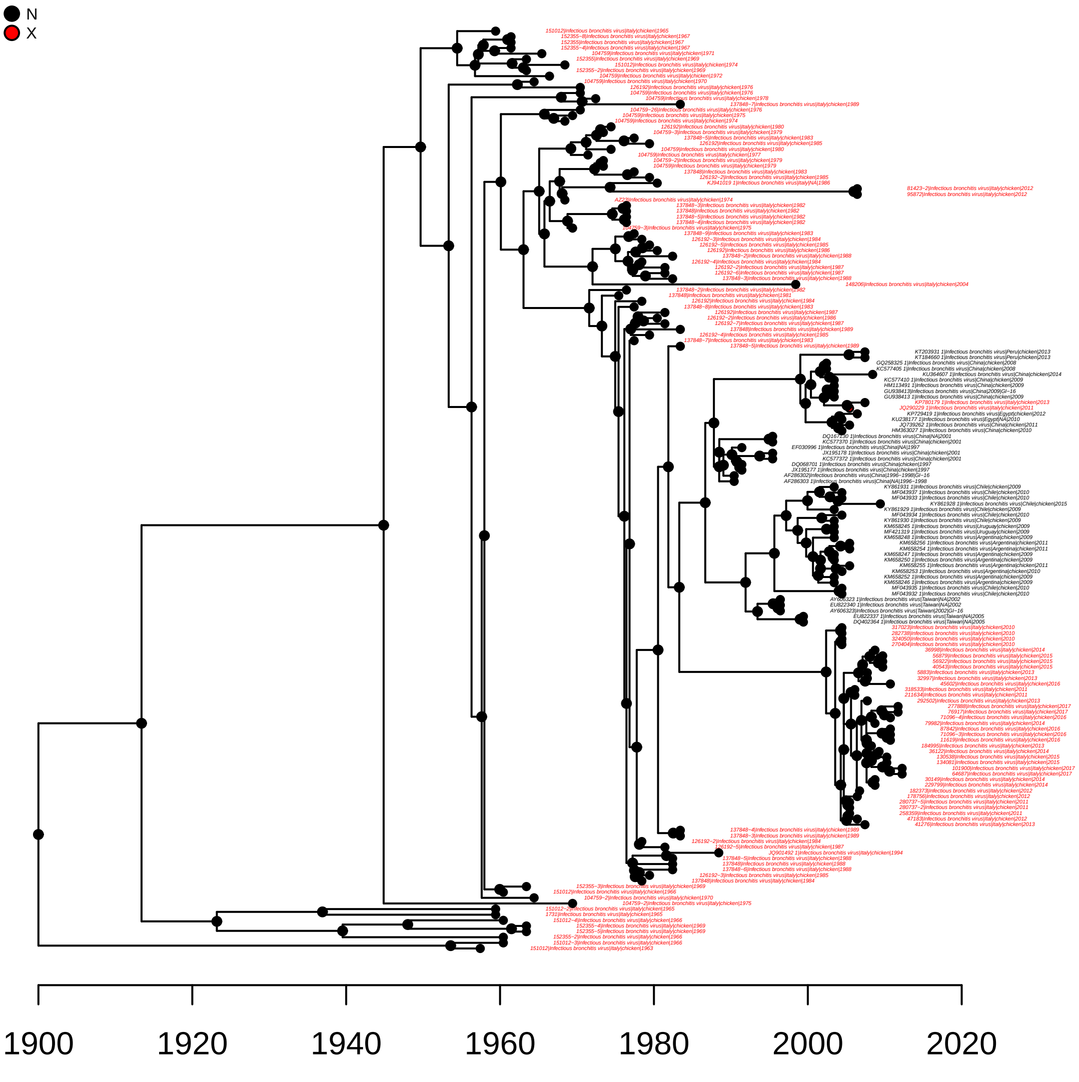

50

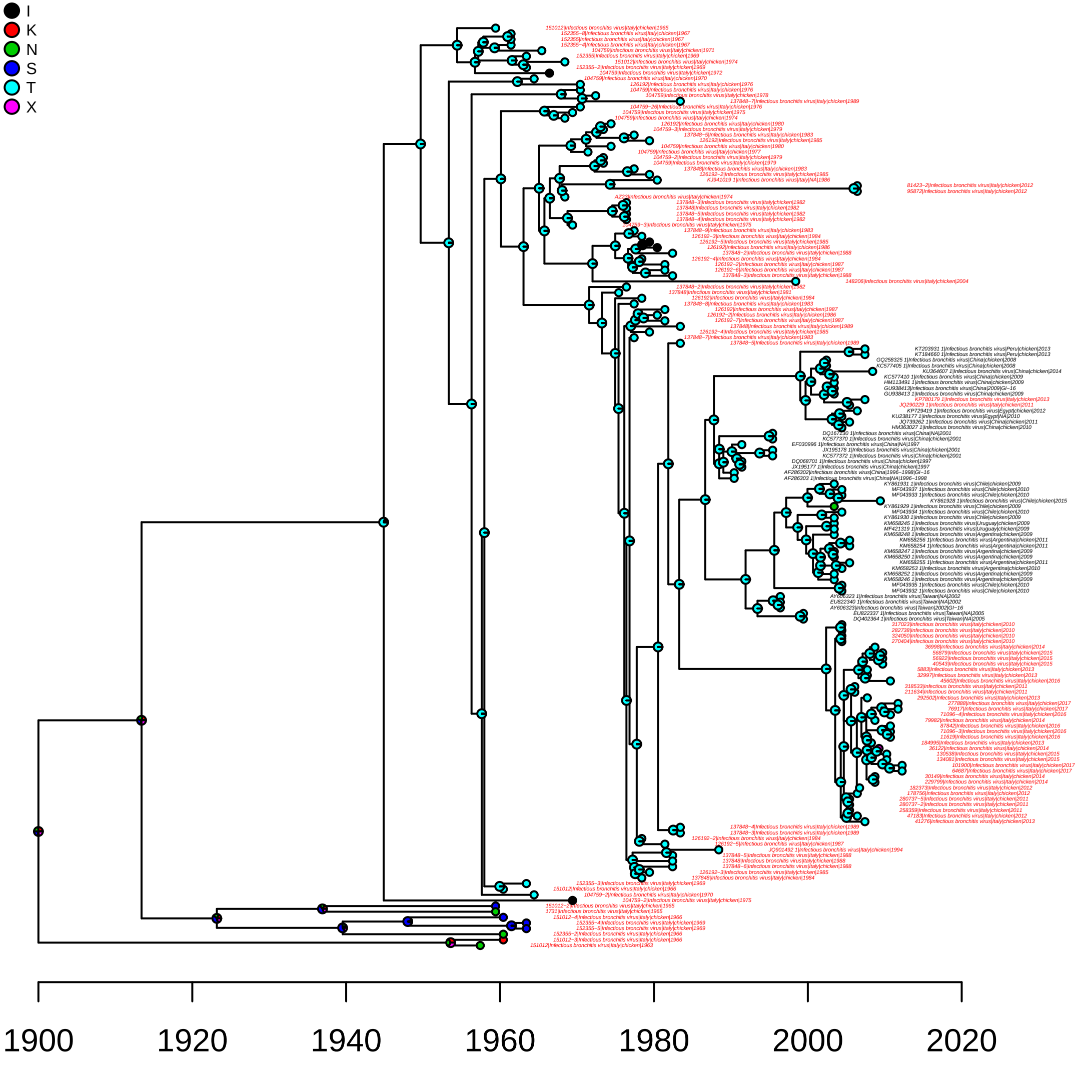

51

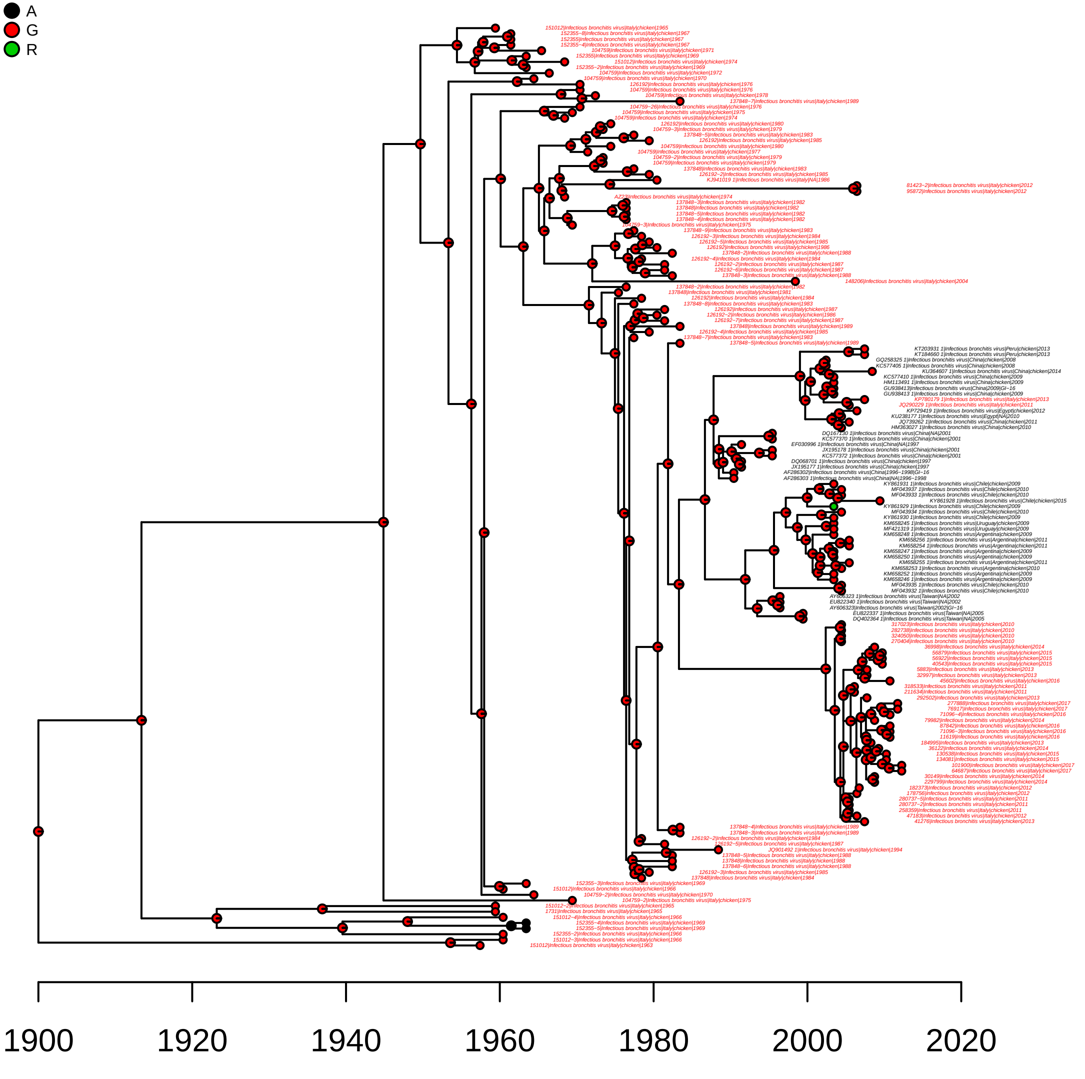

52

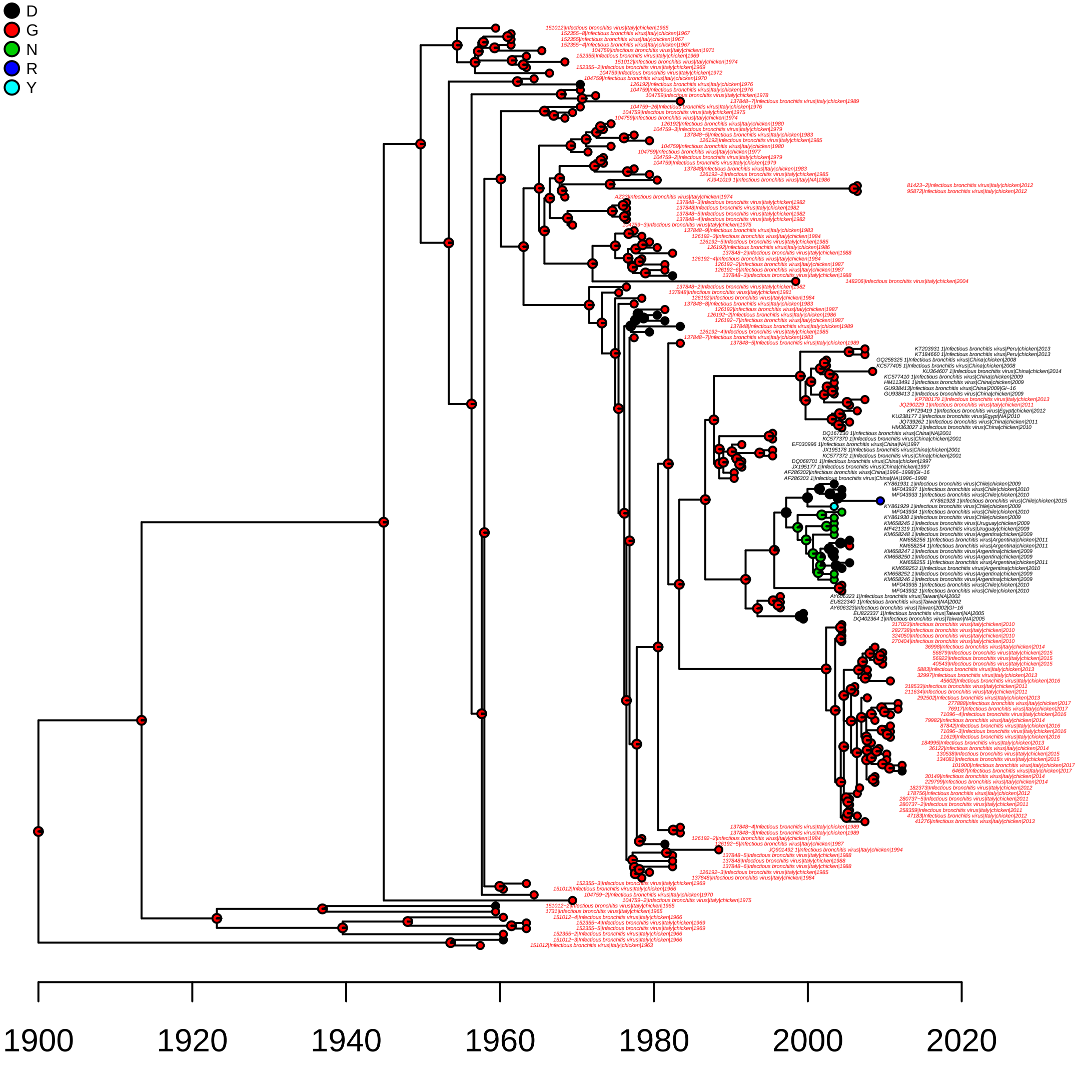

53

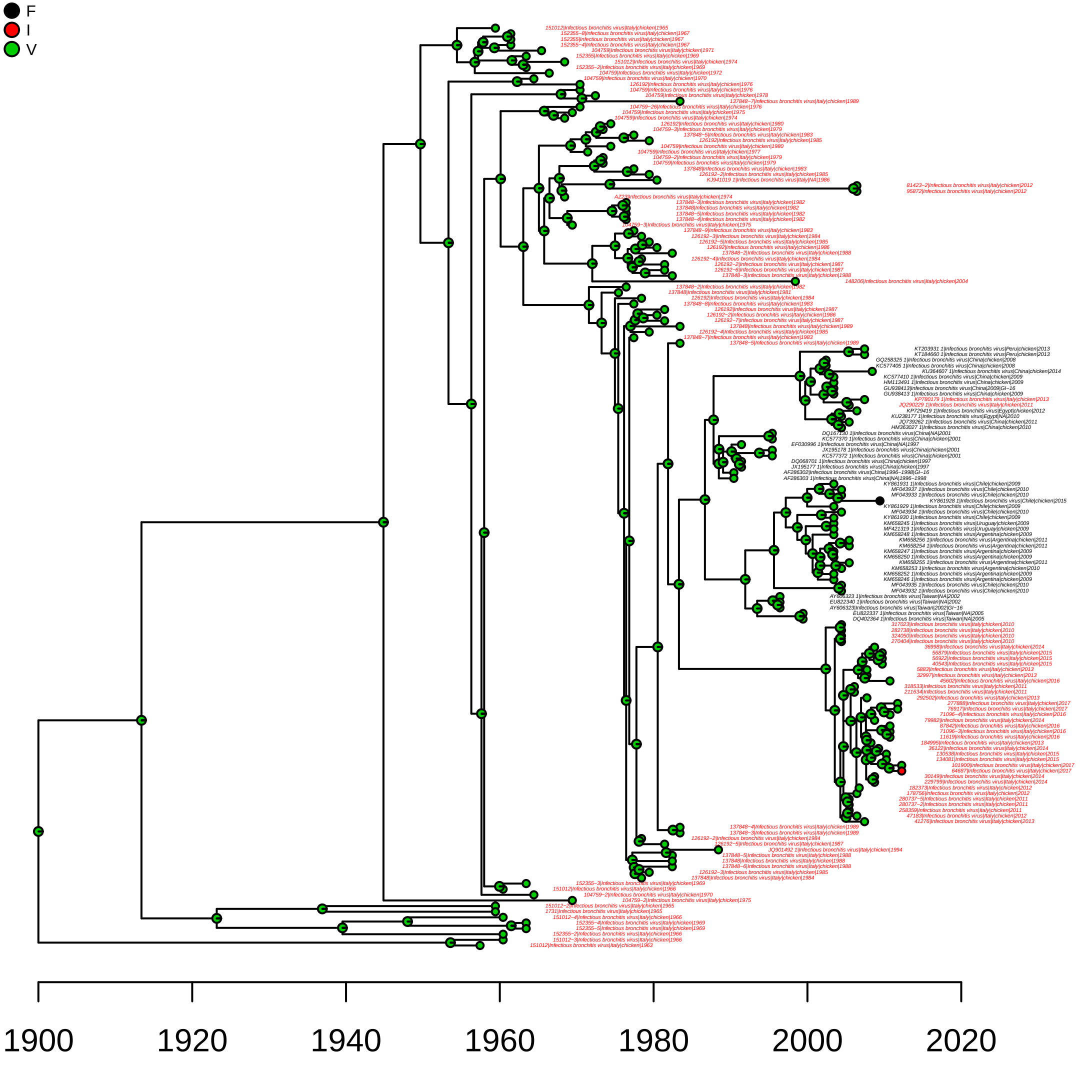

54

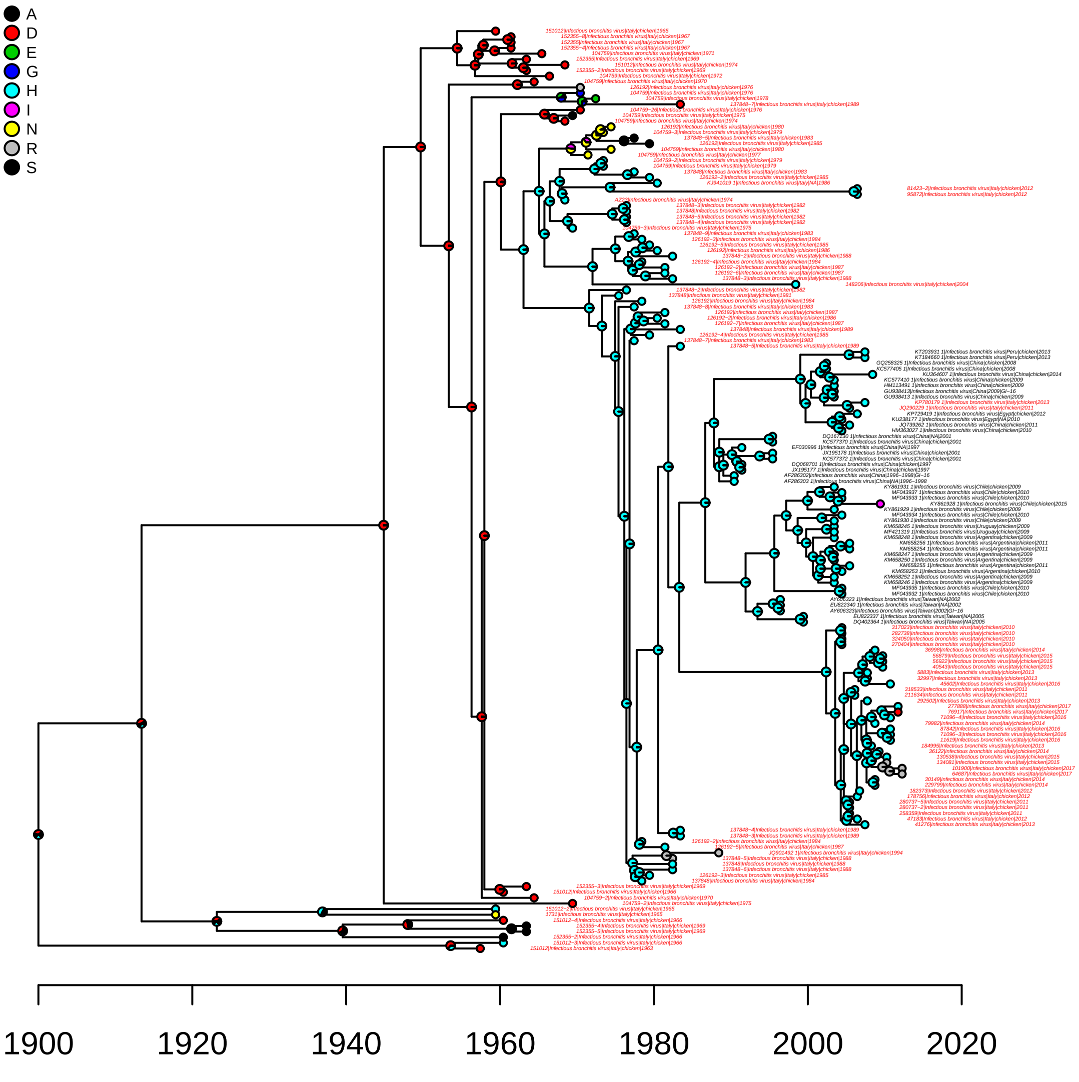

55

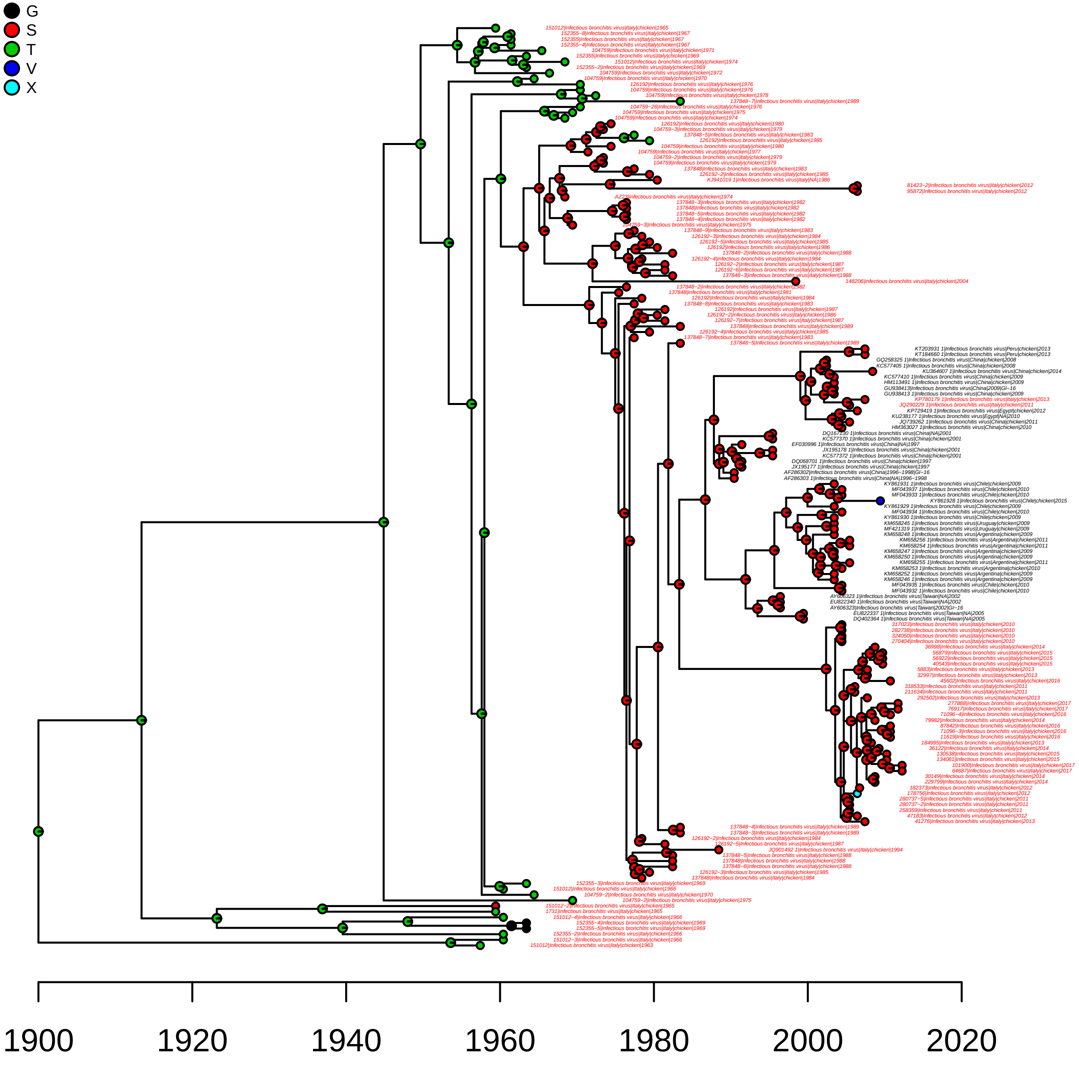

56

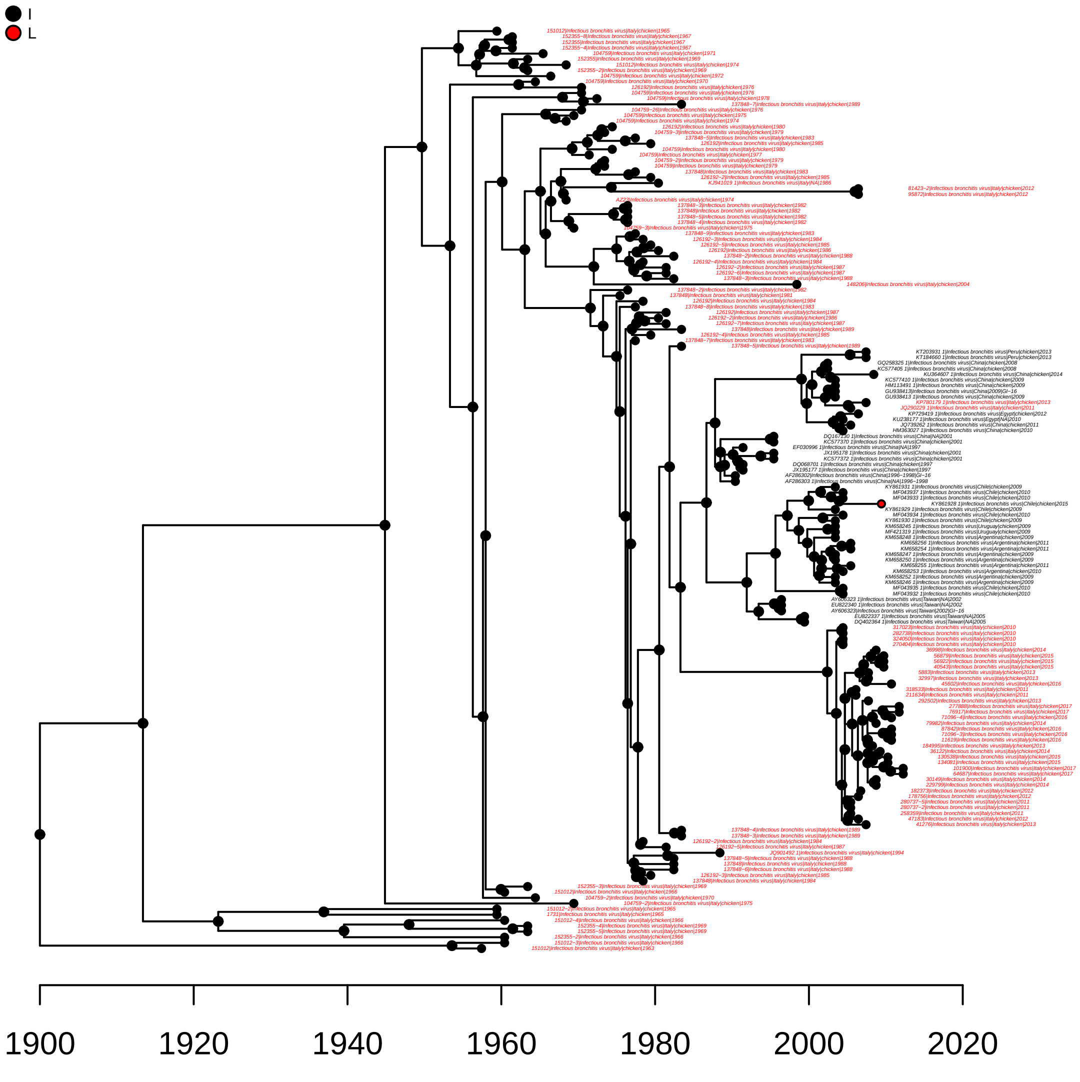

57

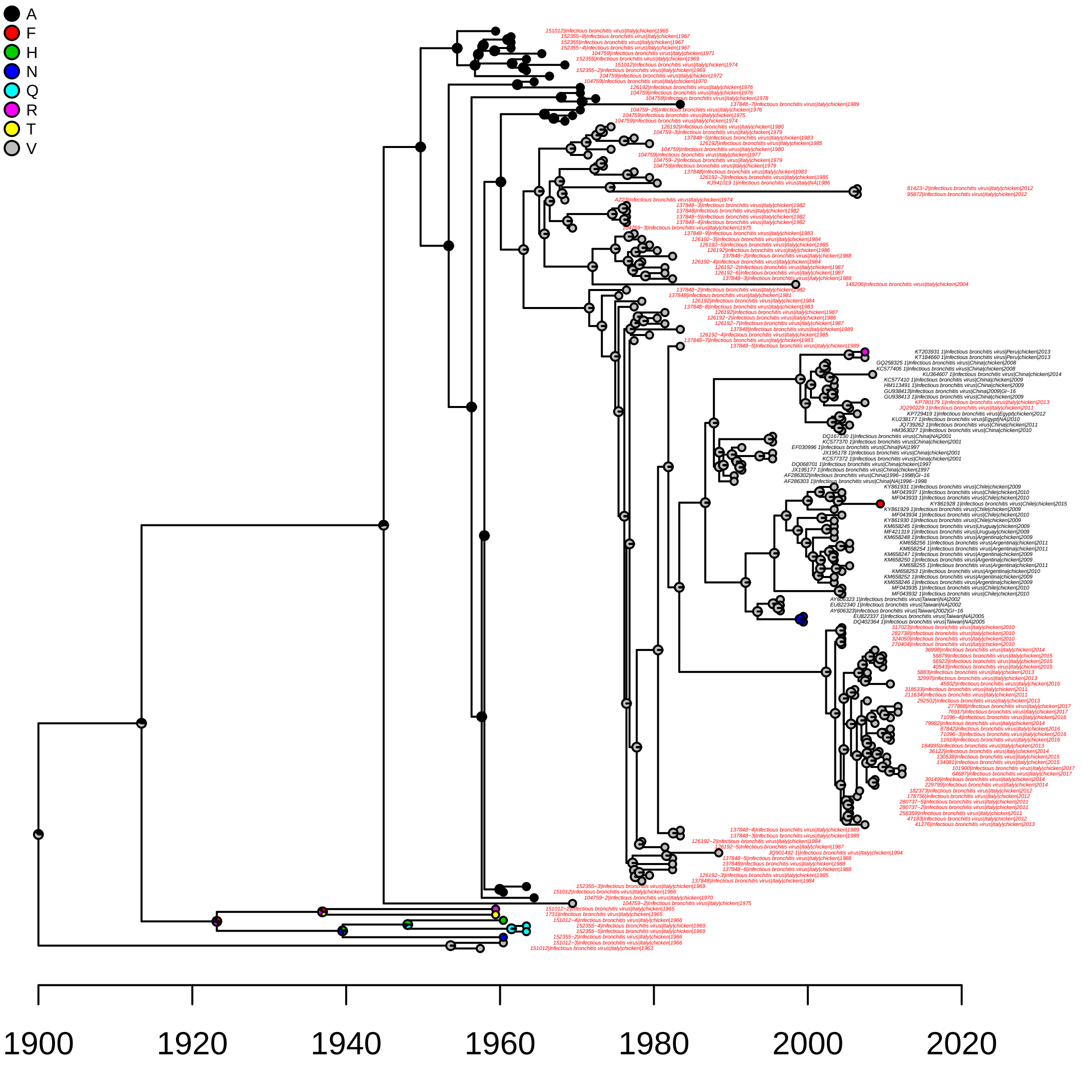

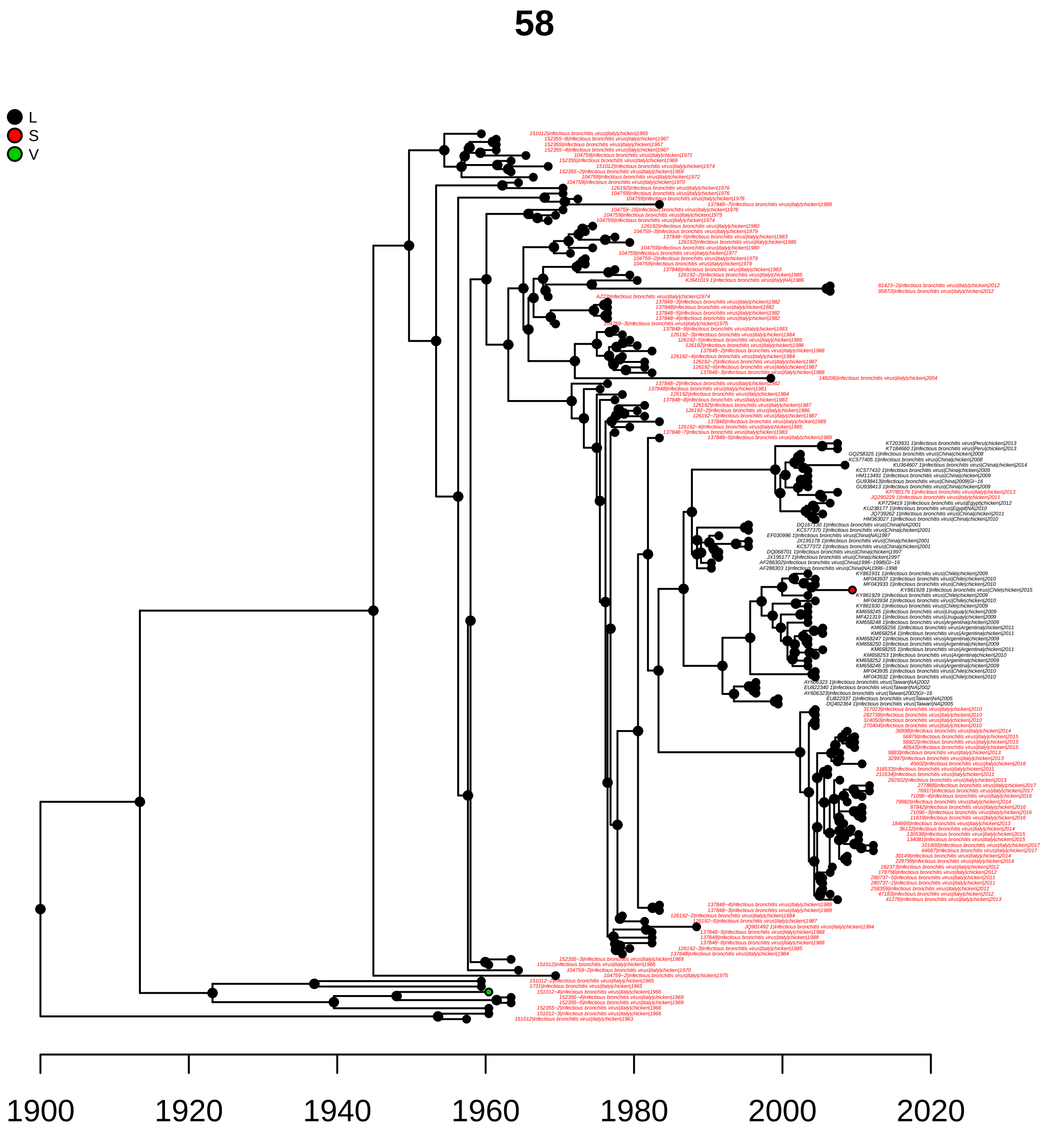
58
L
S
V
1900
1920
1940
1960
1980
2000
2020

59

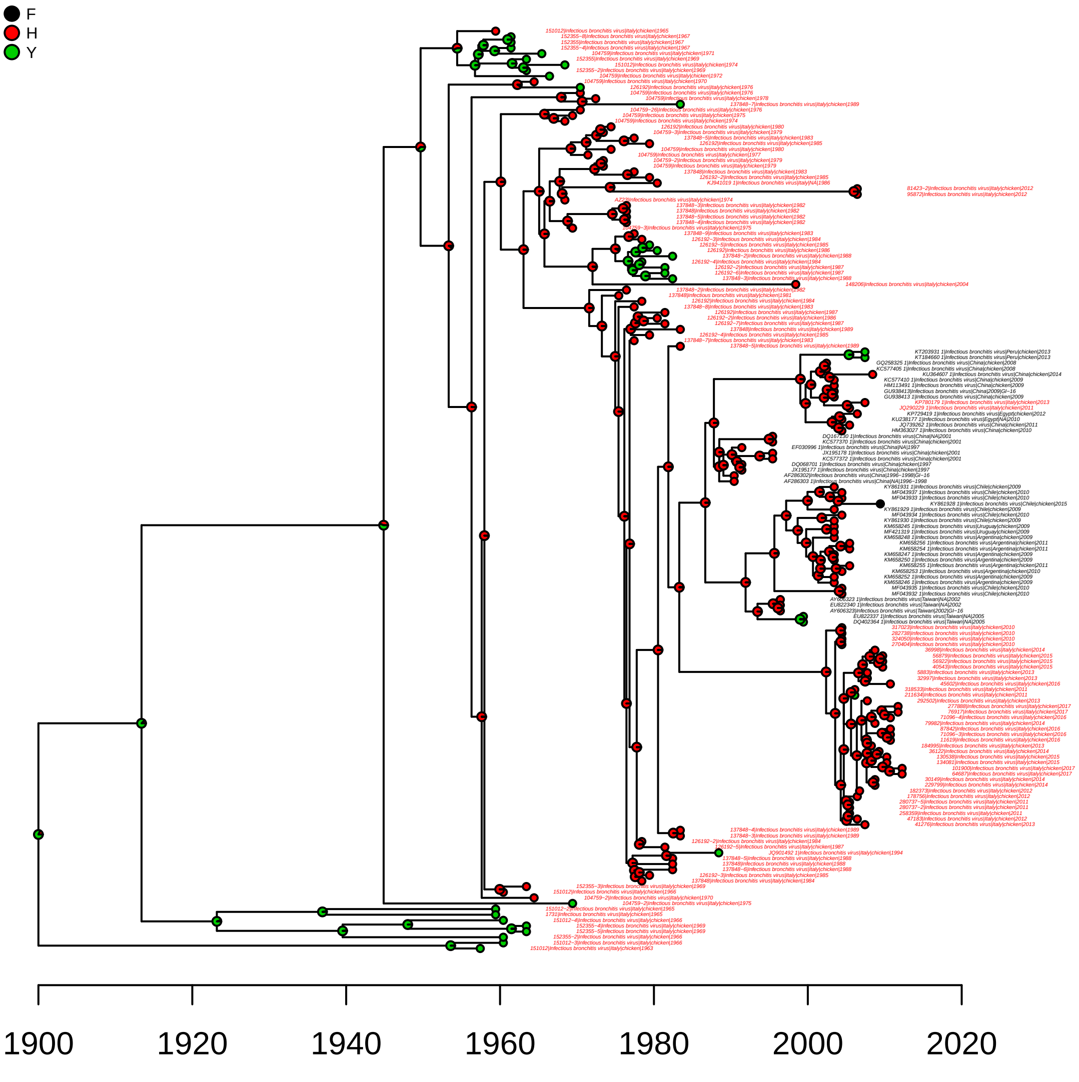

60

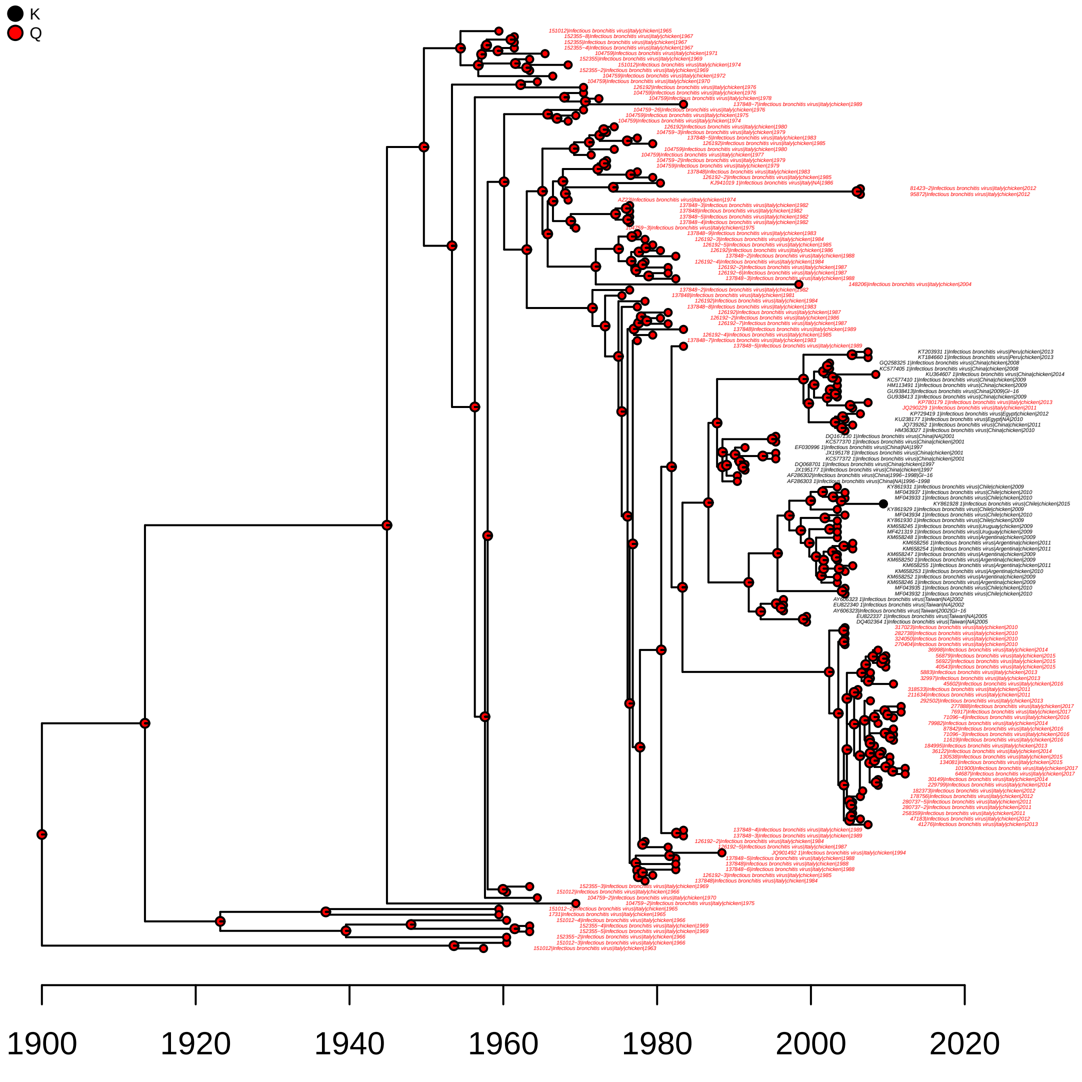

61

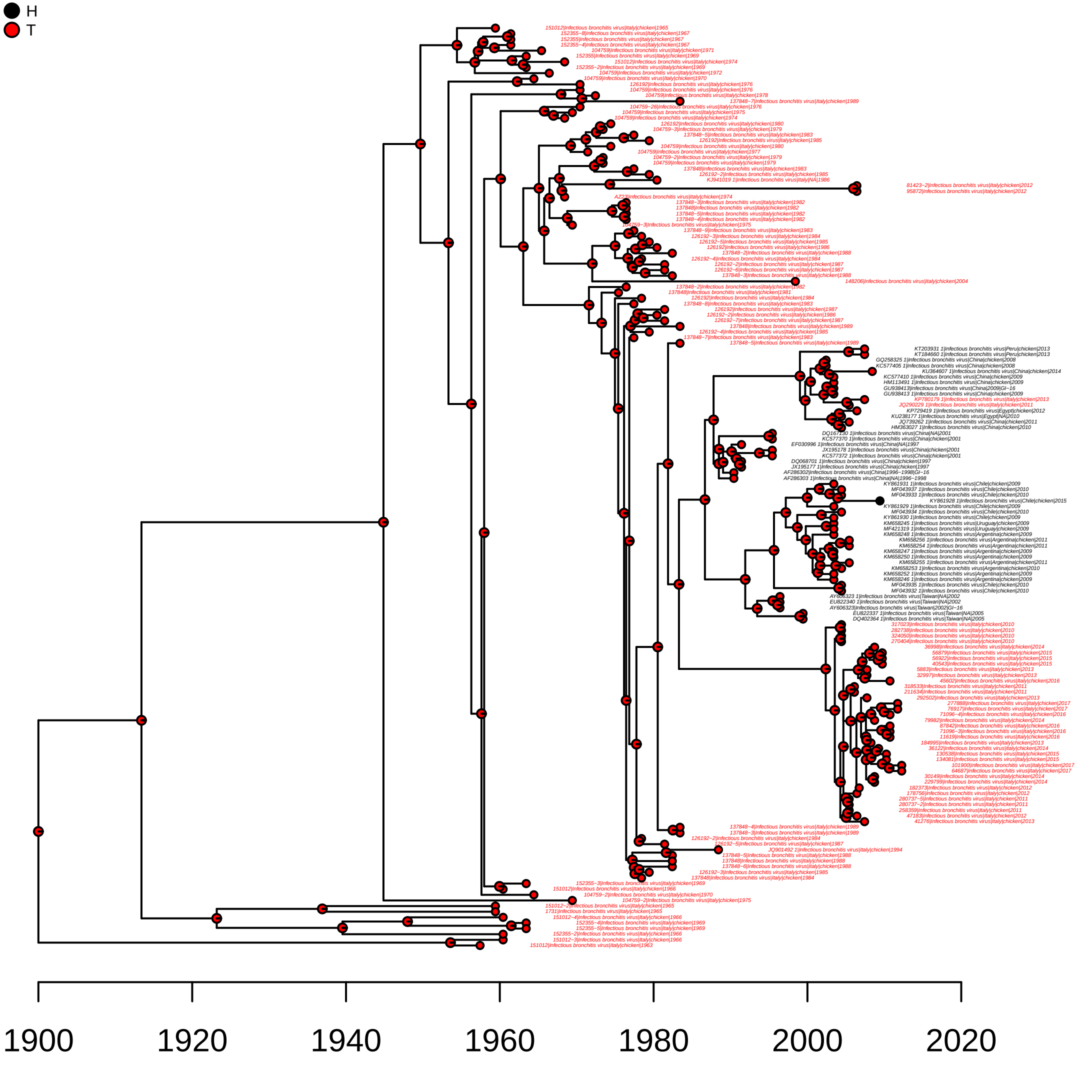

62

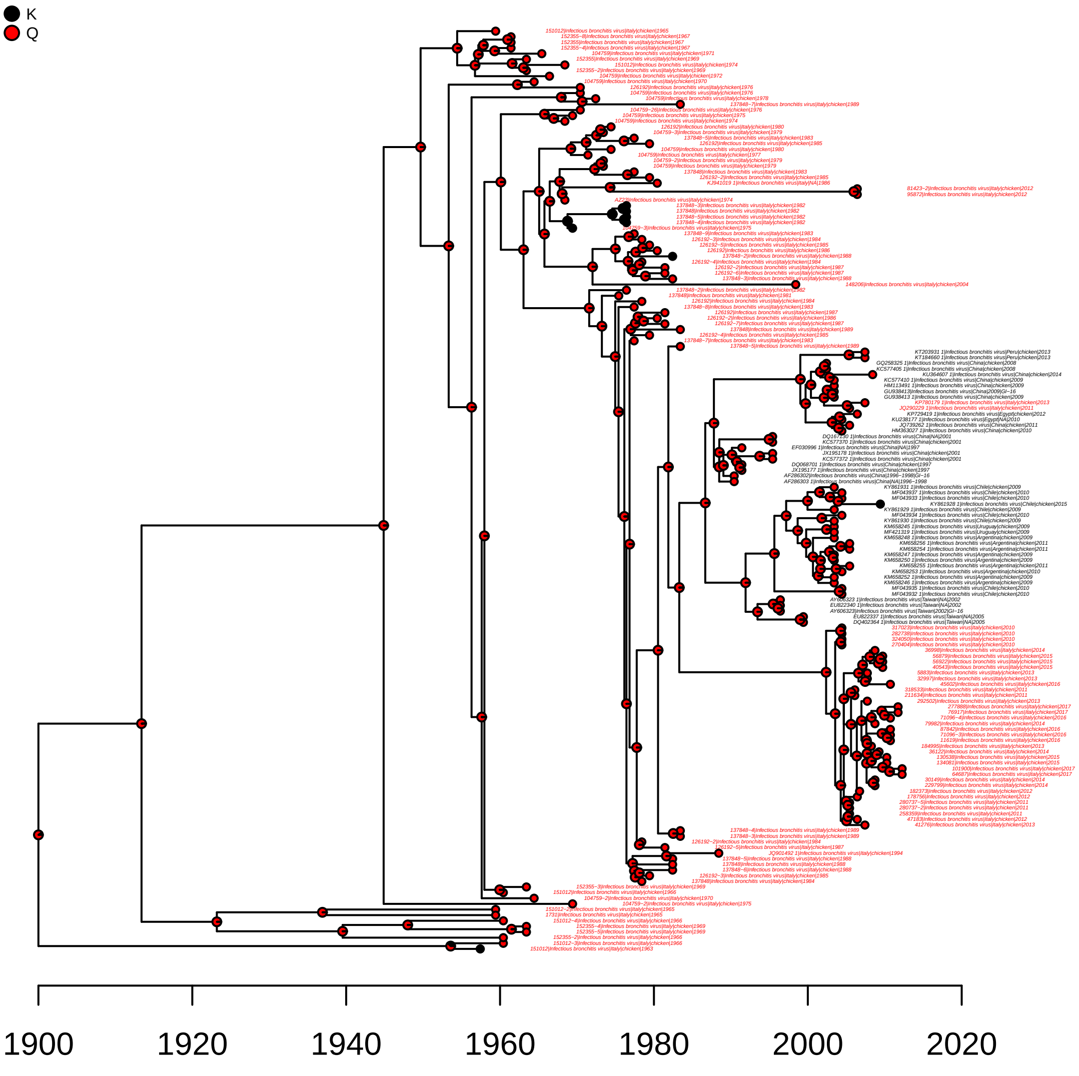

63

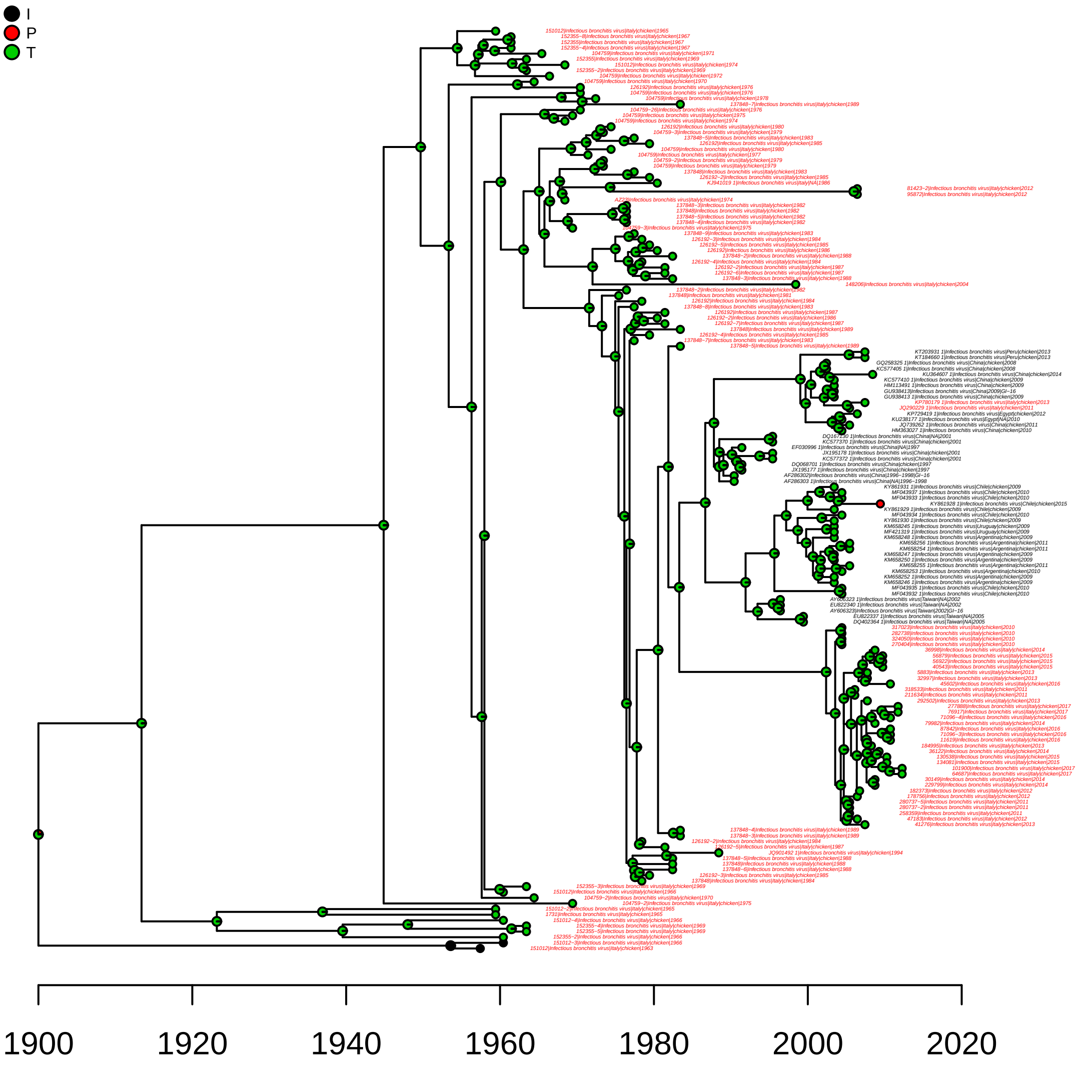

64

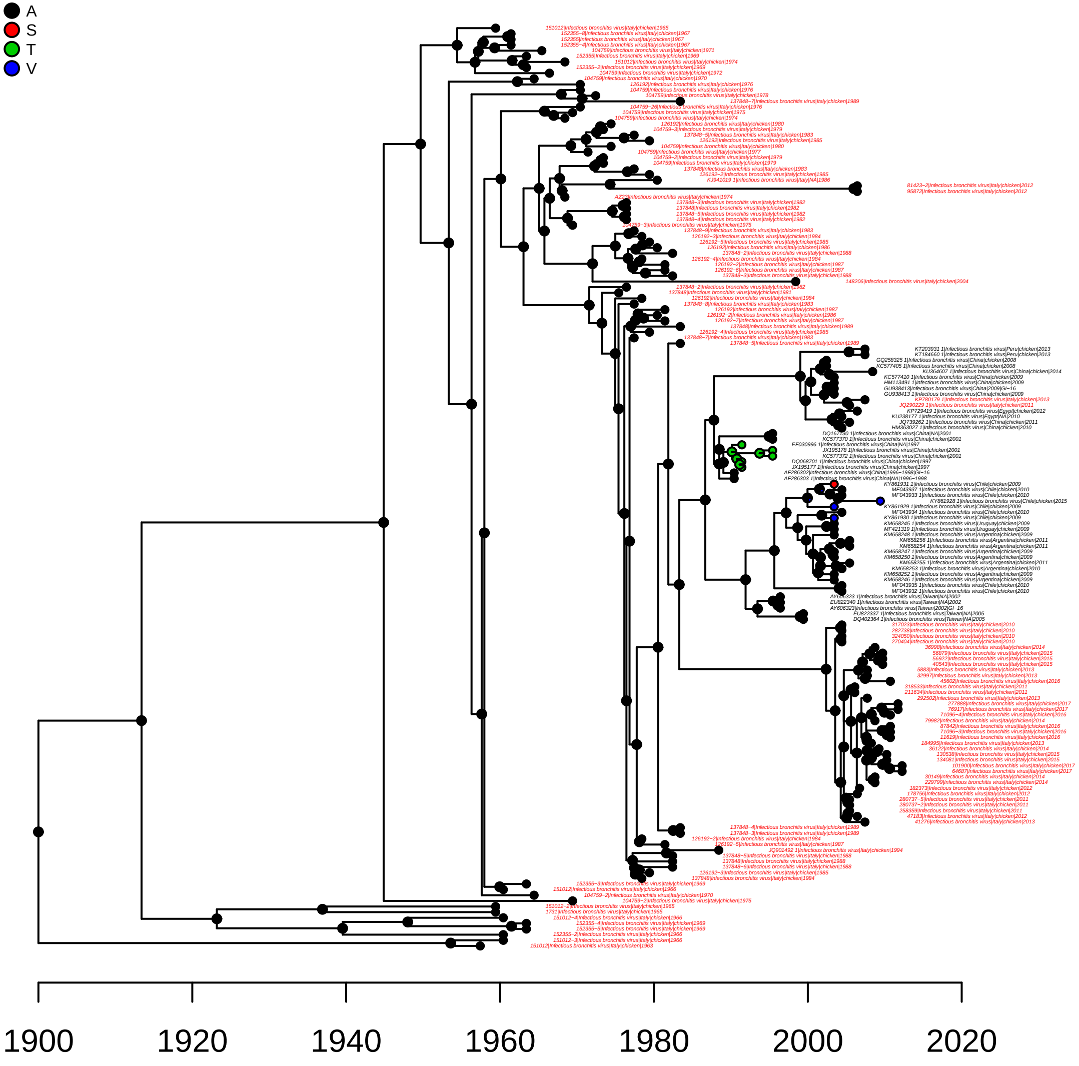

65

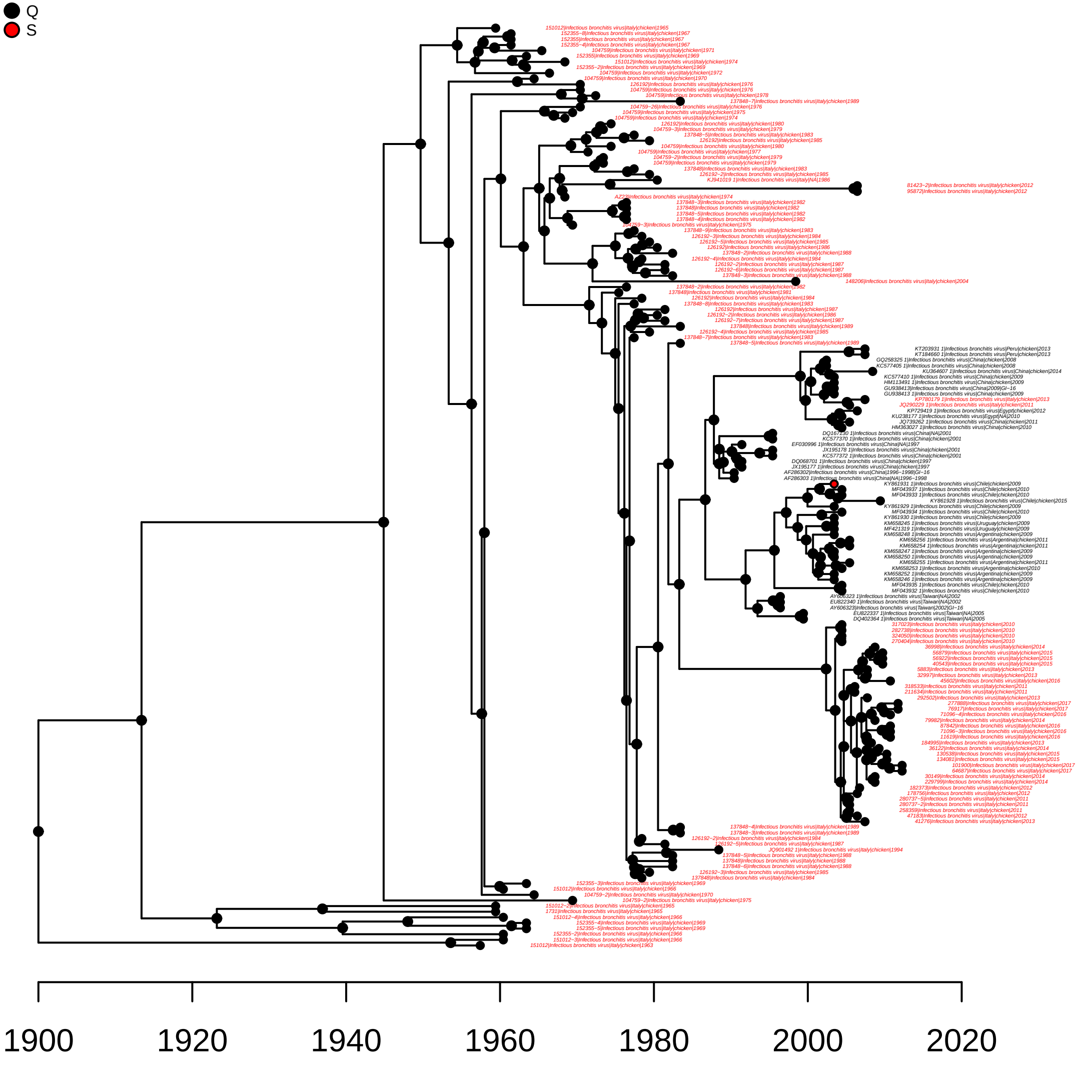

68

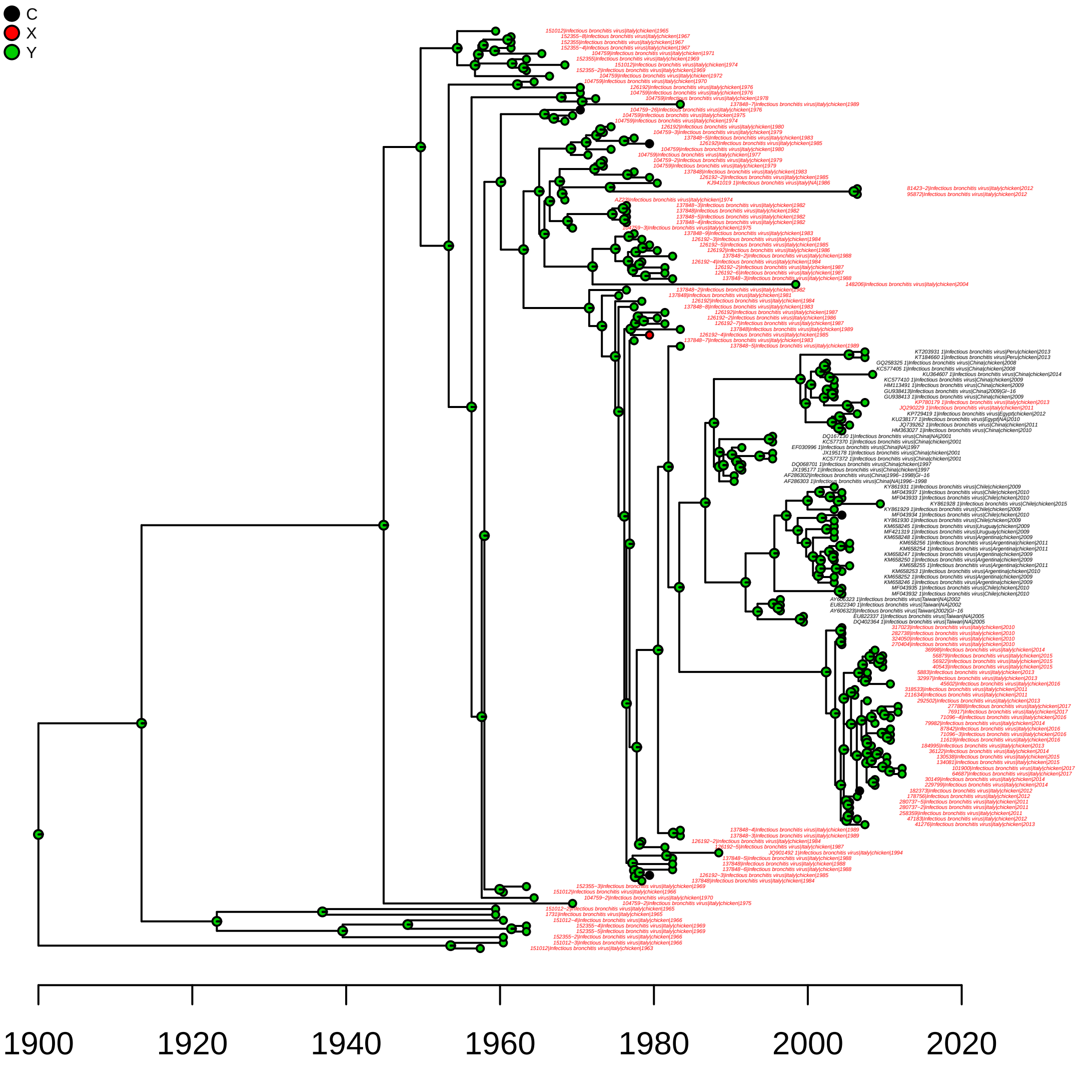

69

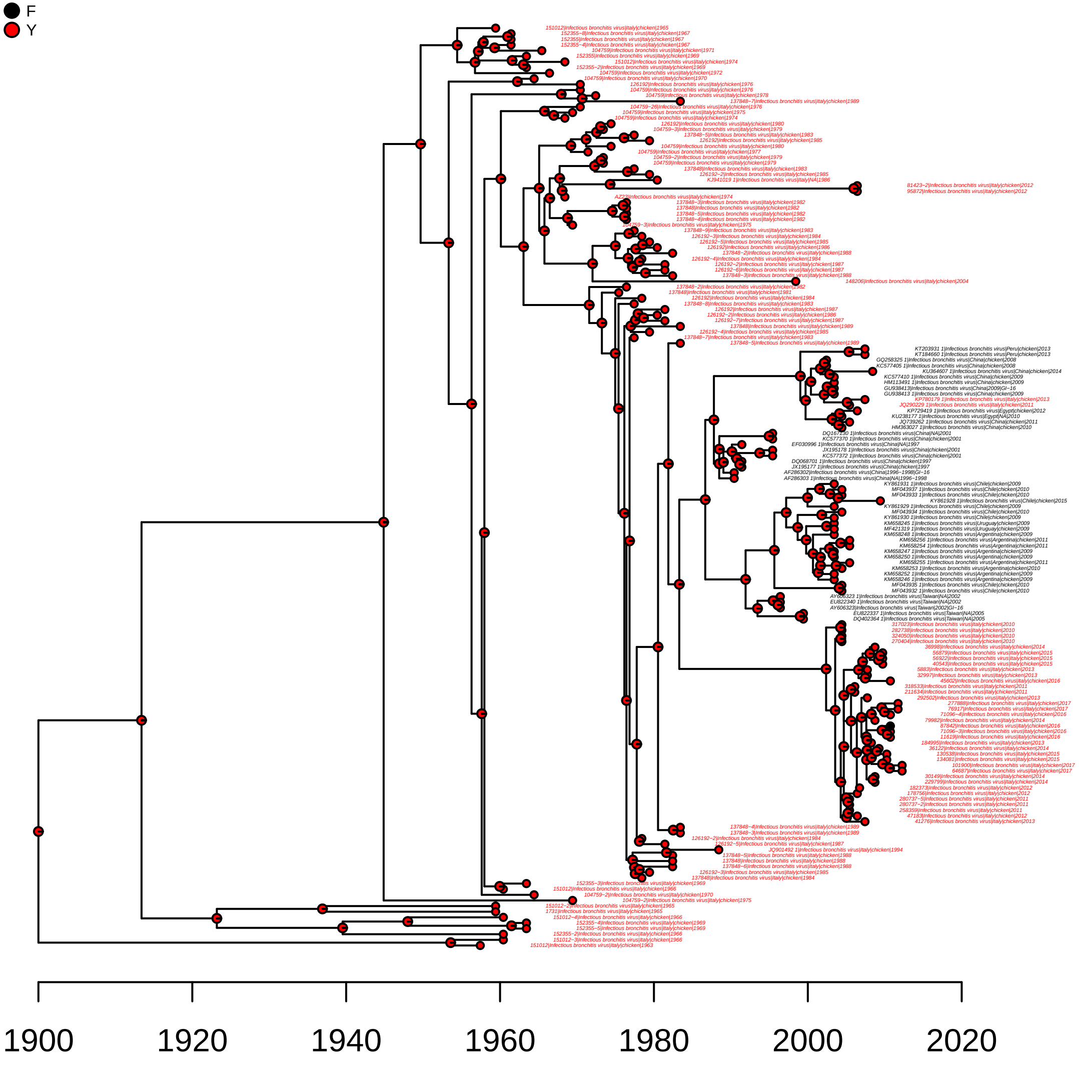

71

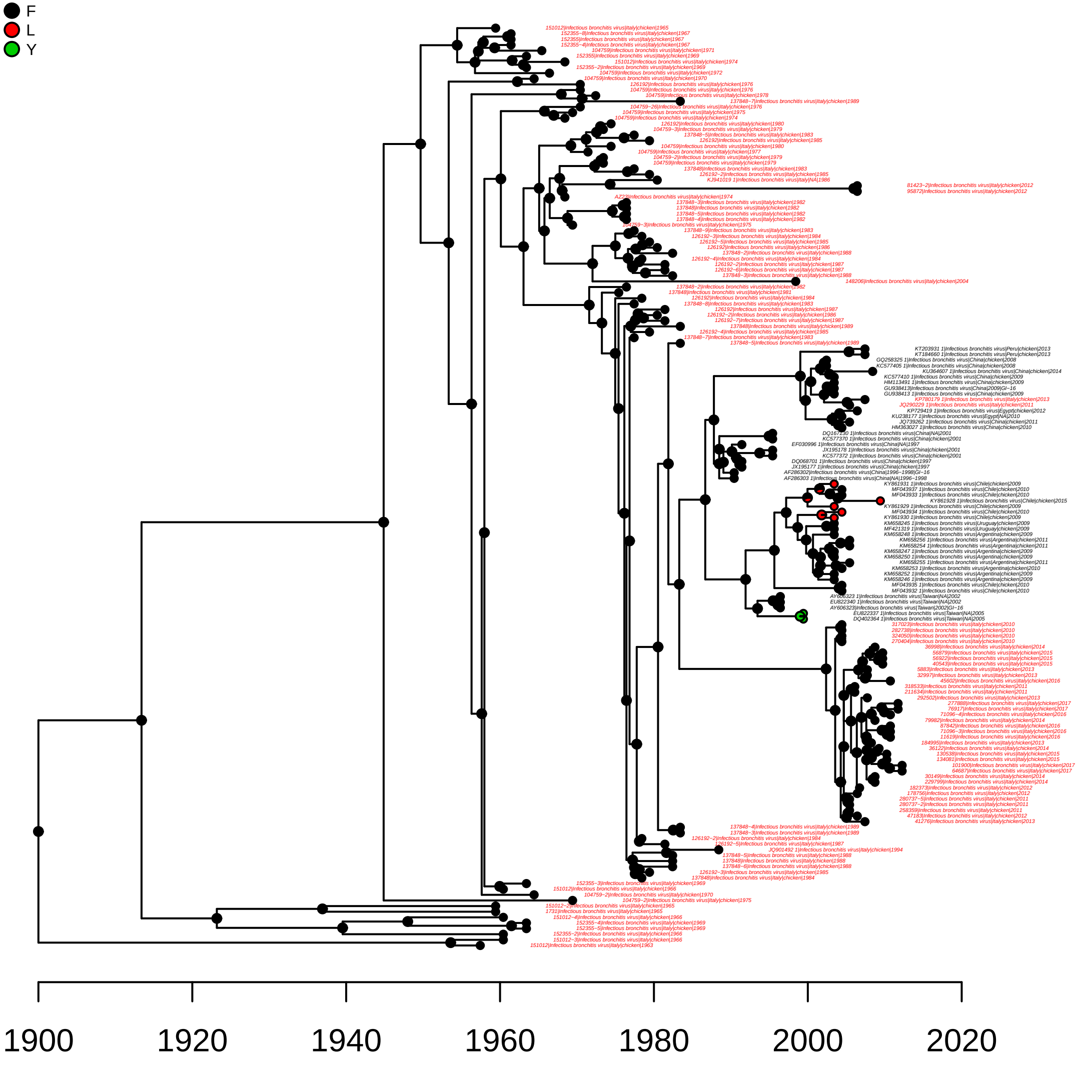

73

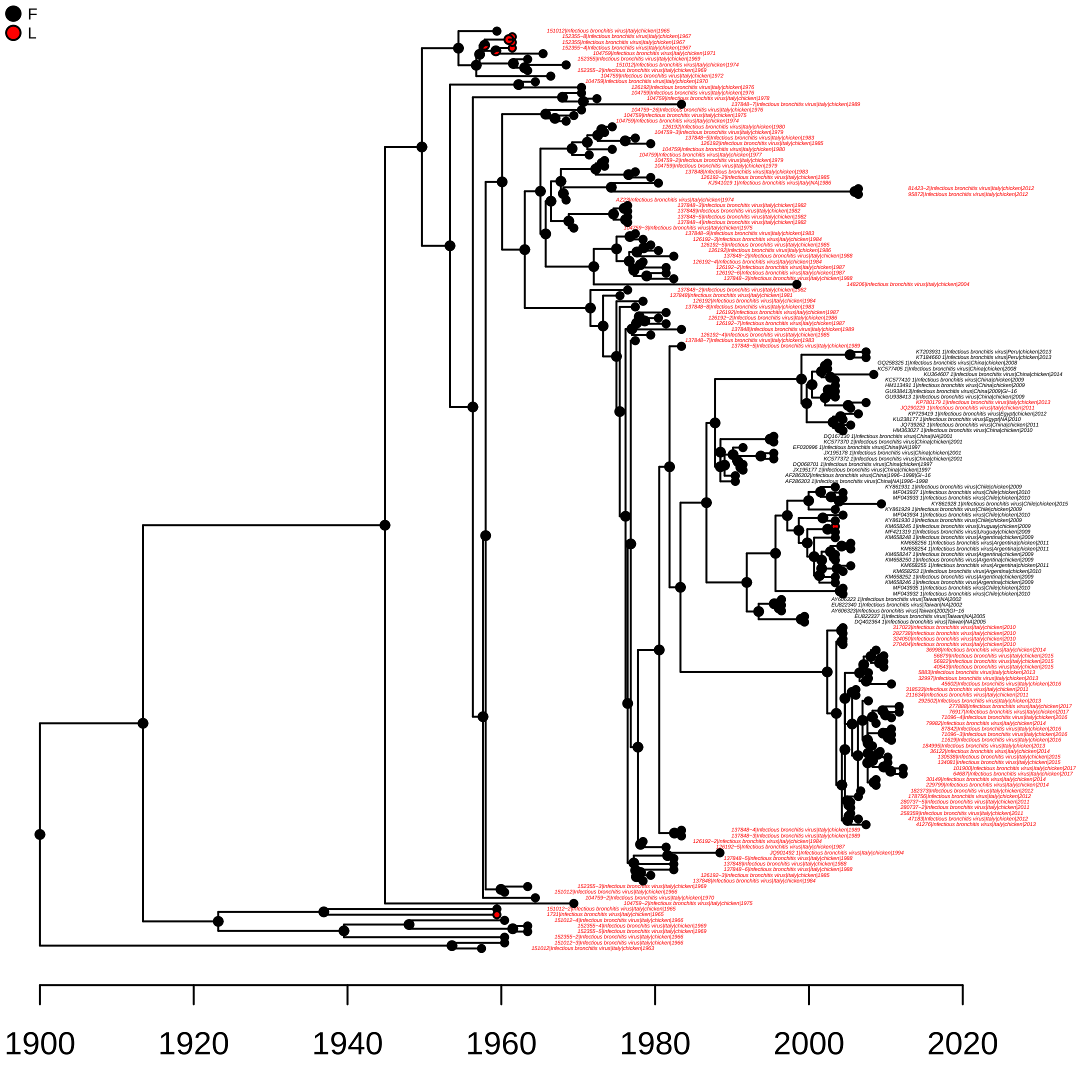

77

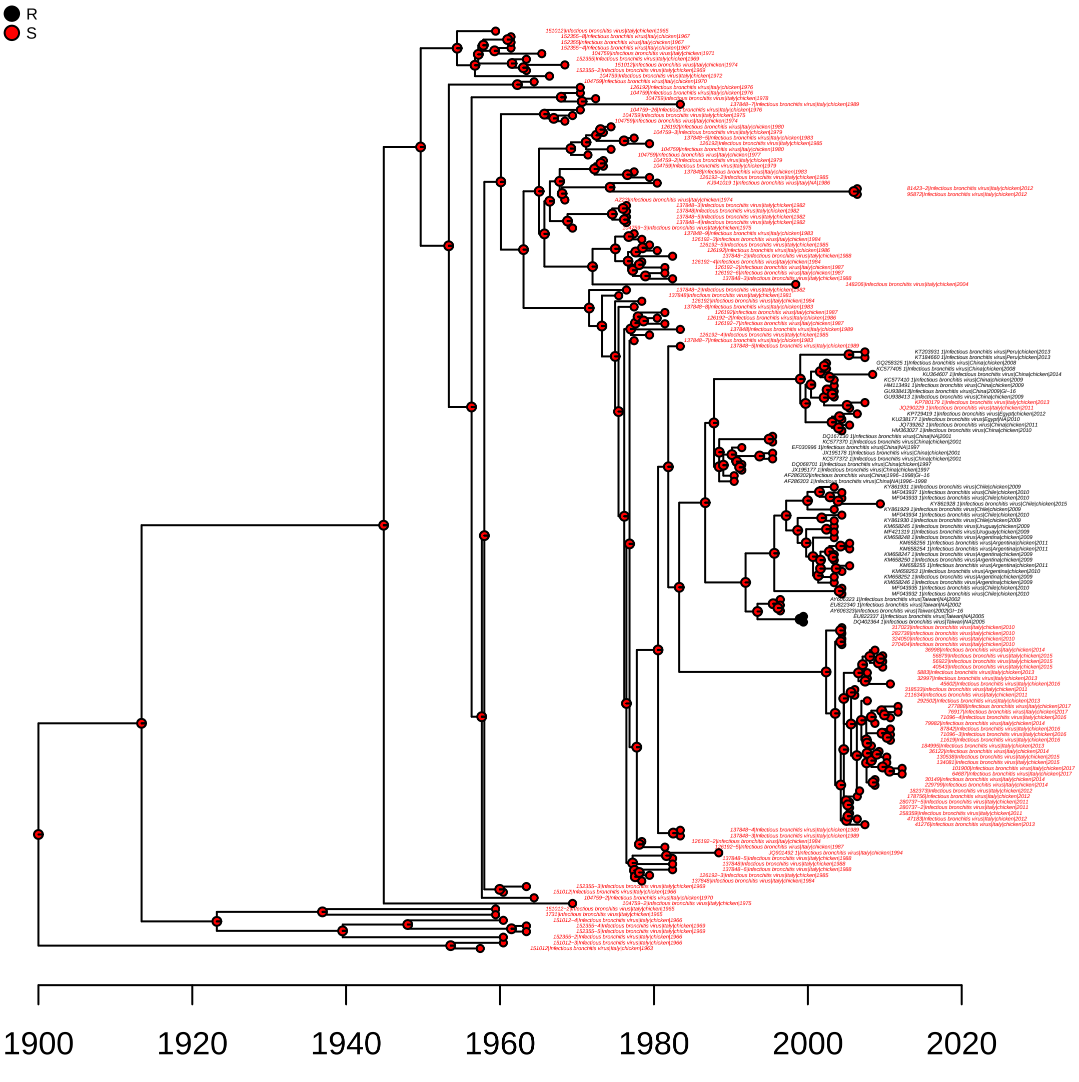

78

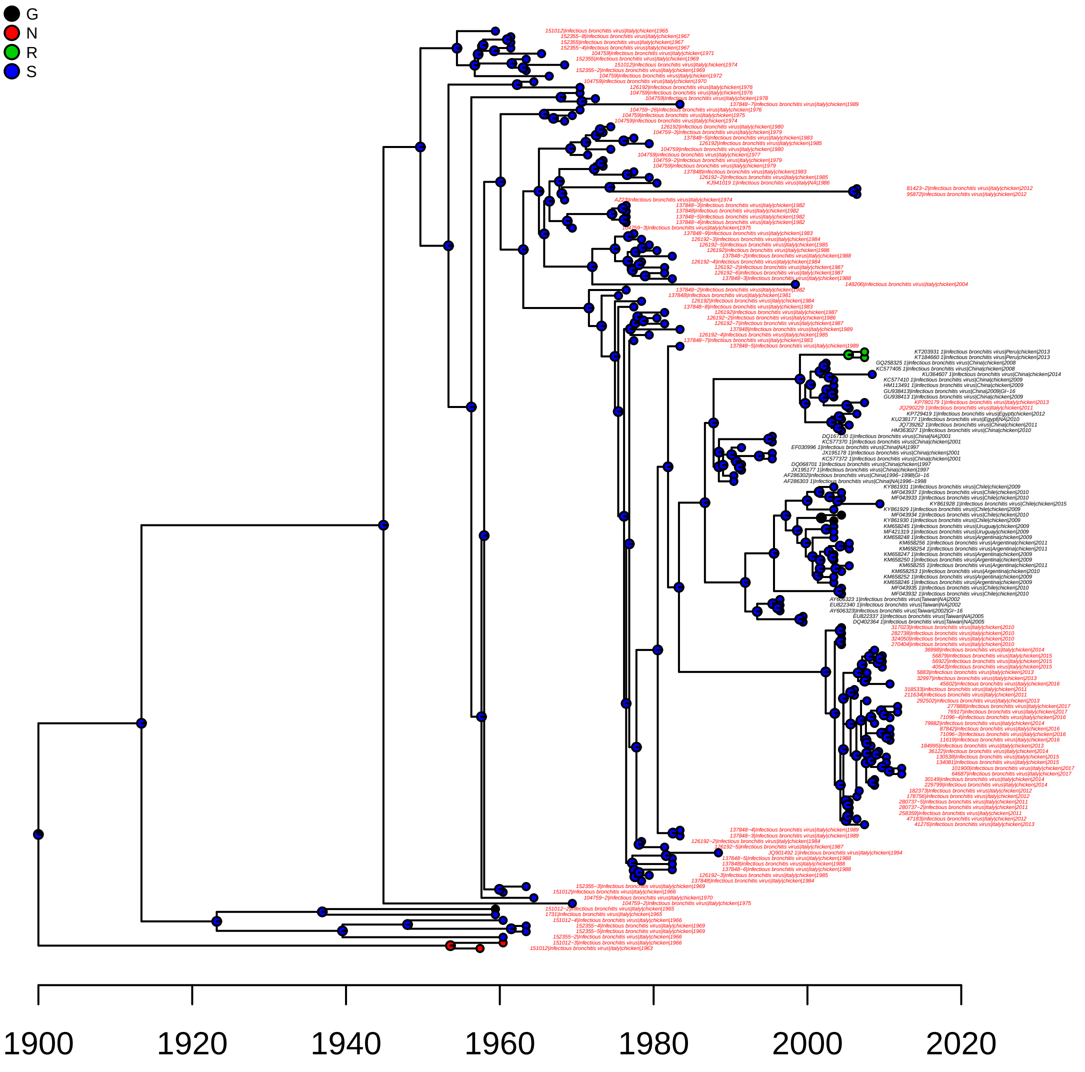

80

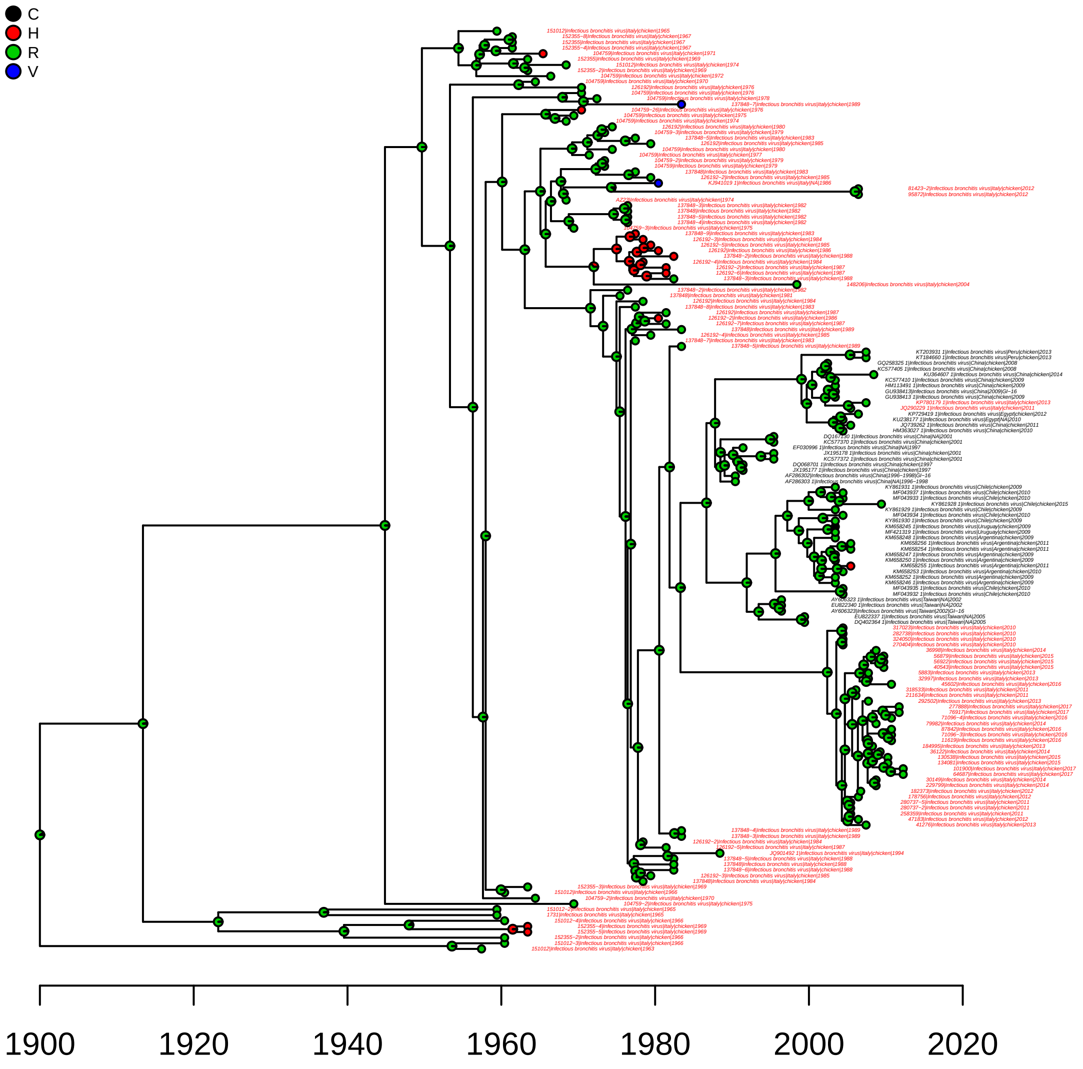

82

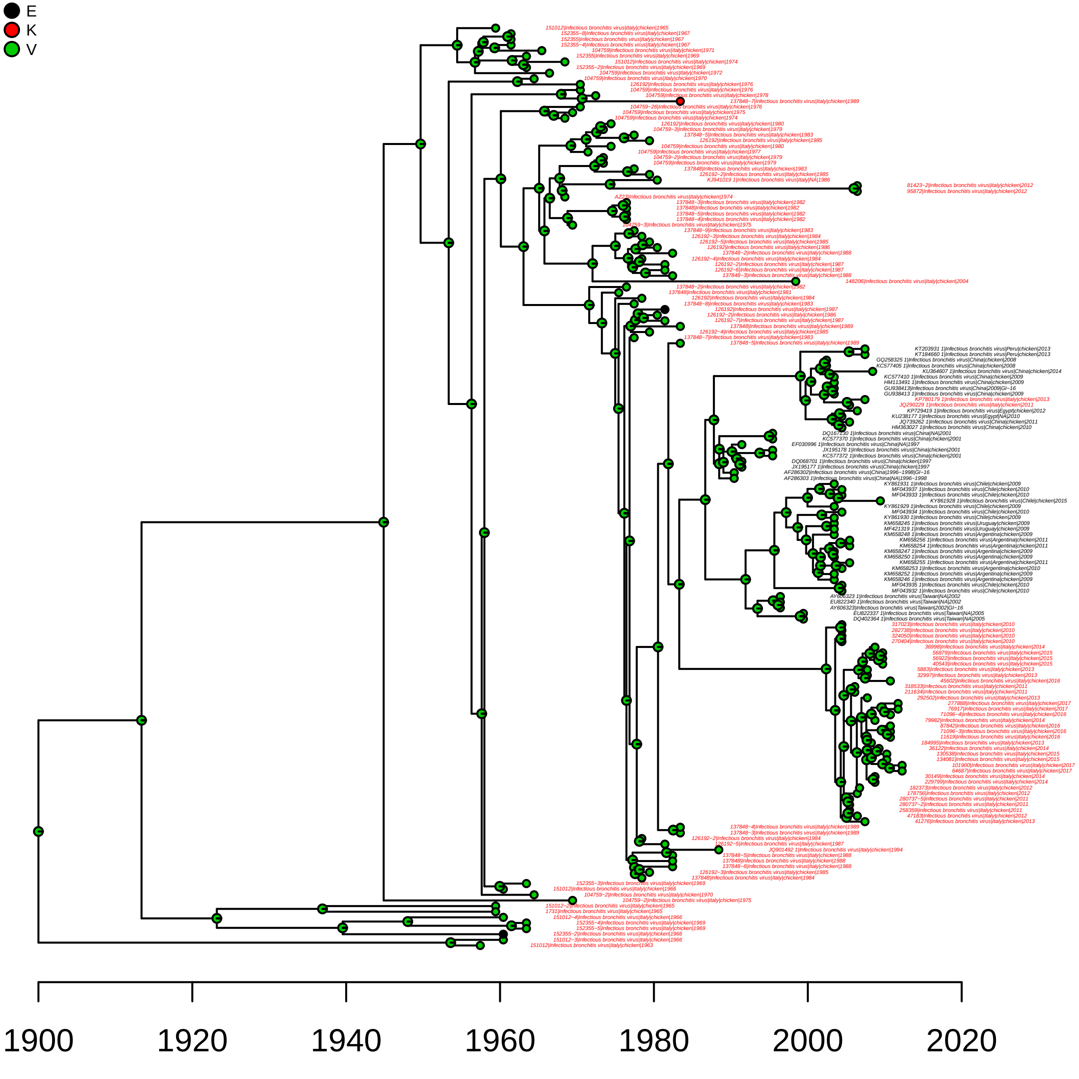

83

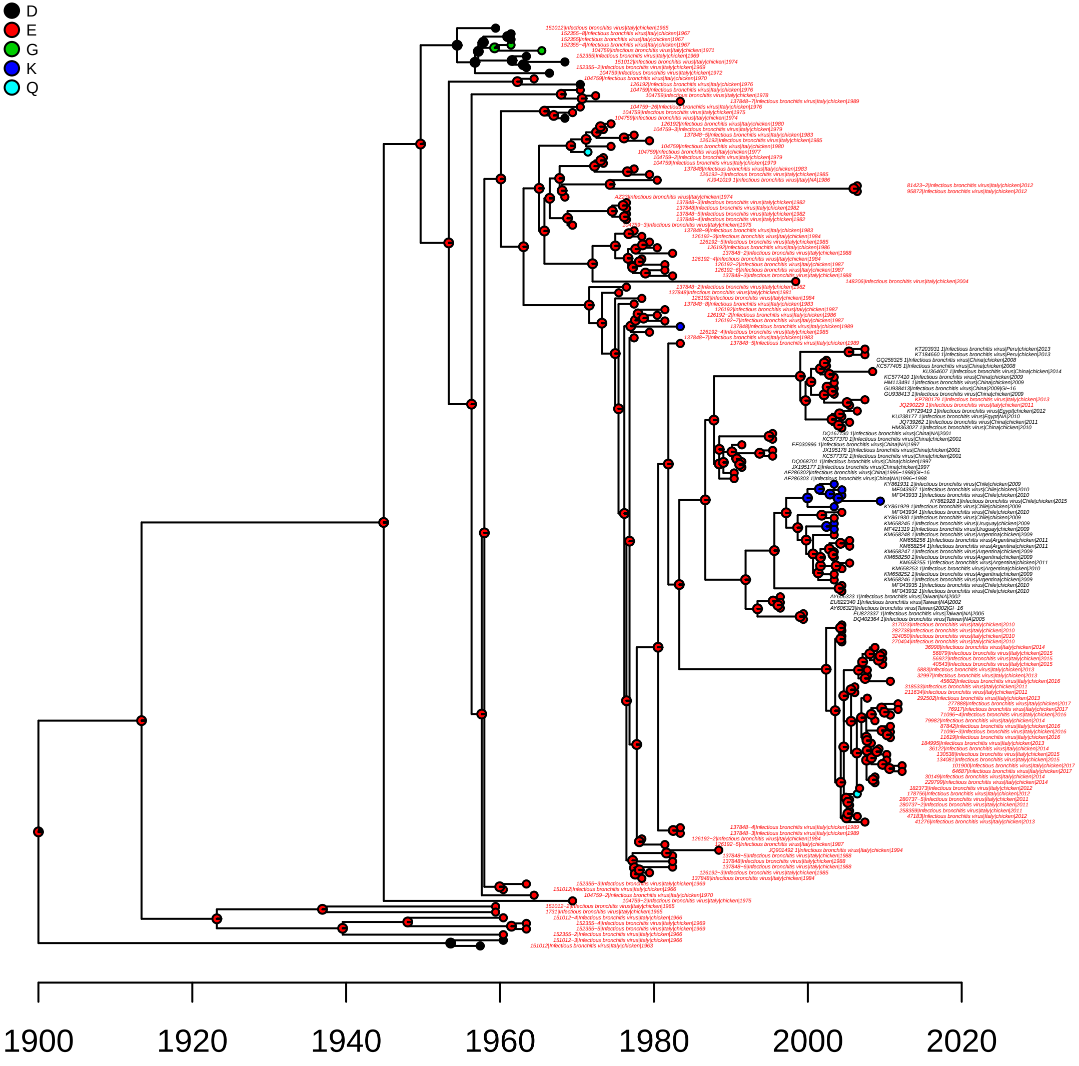

85

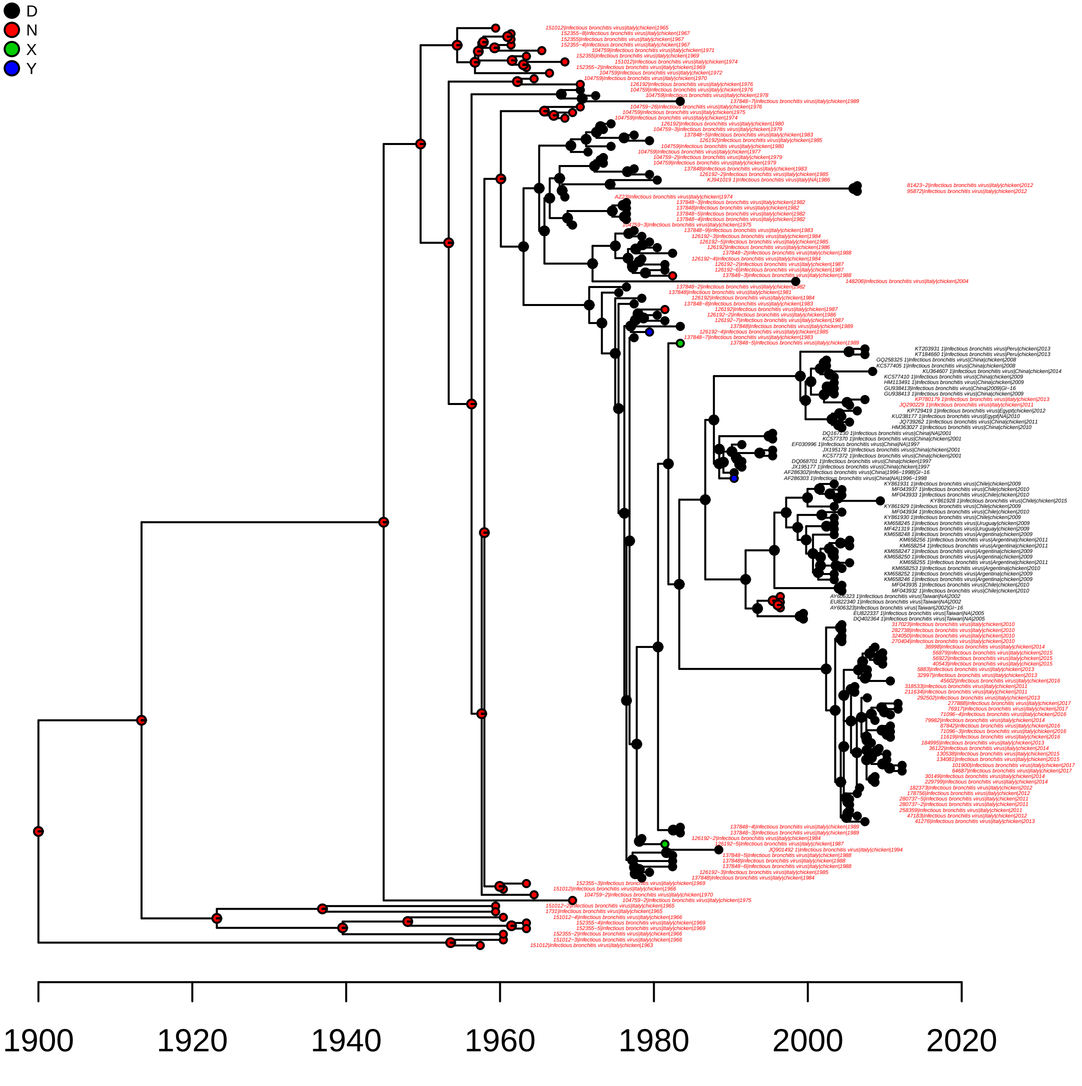

86

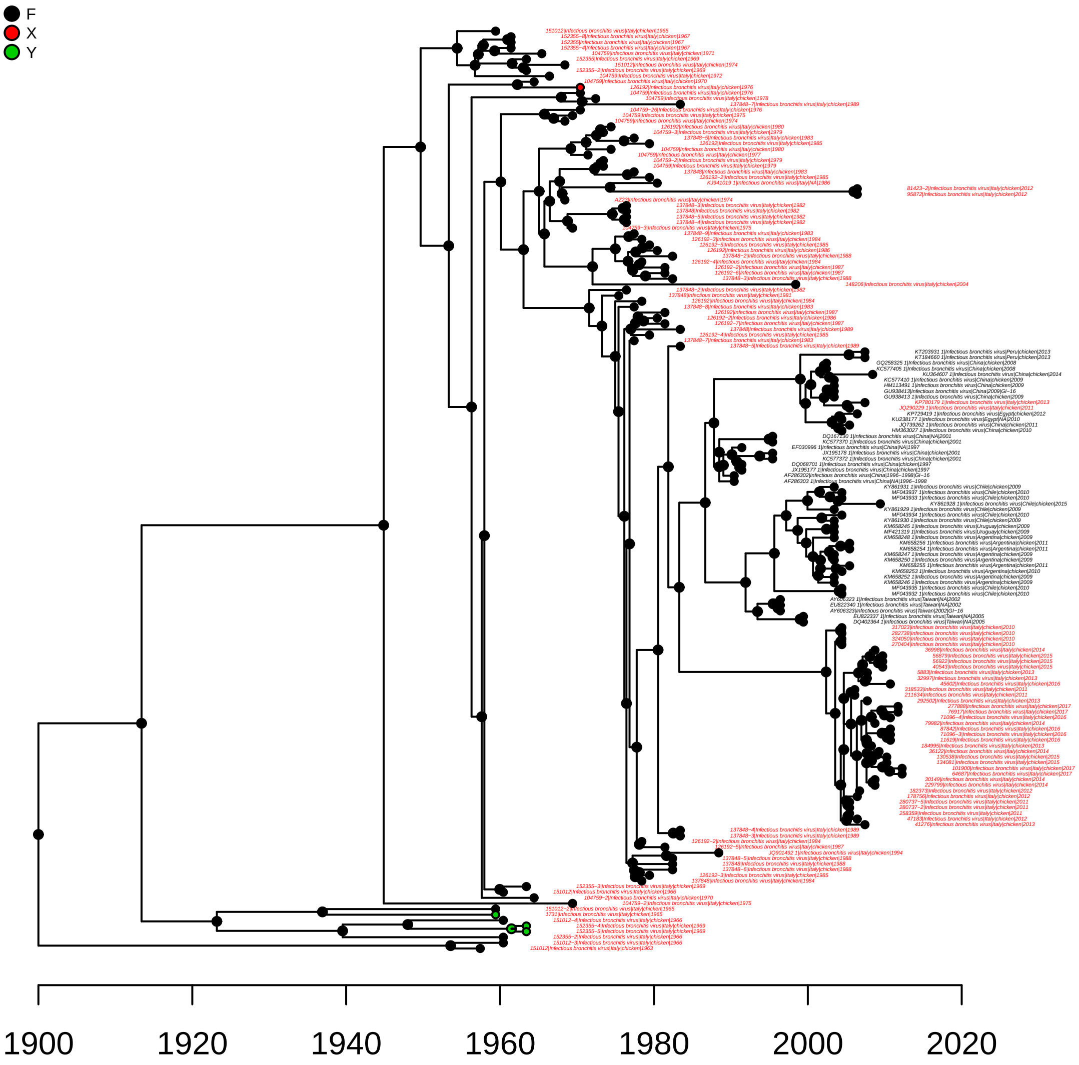

87

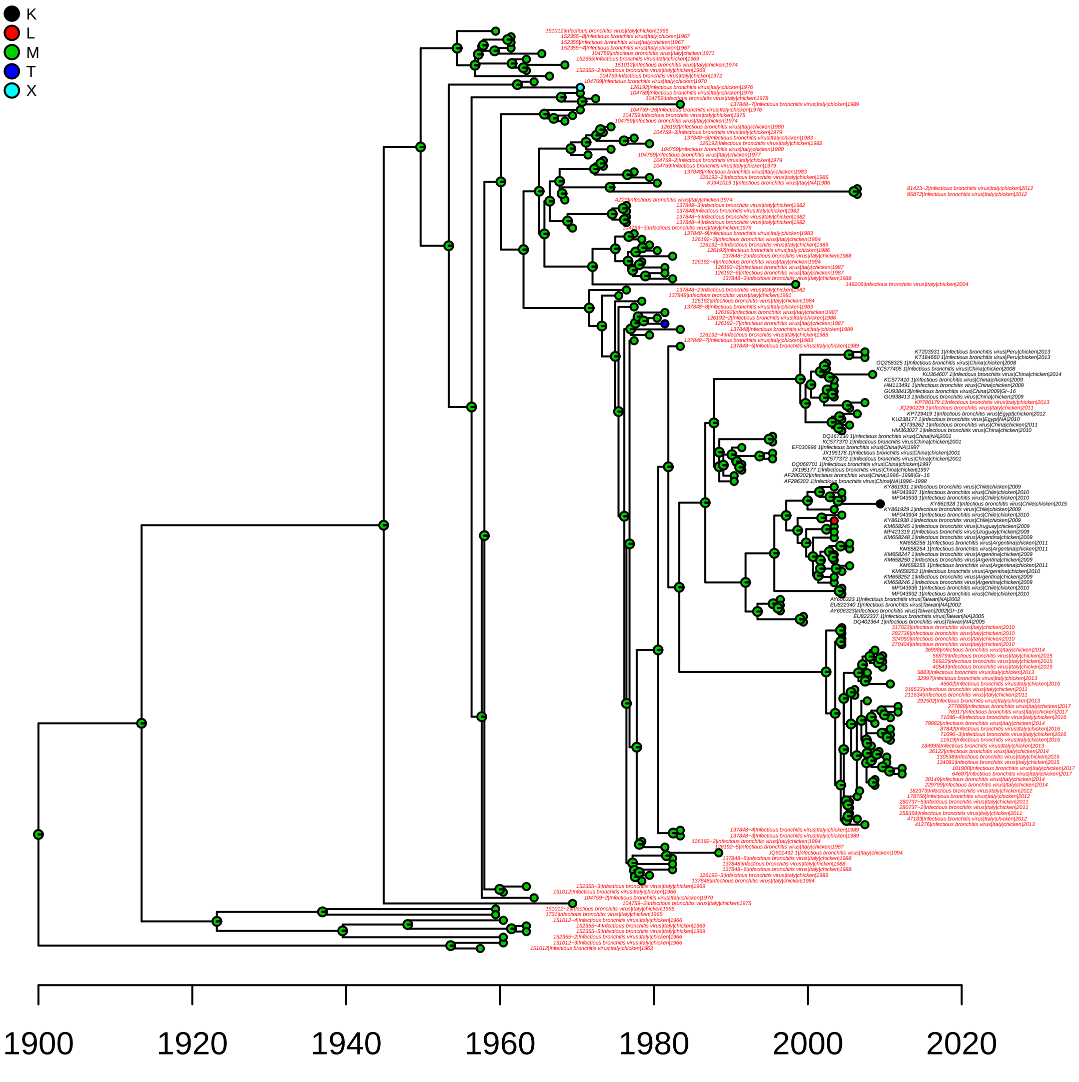

88

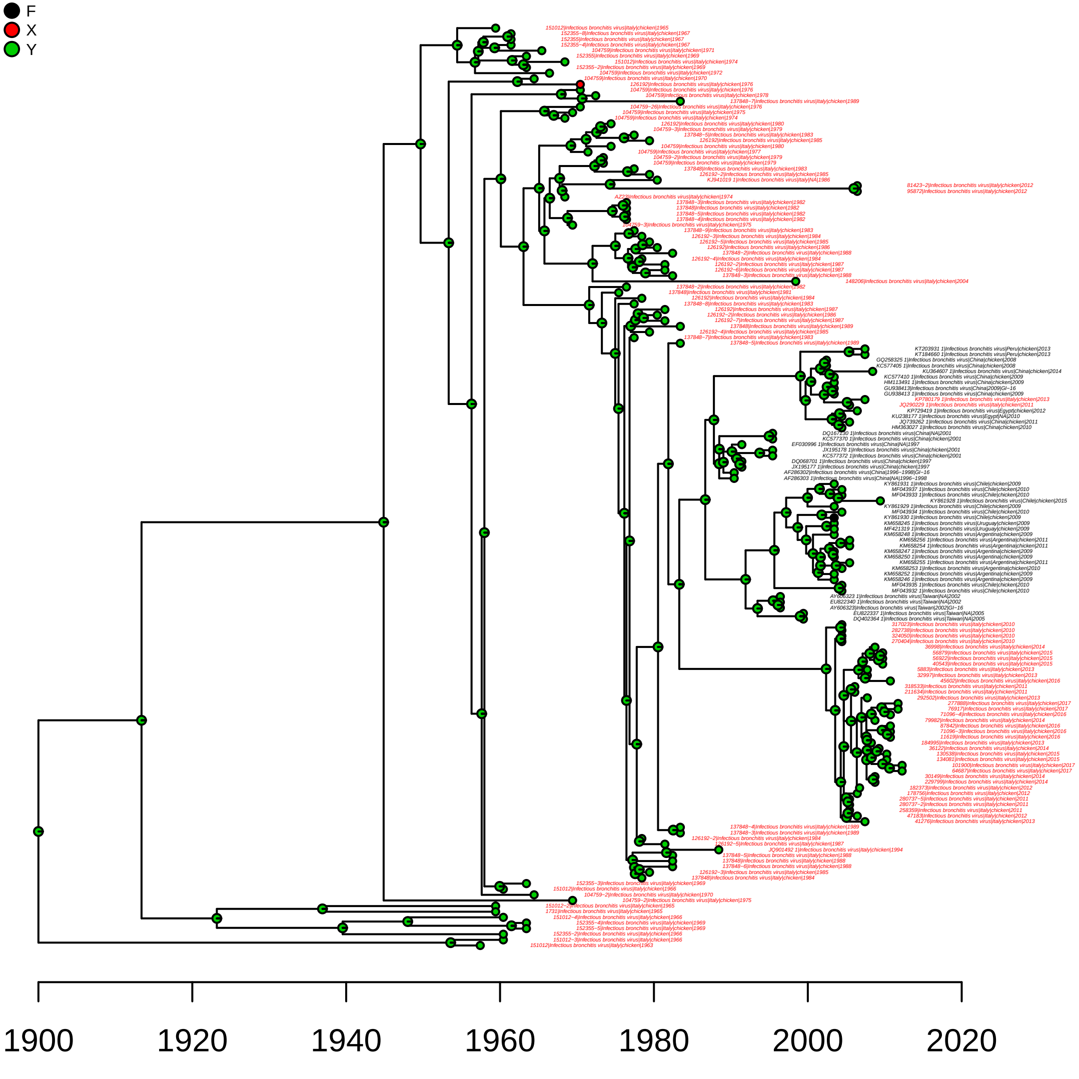

89

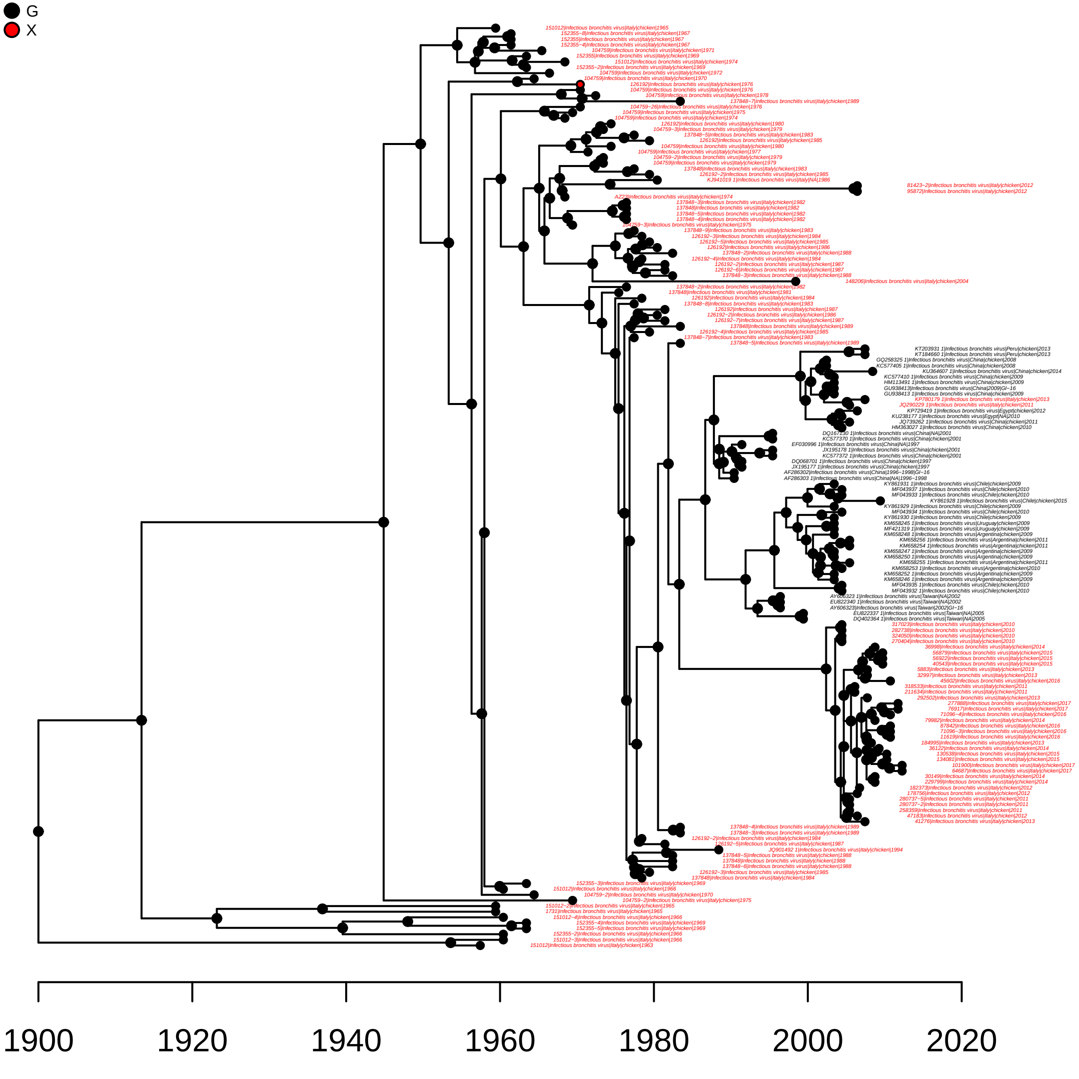

90

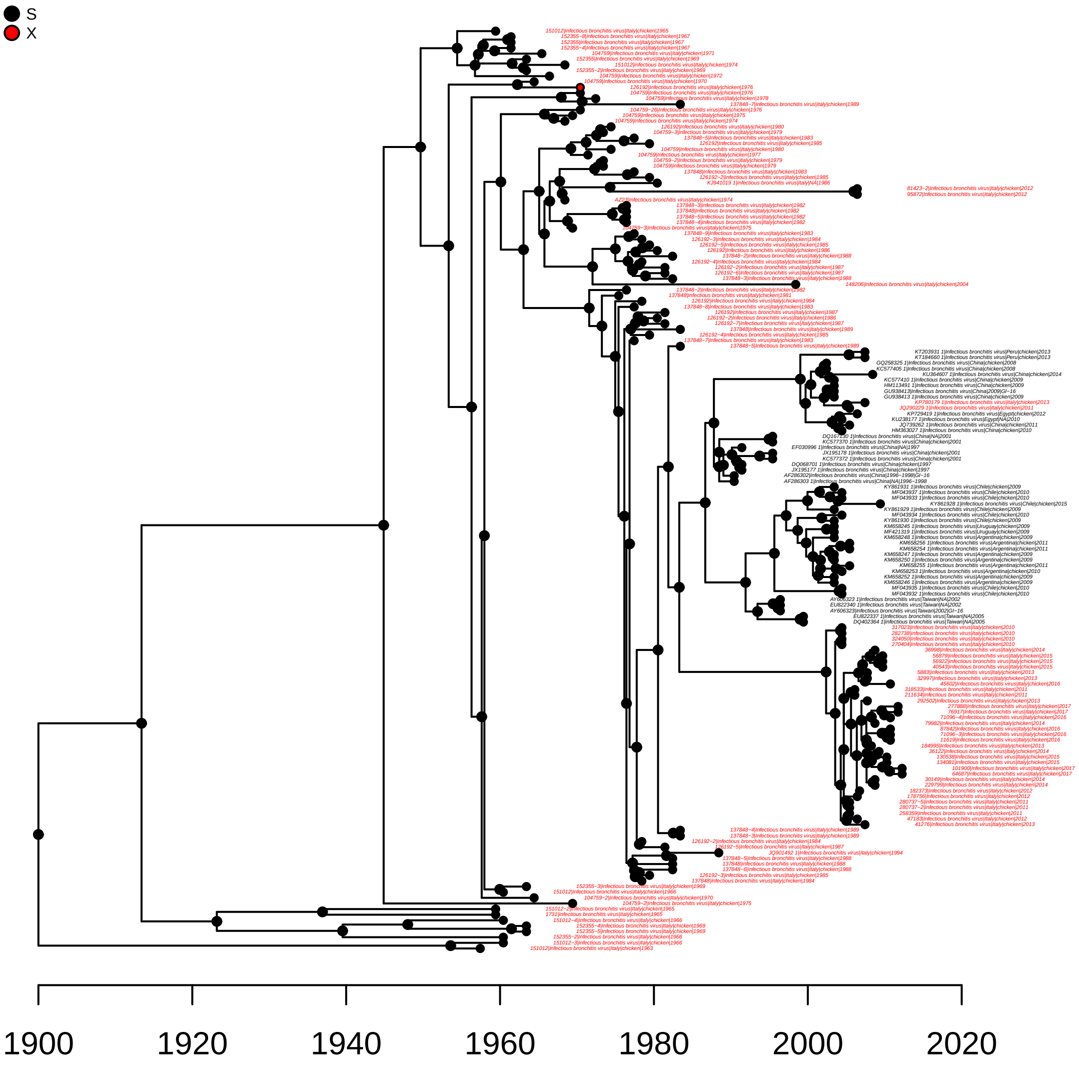

91

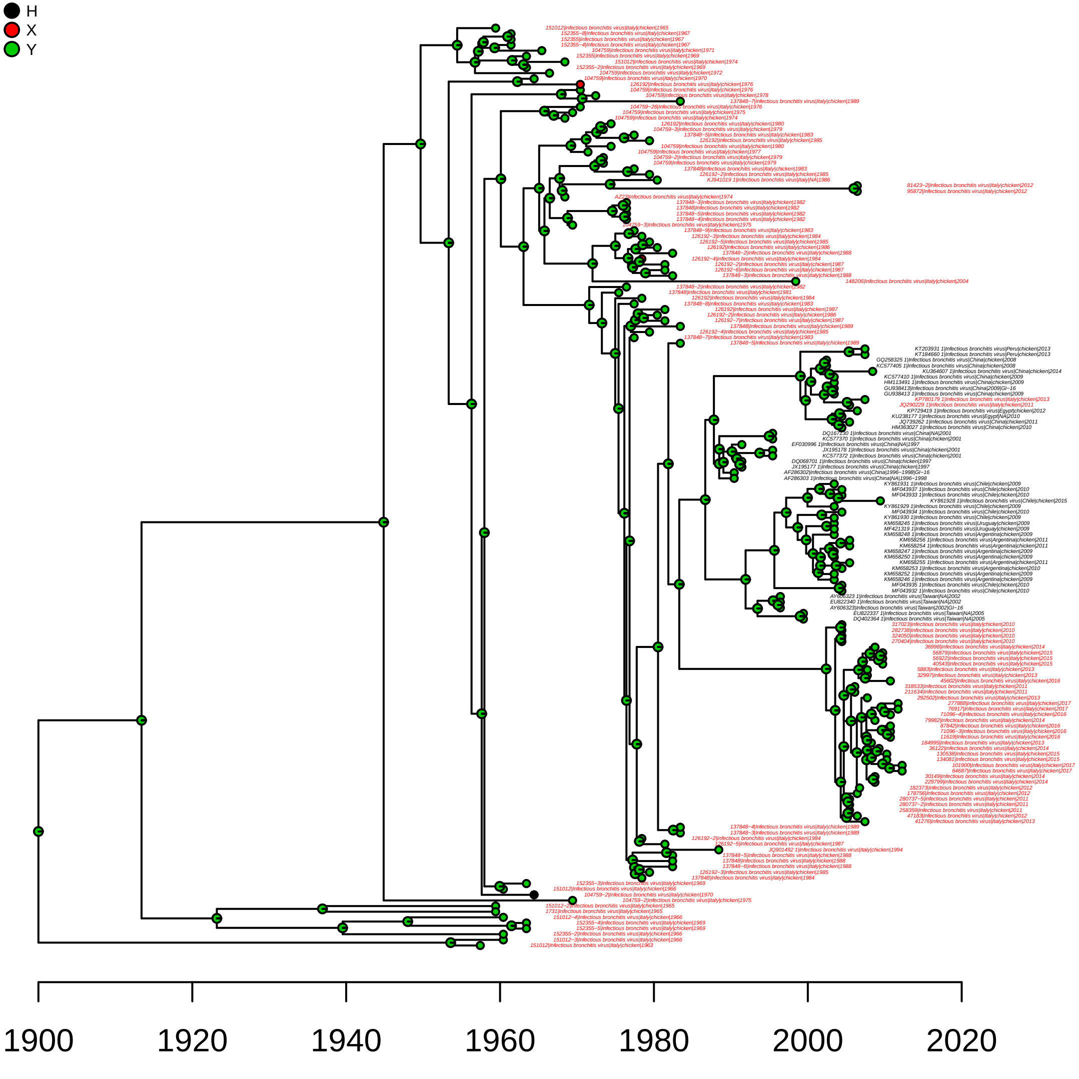

92

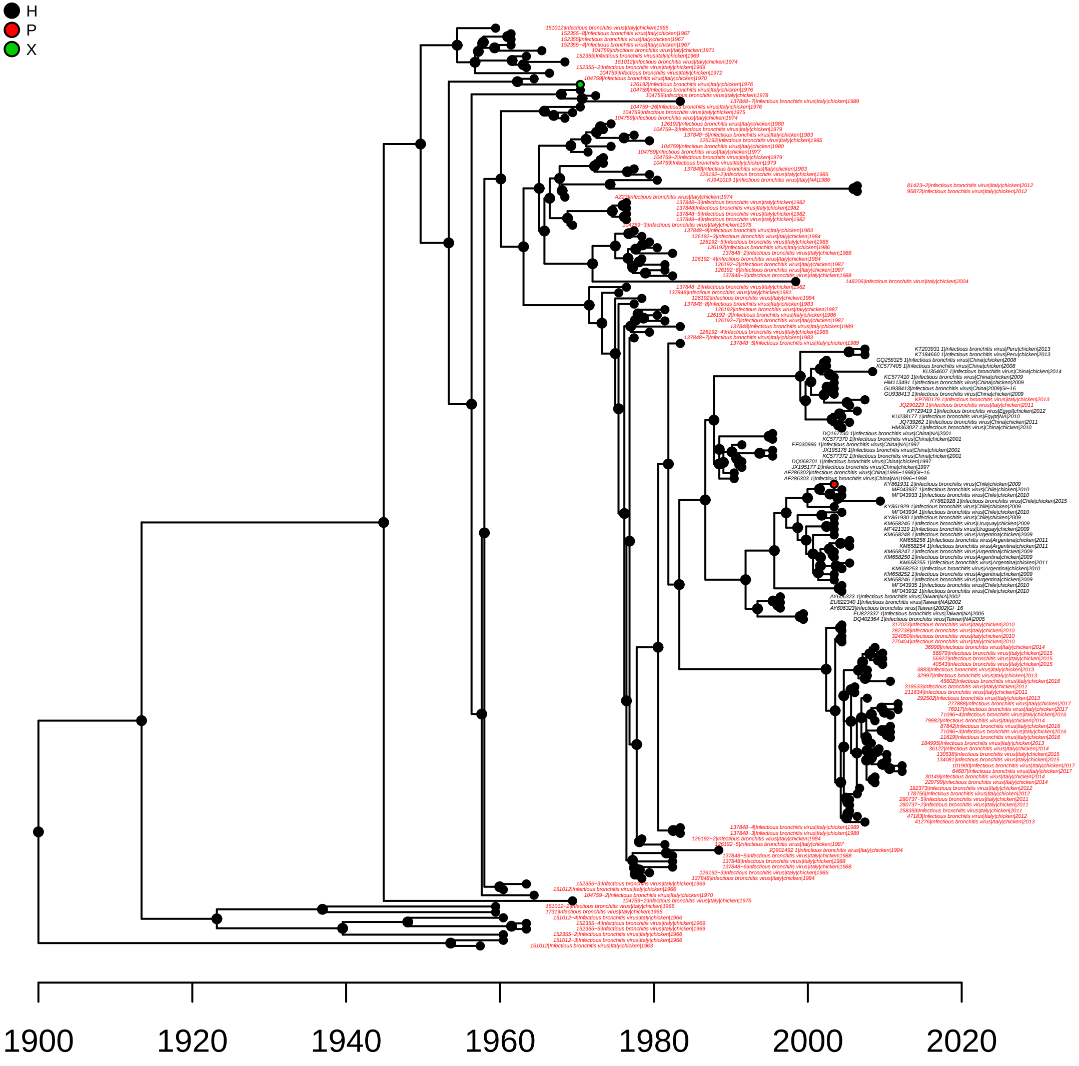

93

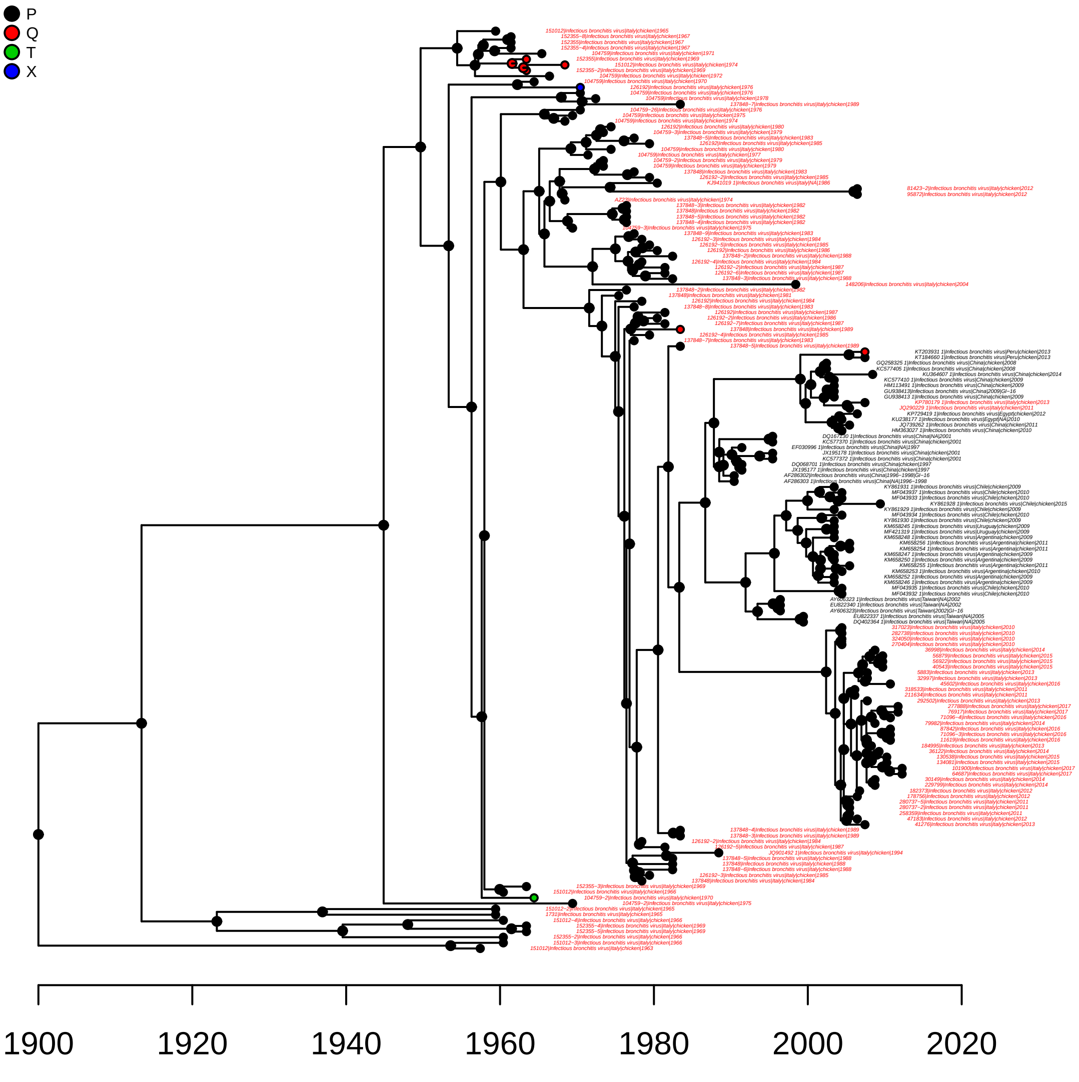

94

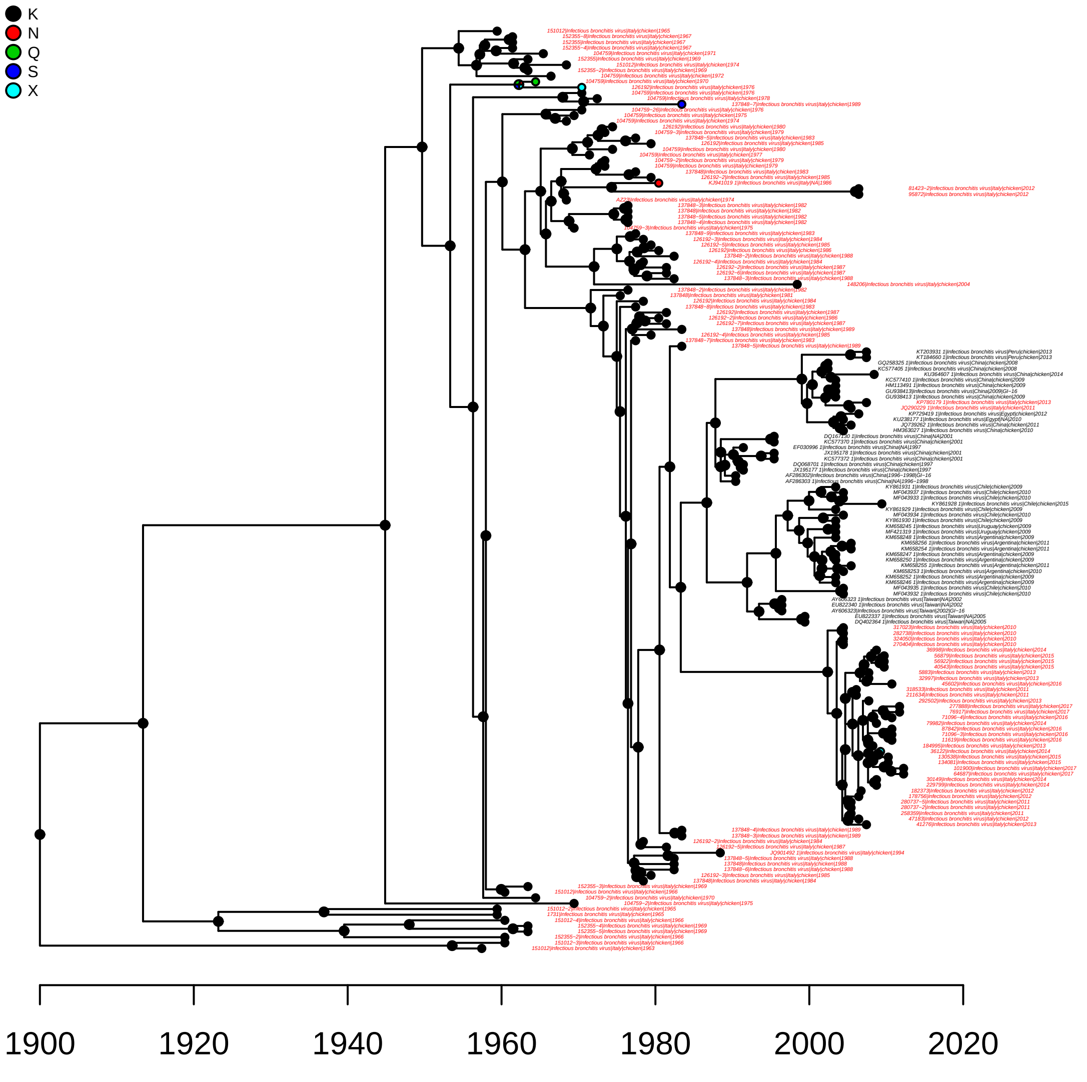

95

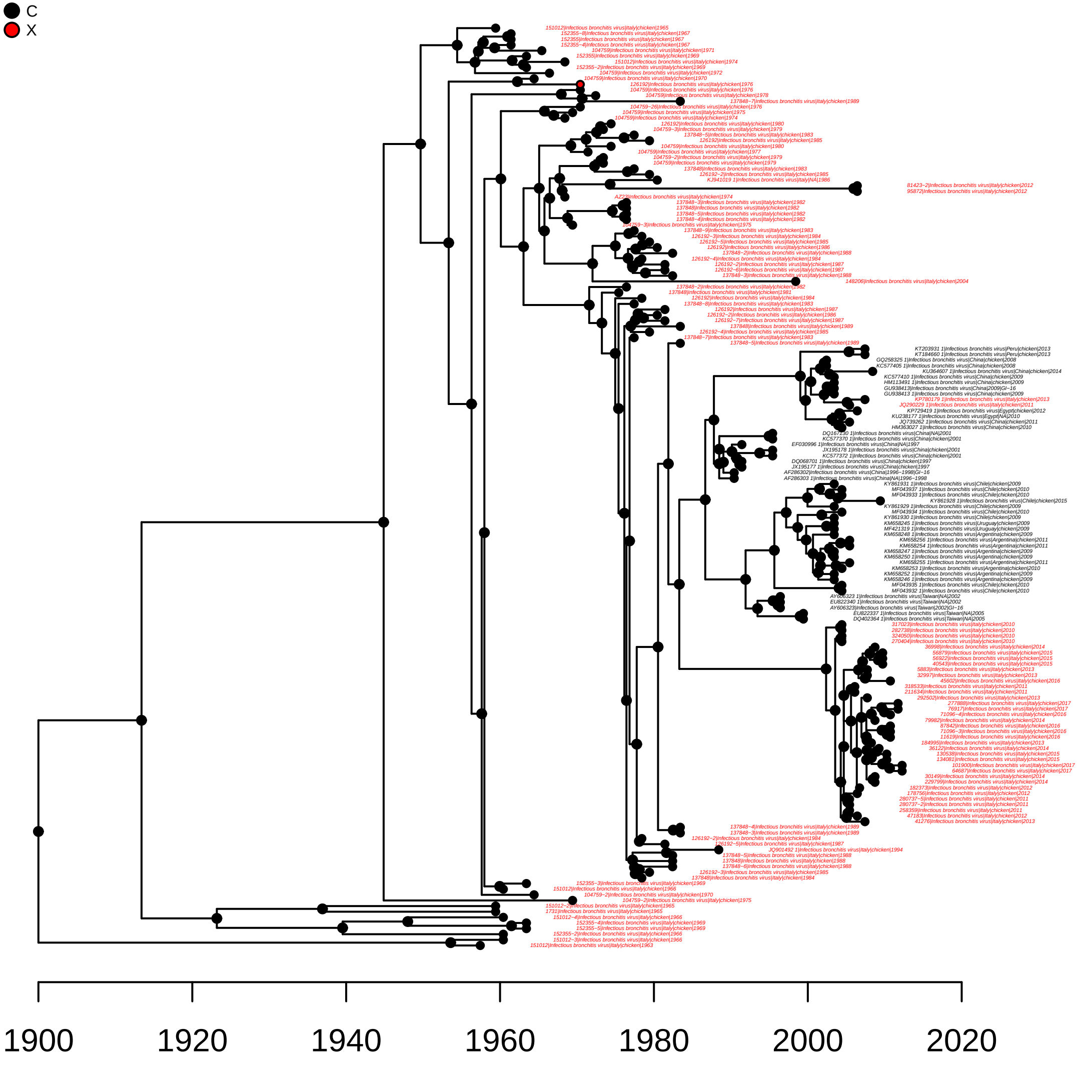

96

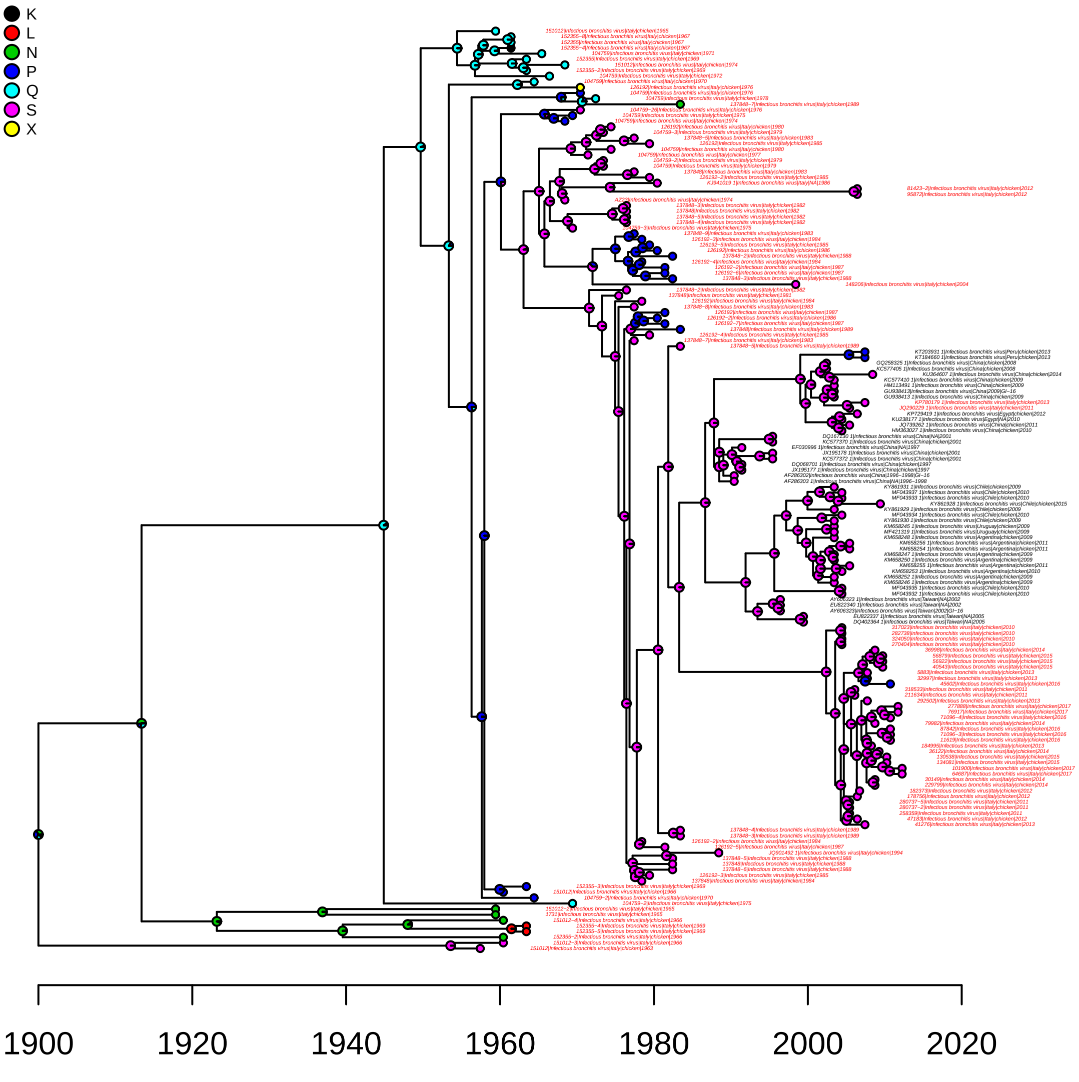

97

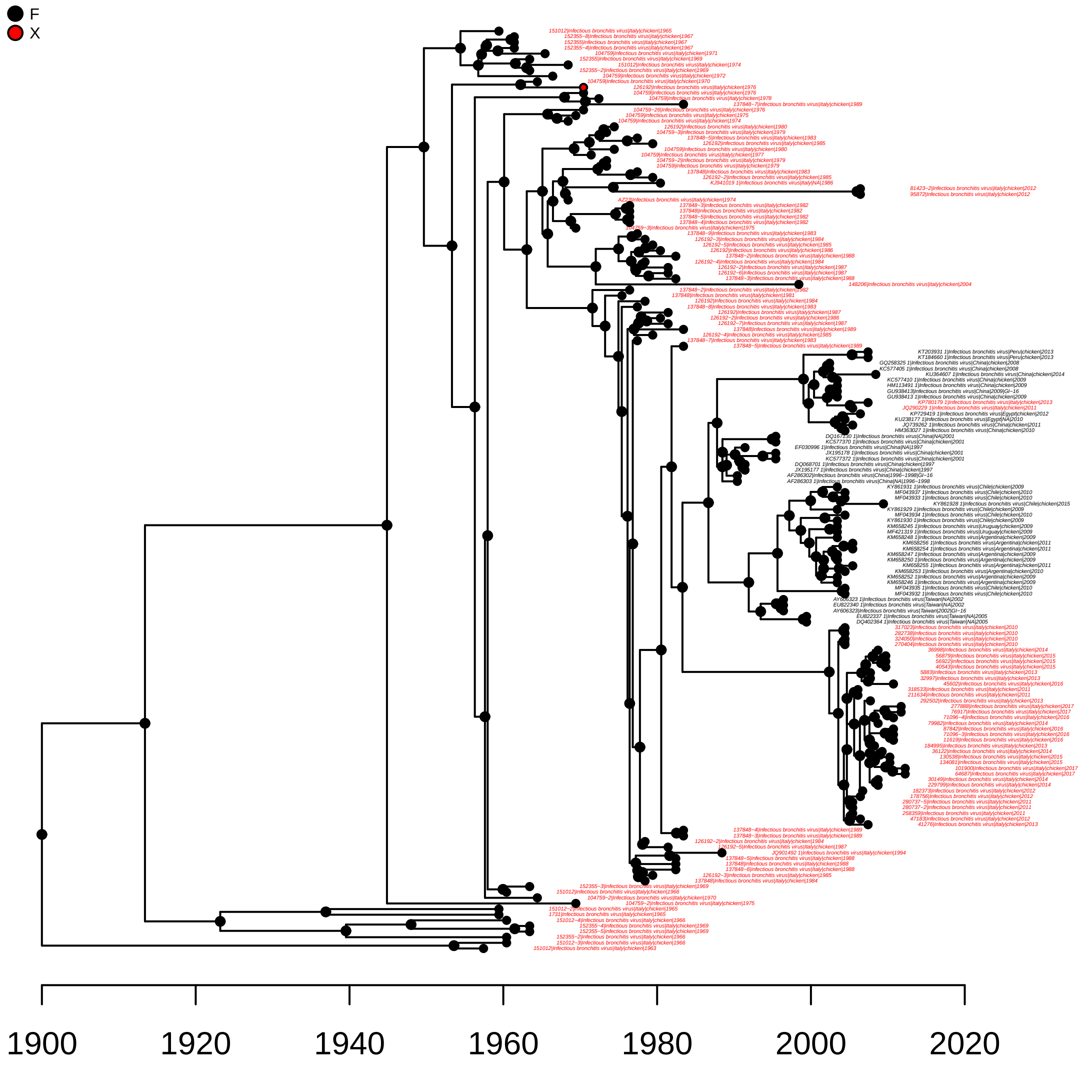

98

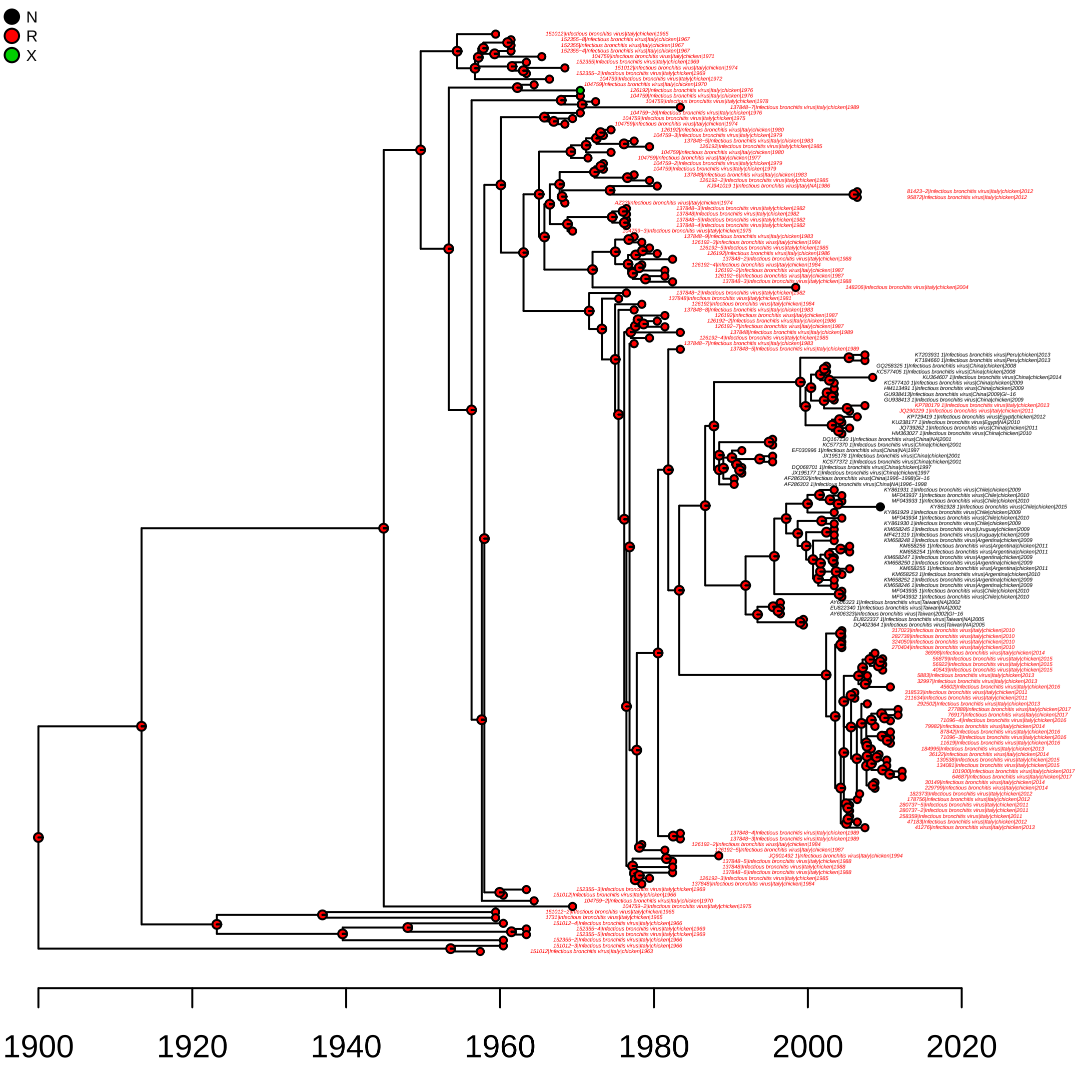

99

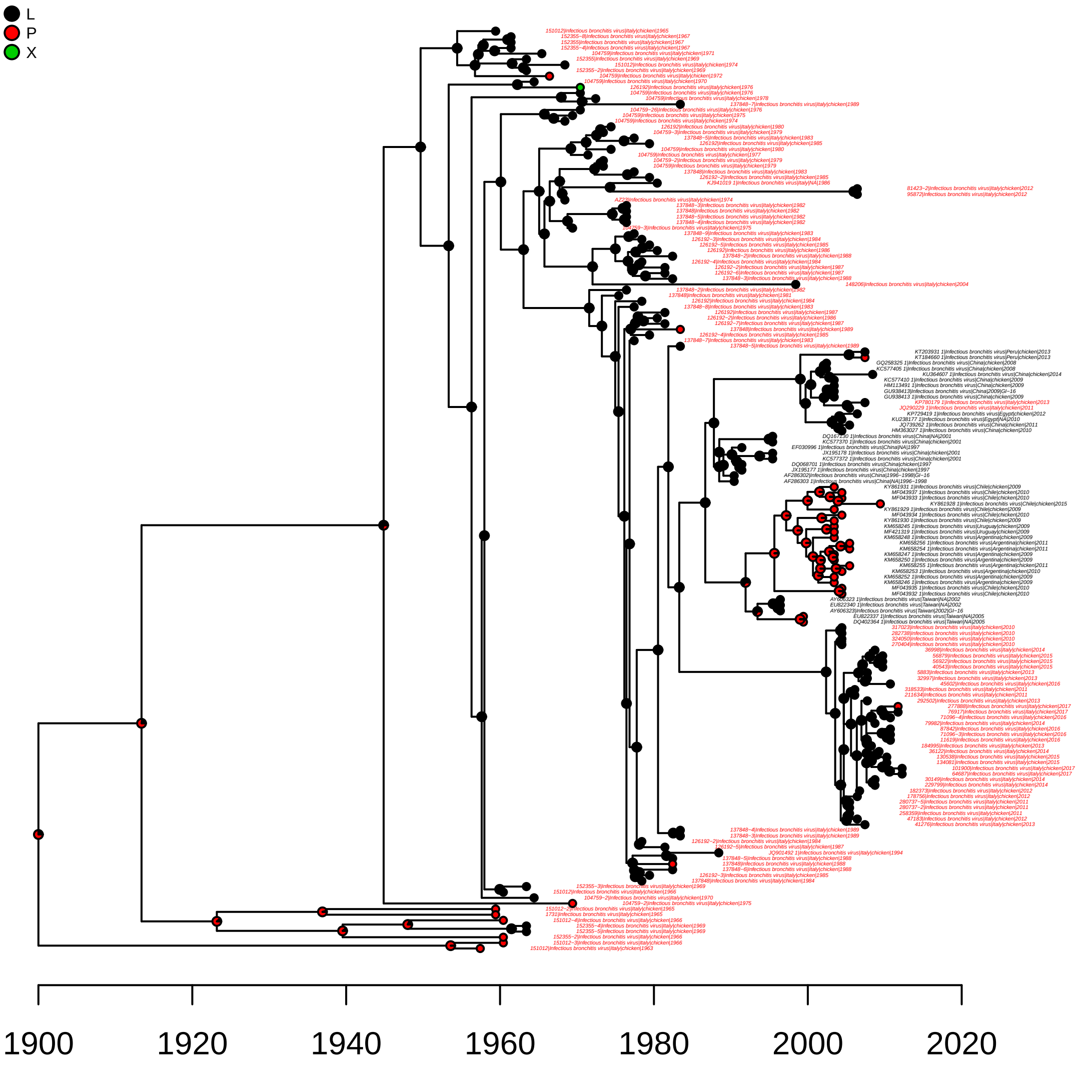

100

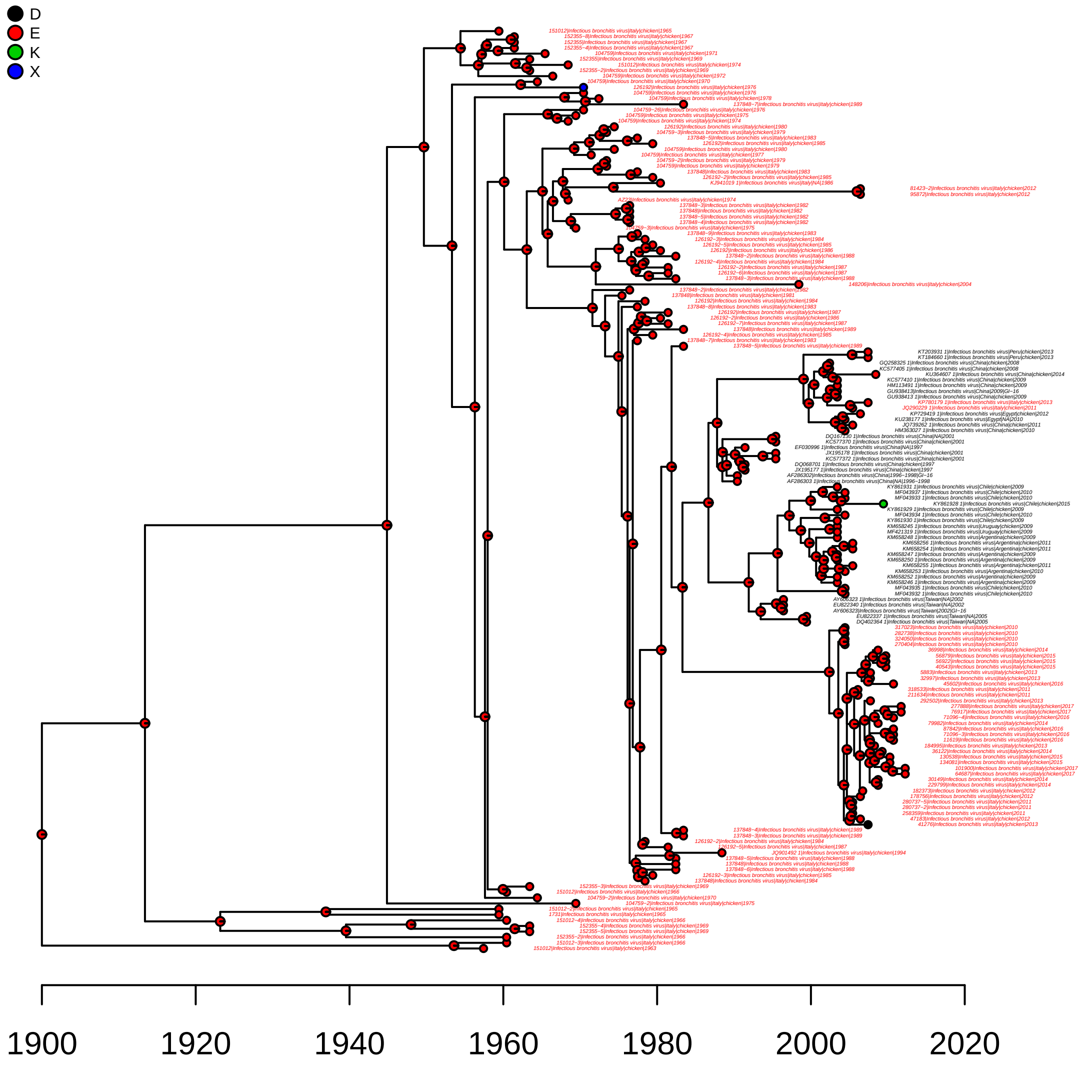

101

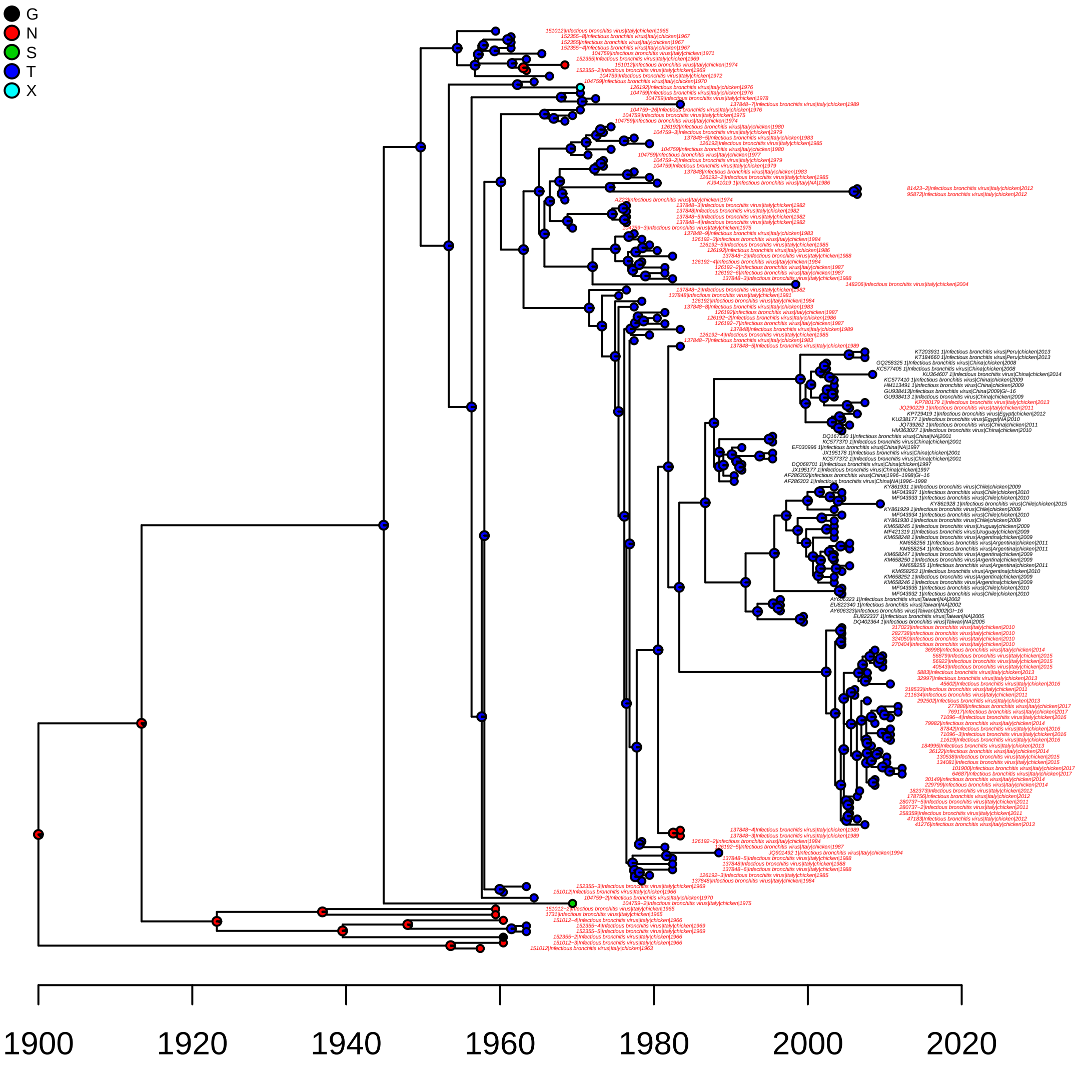

102

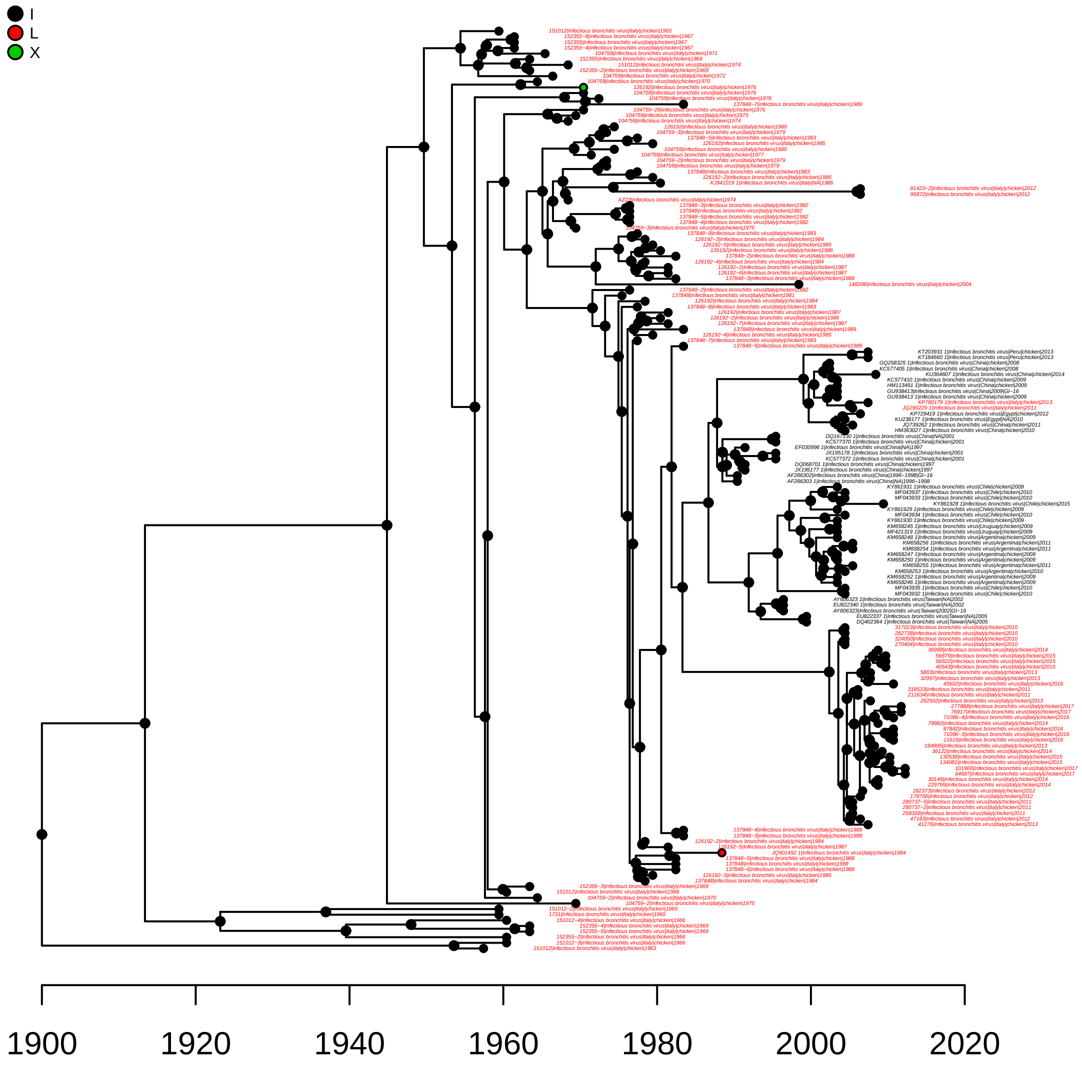

103

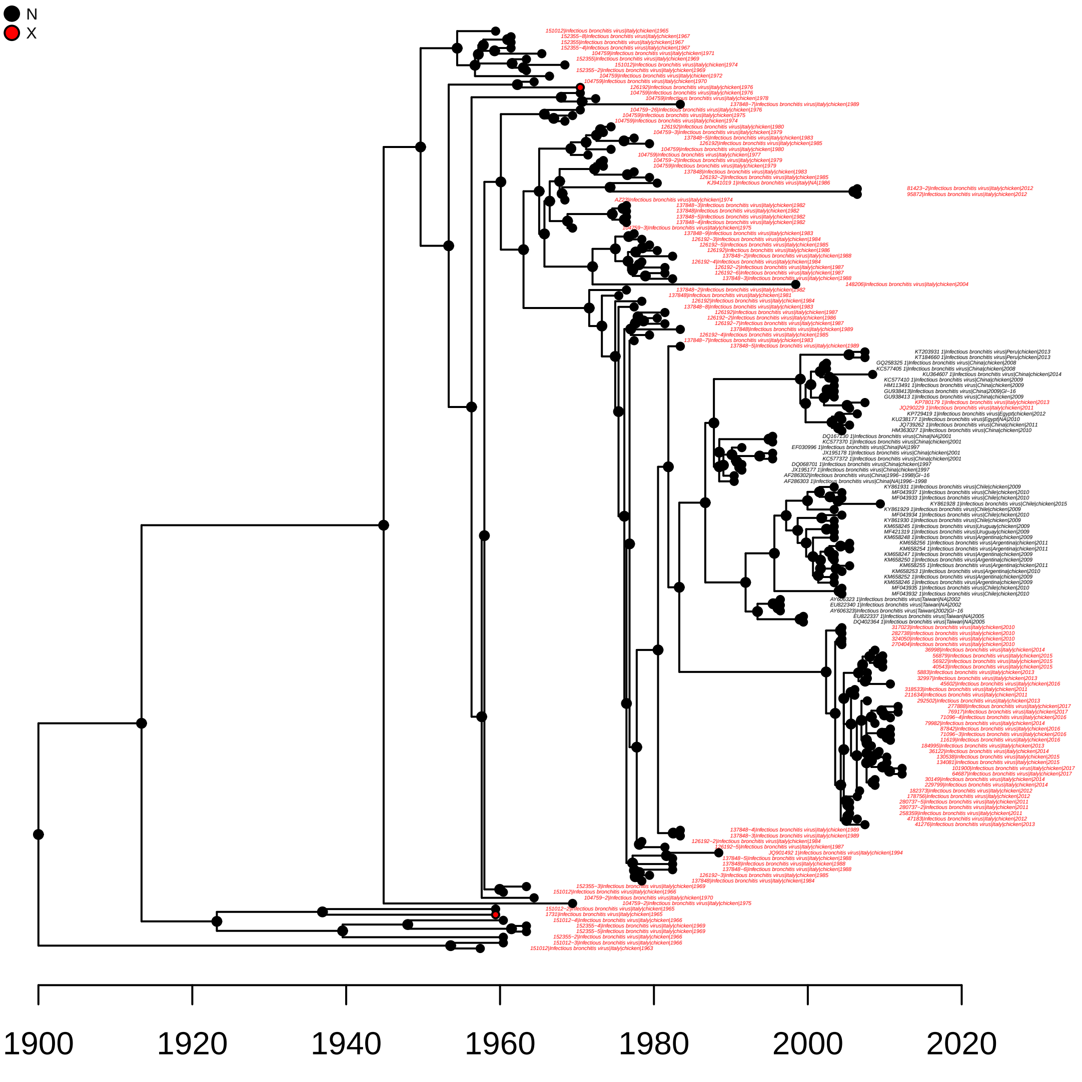

104

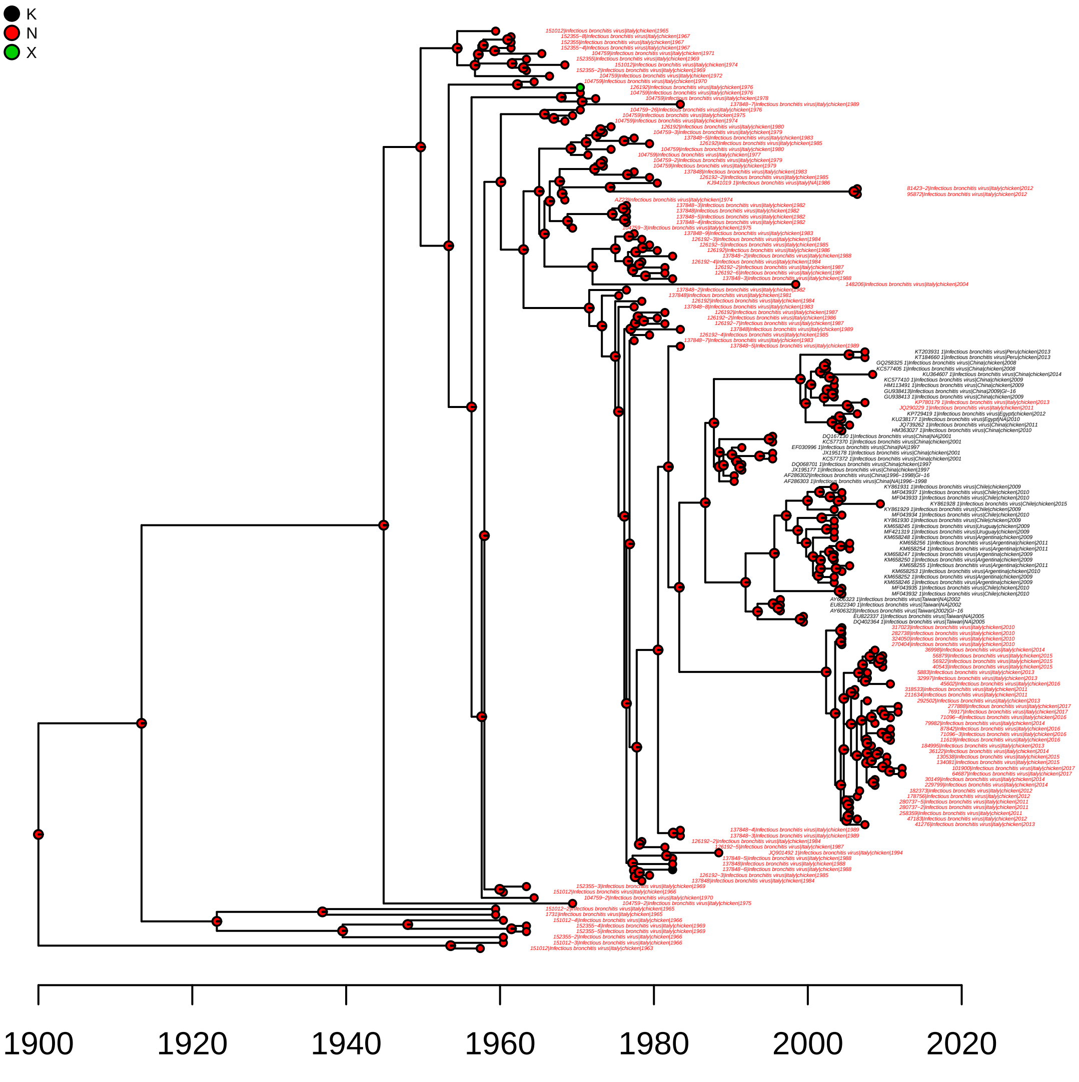

105

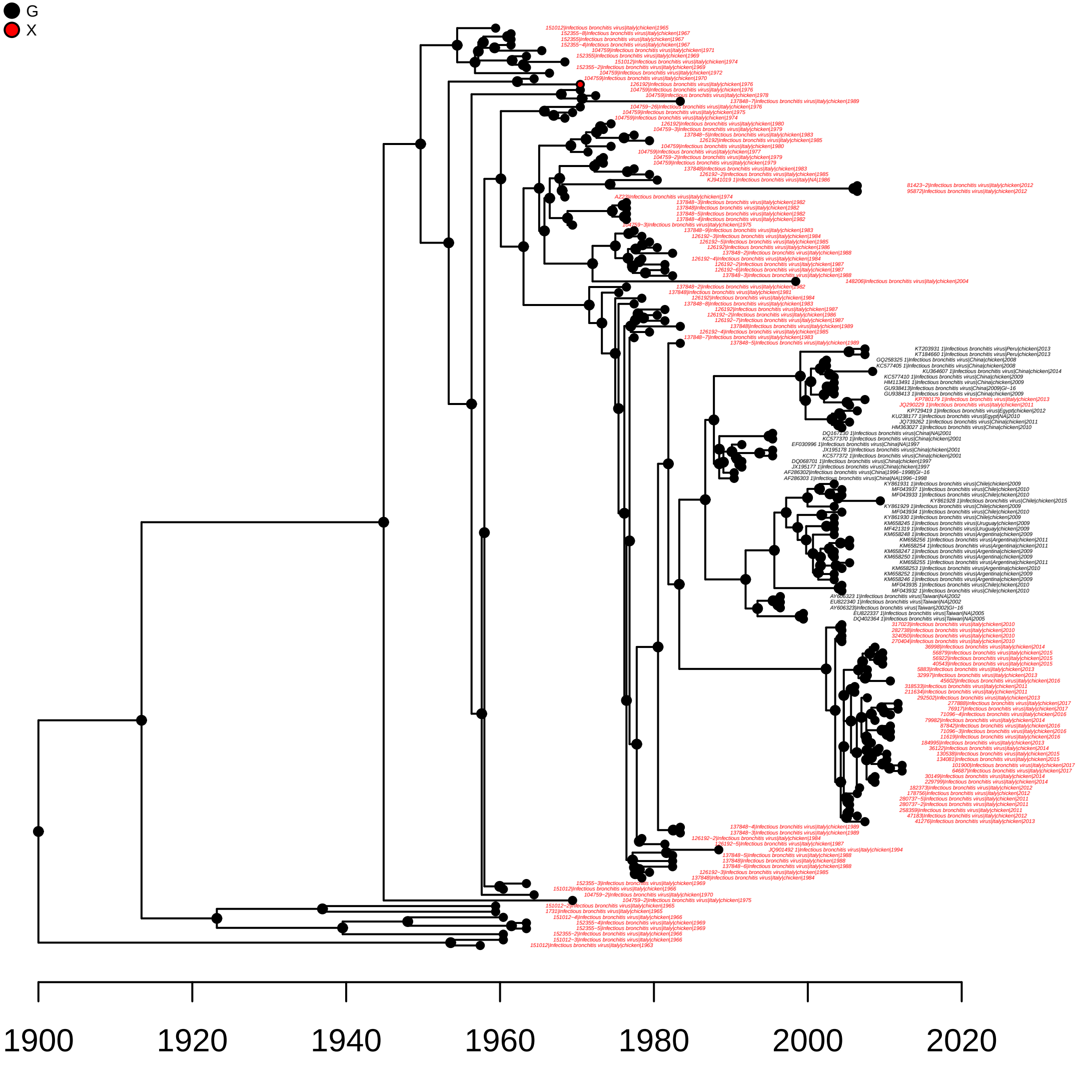

106

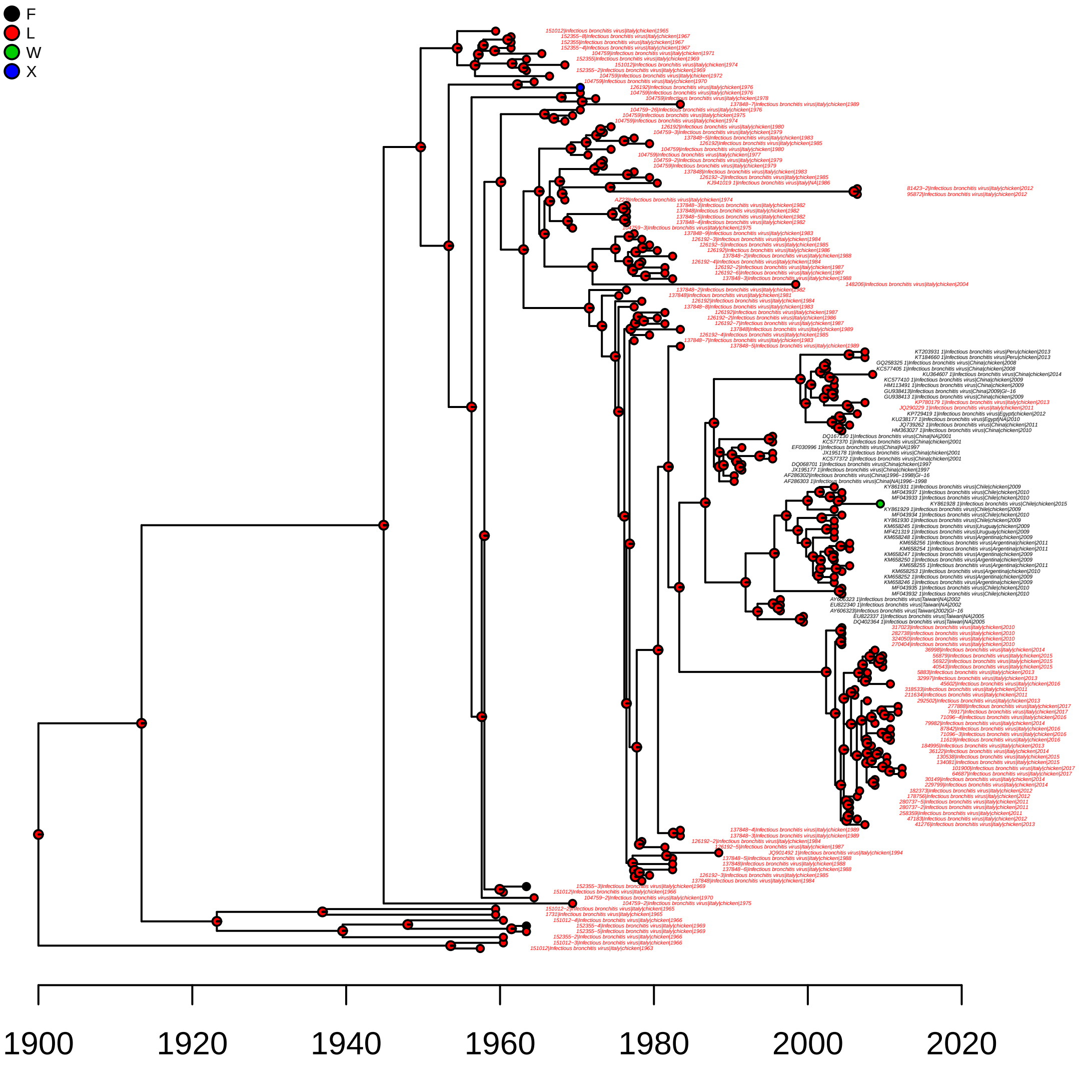

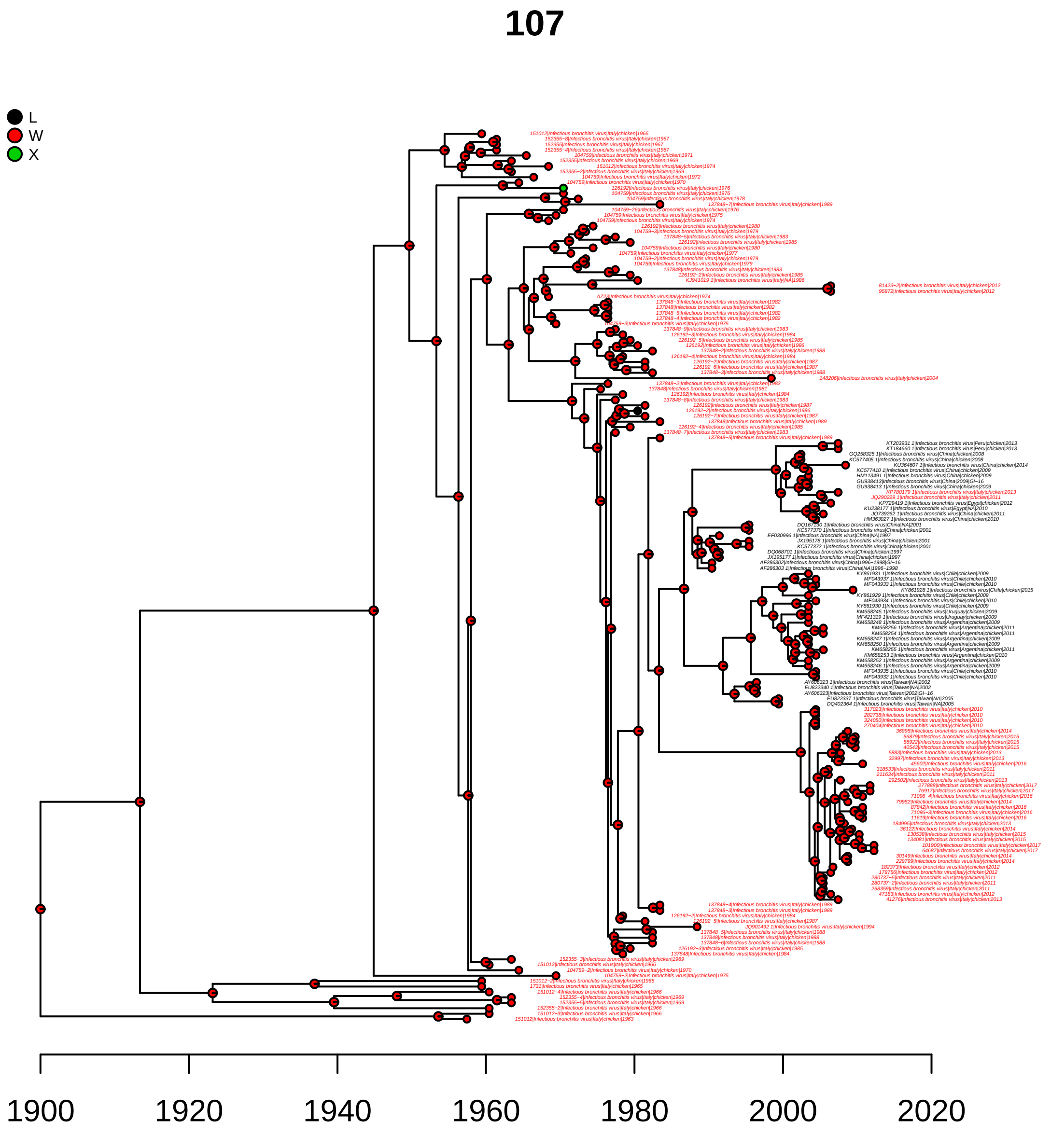

107
L
W
X
1900
1920
1940
1960
1980
2000
2020

108

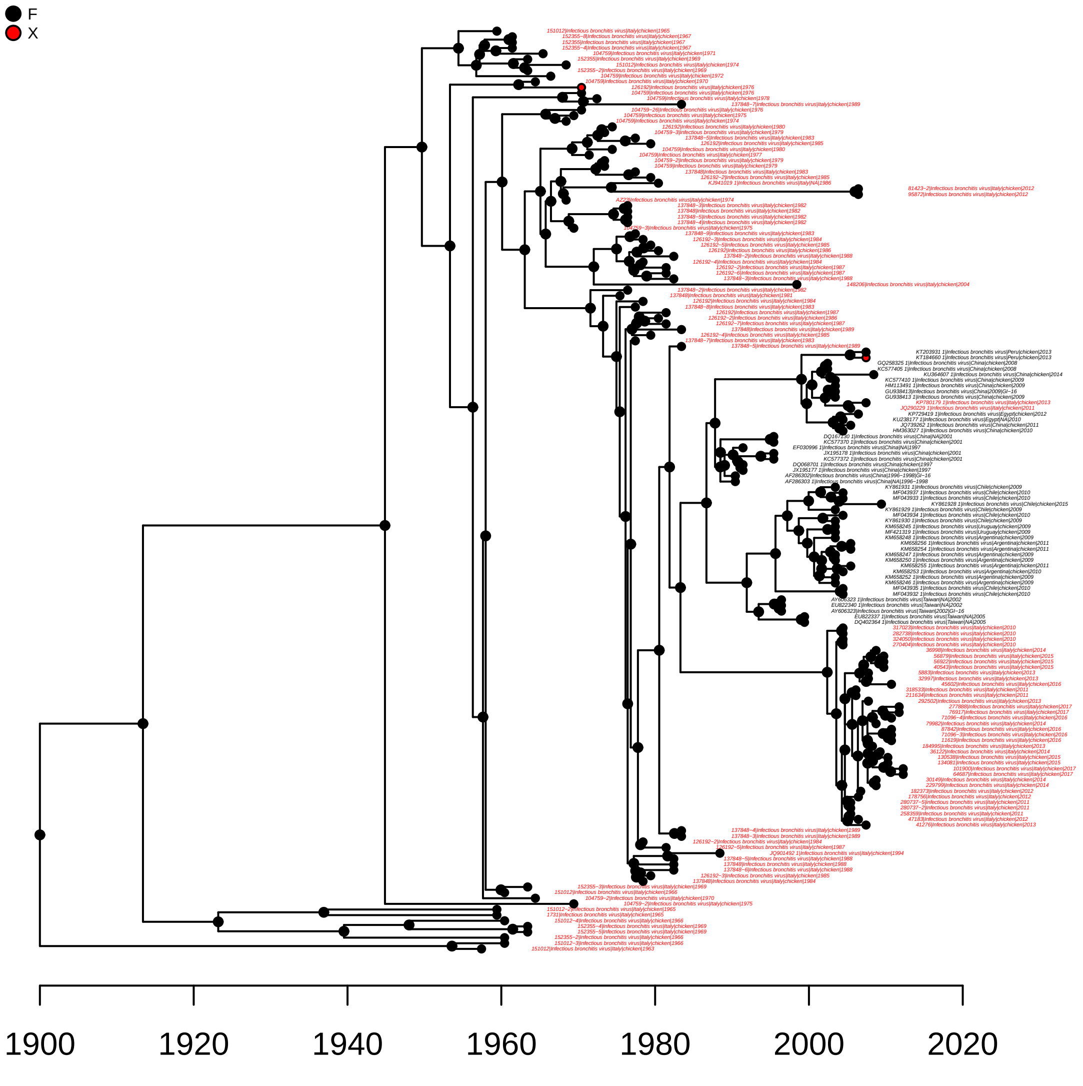

109

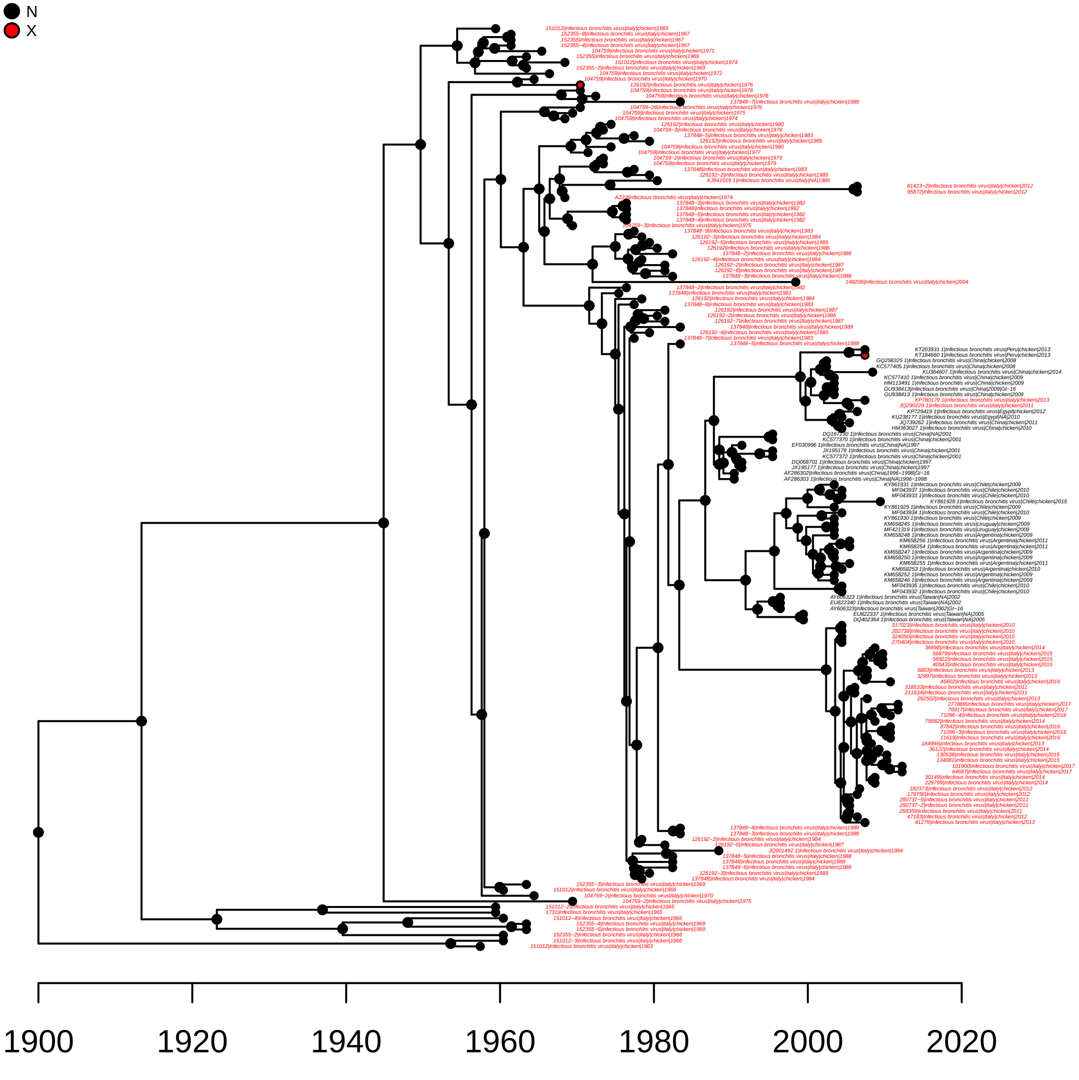

110

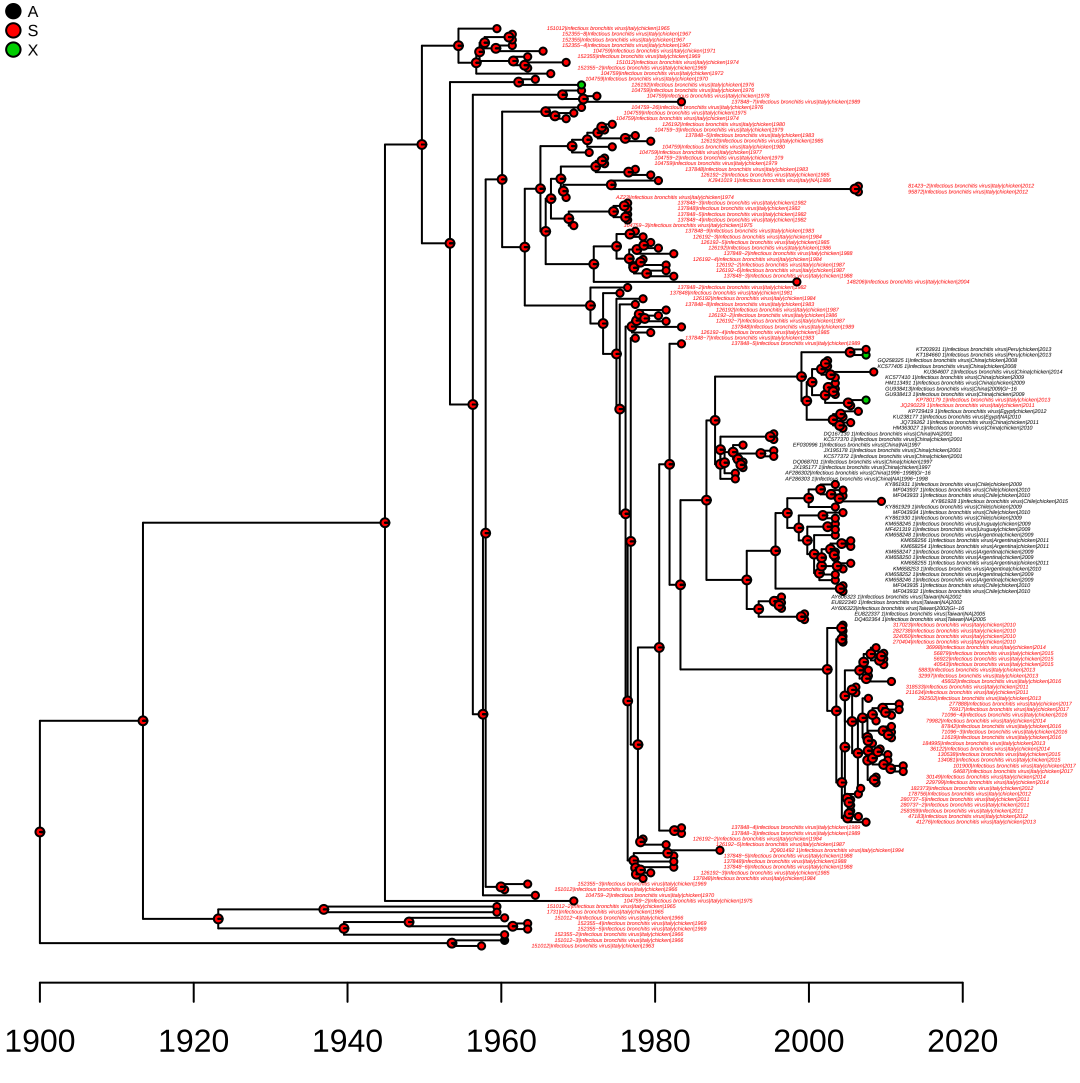

111

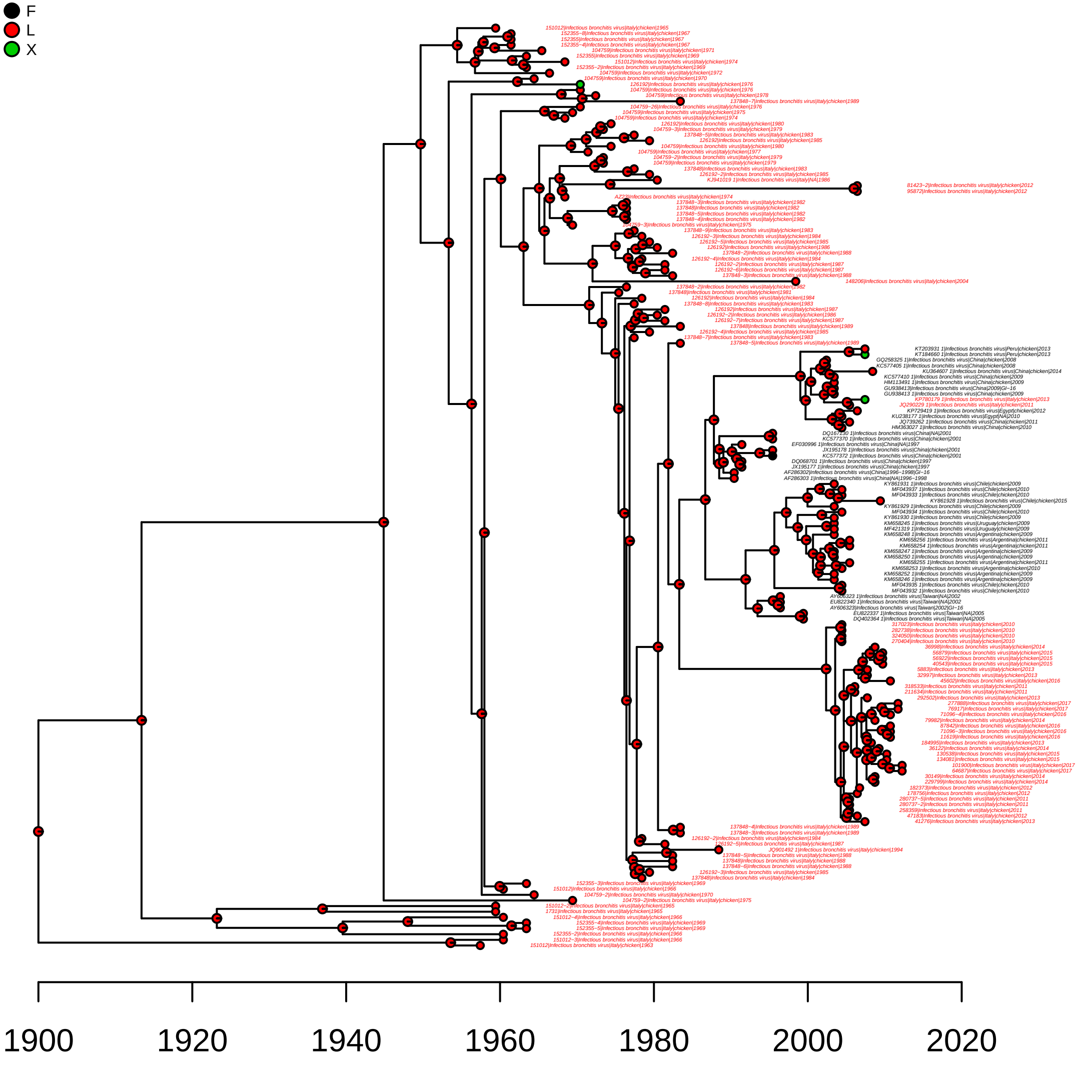

112

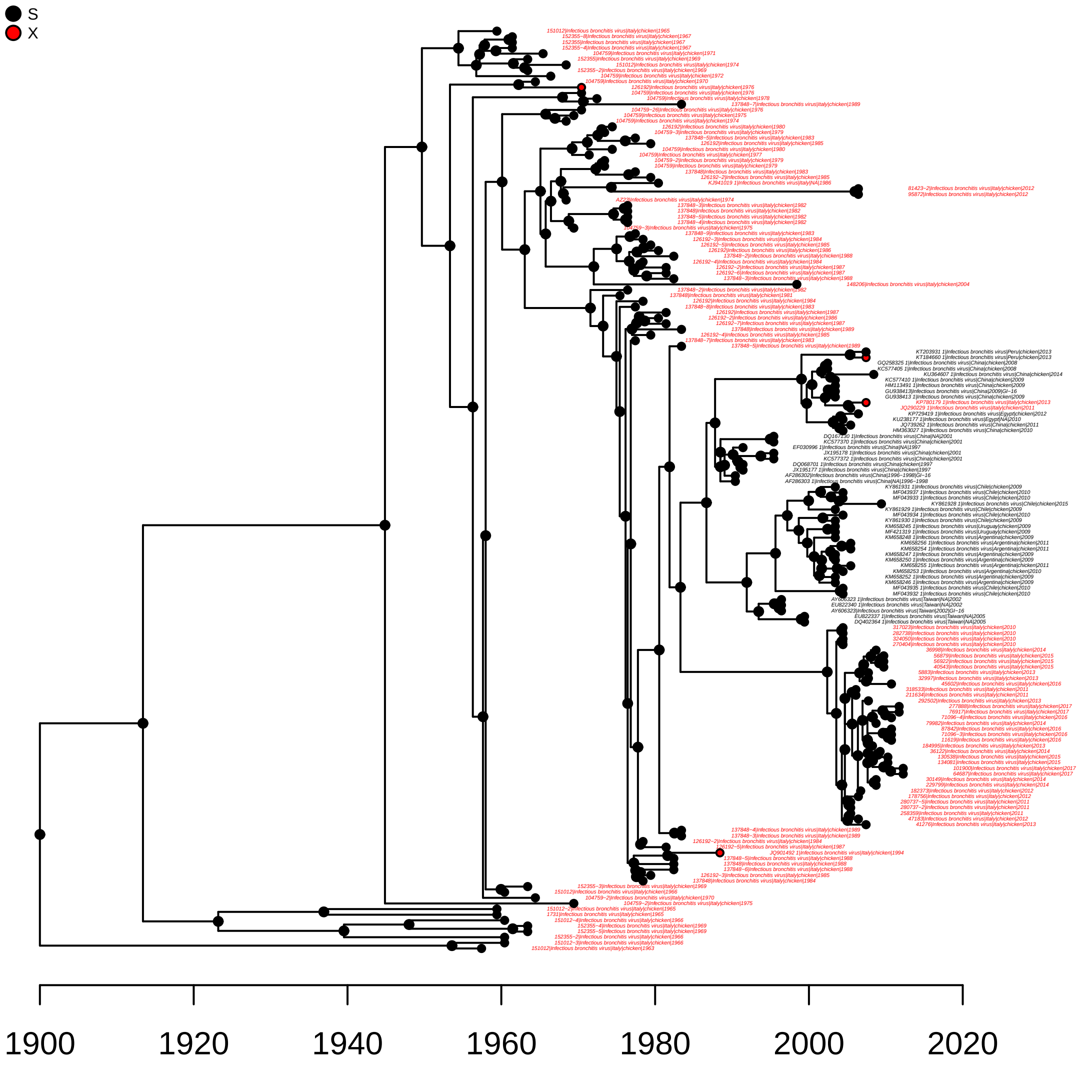

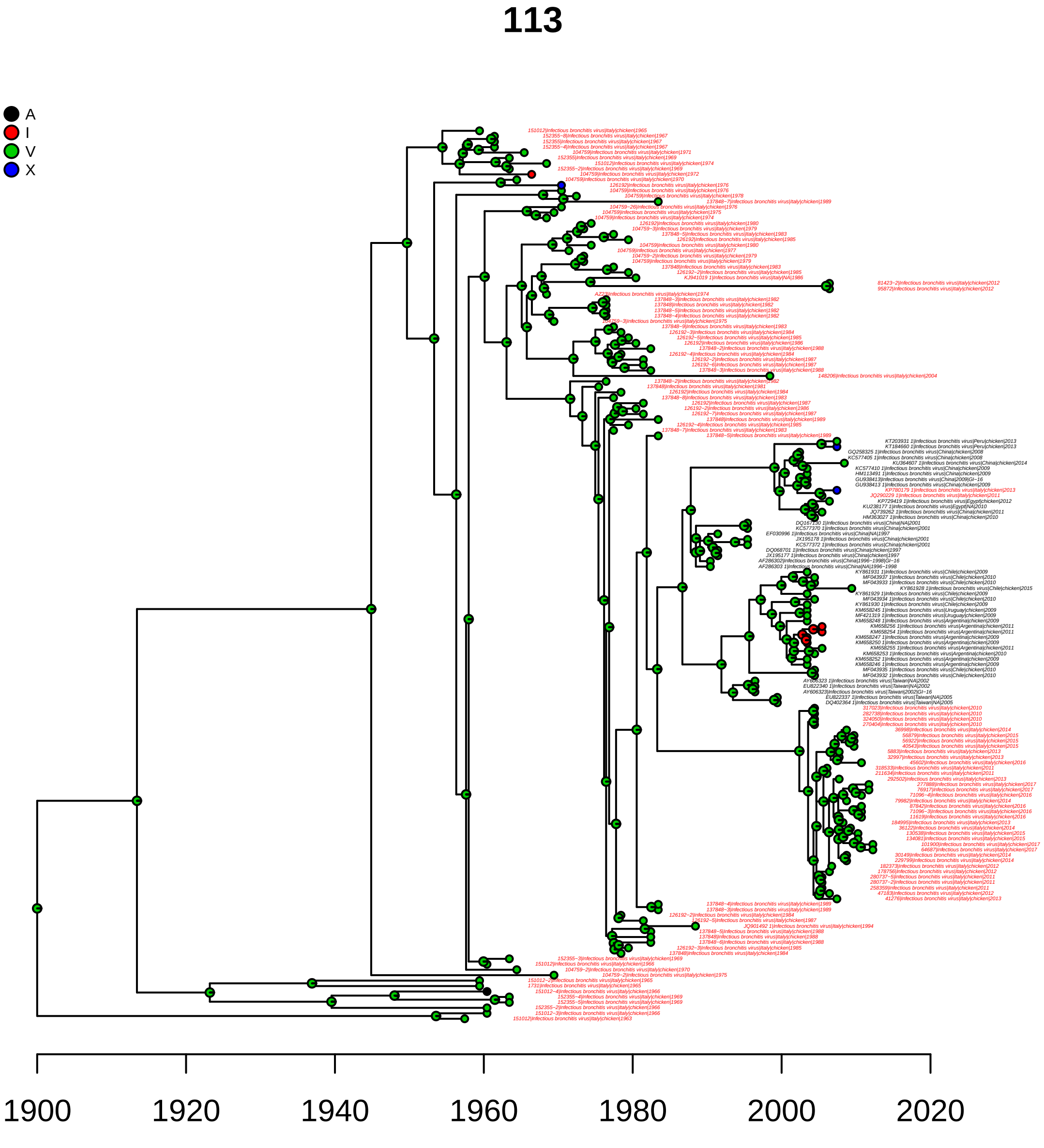
113
A
I
V
X
1900
1920
1940
1960
1980
2000
2020

114

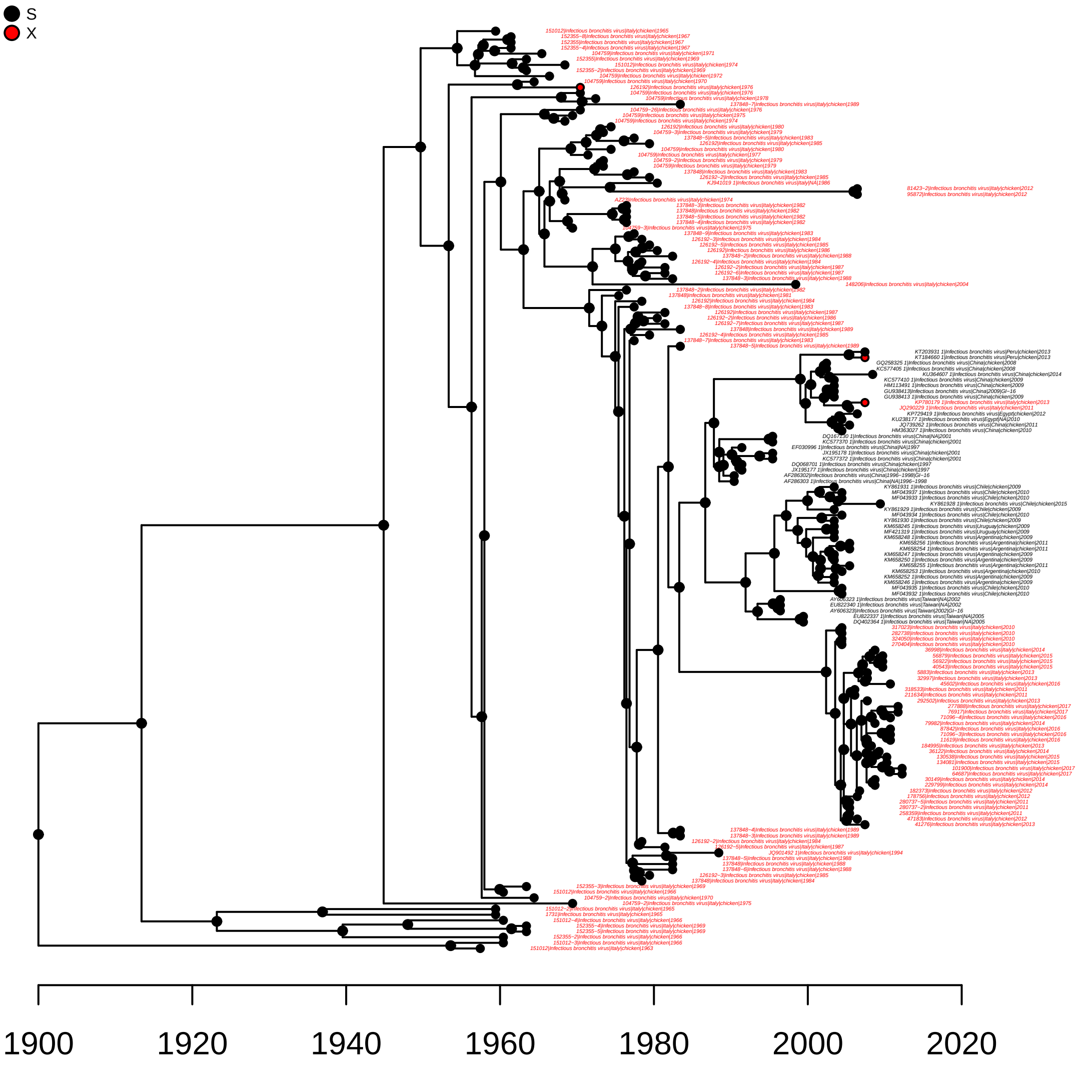

115

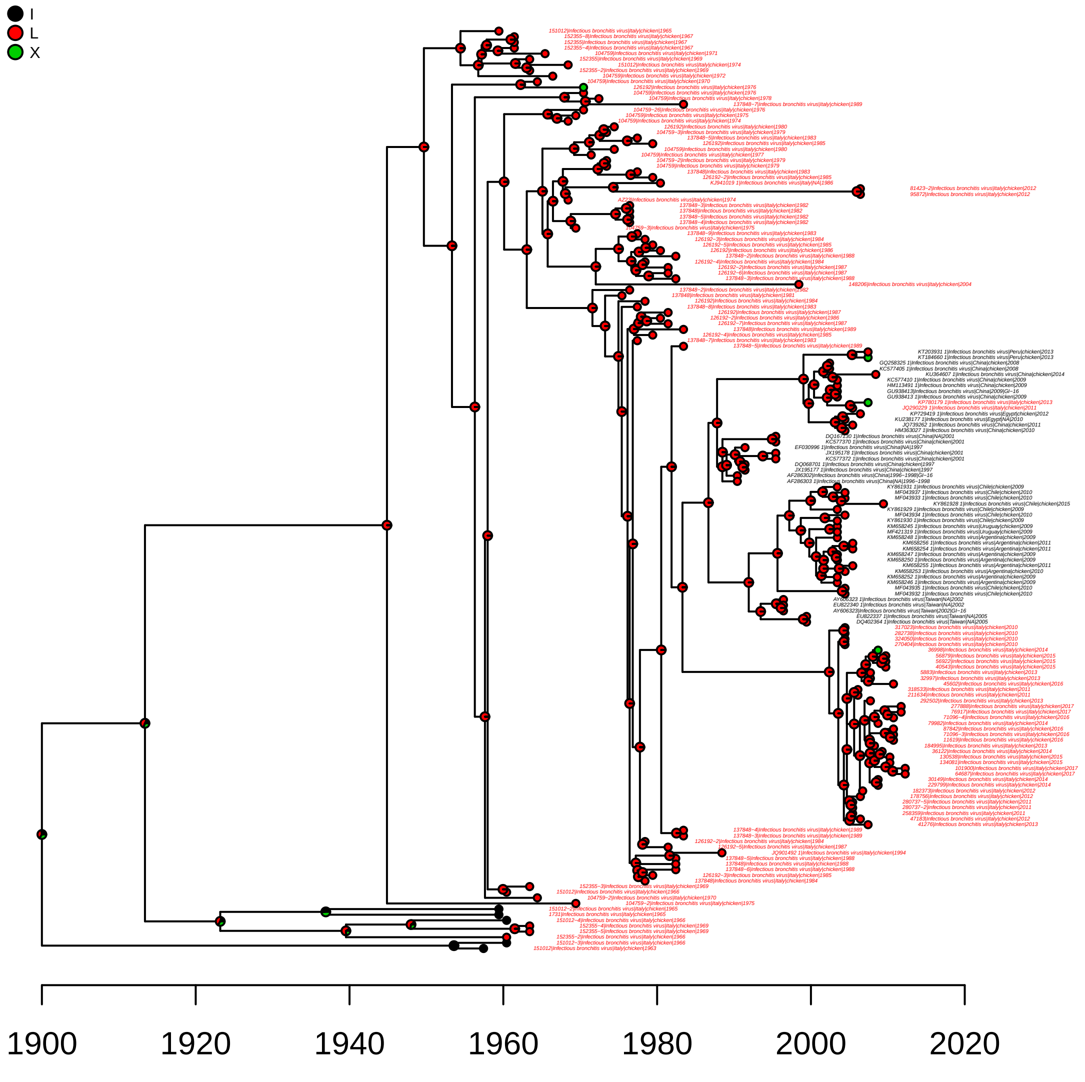

116

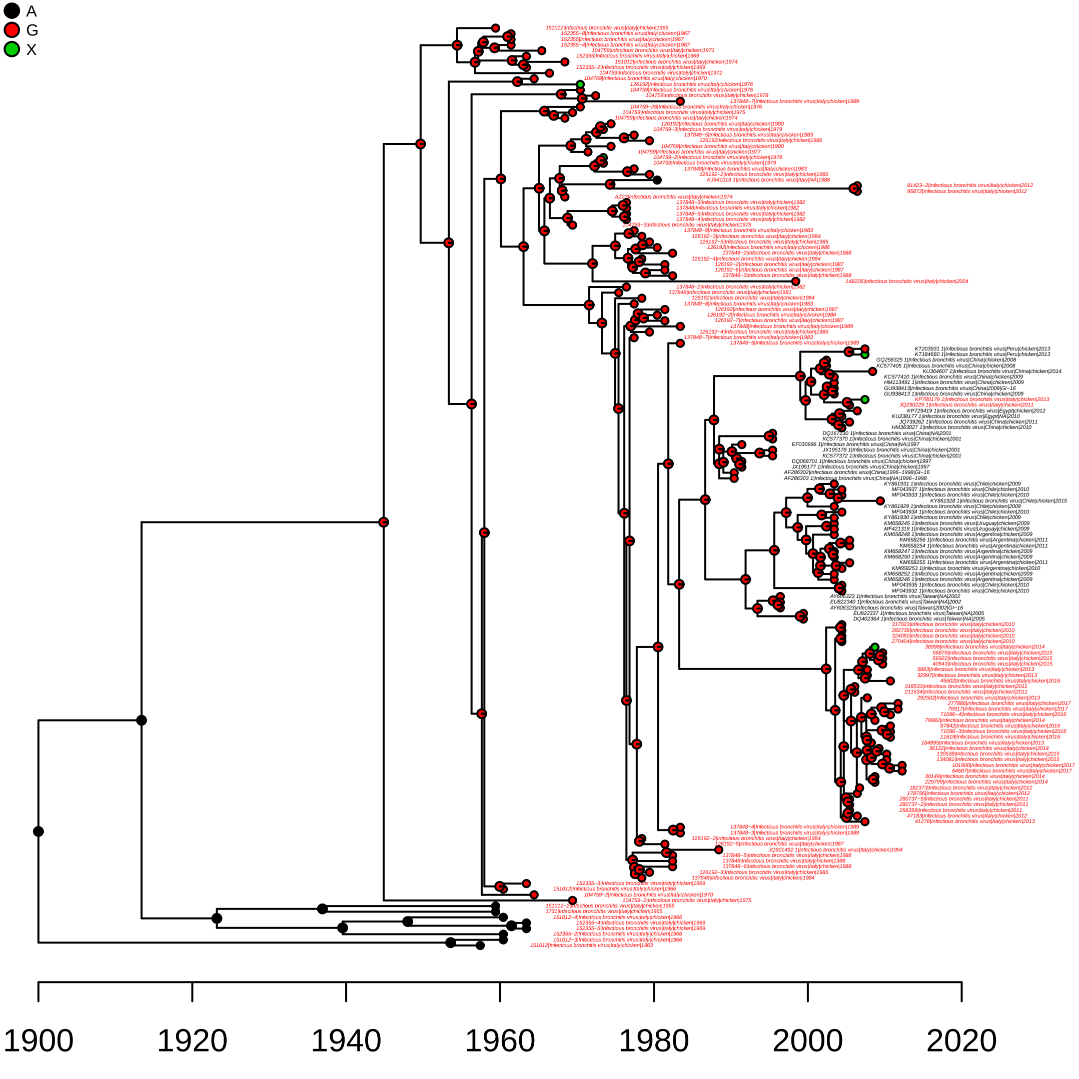

117

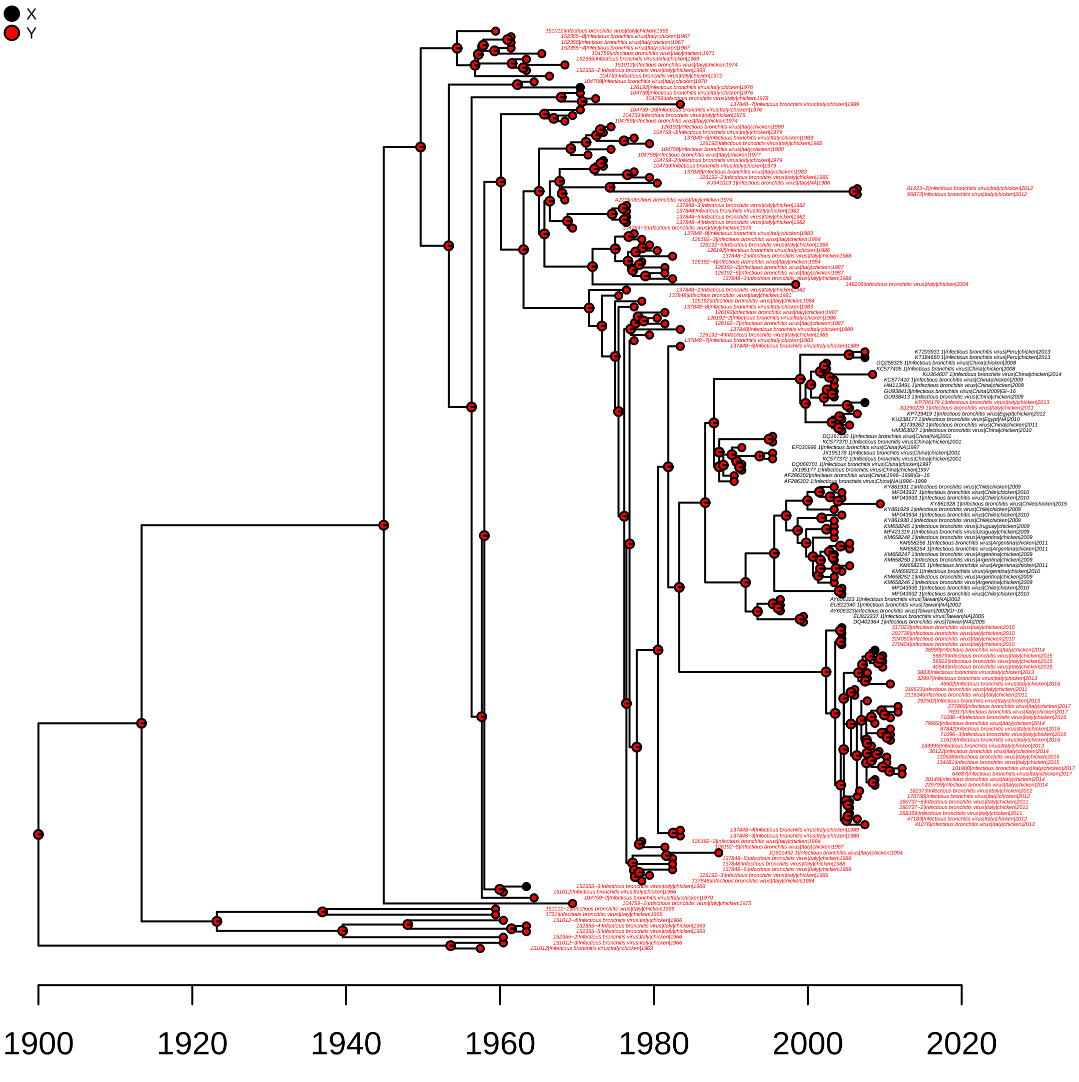

118

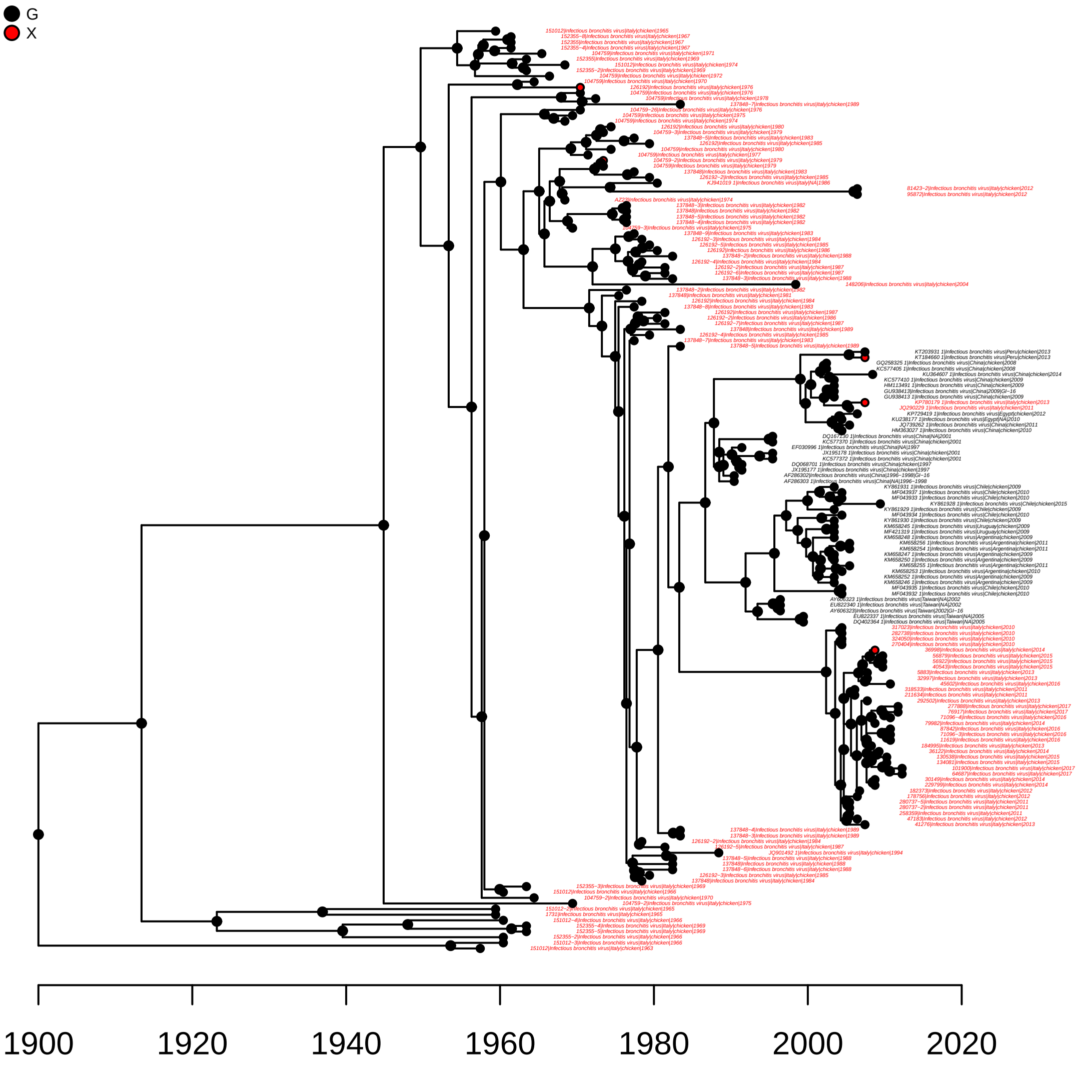

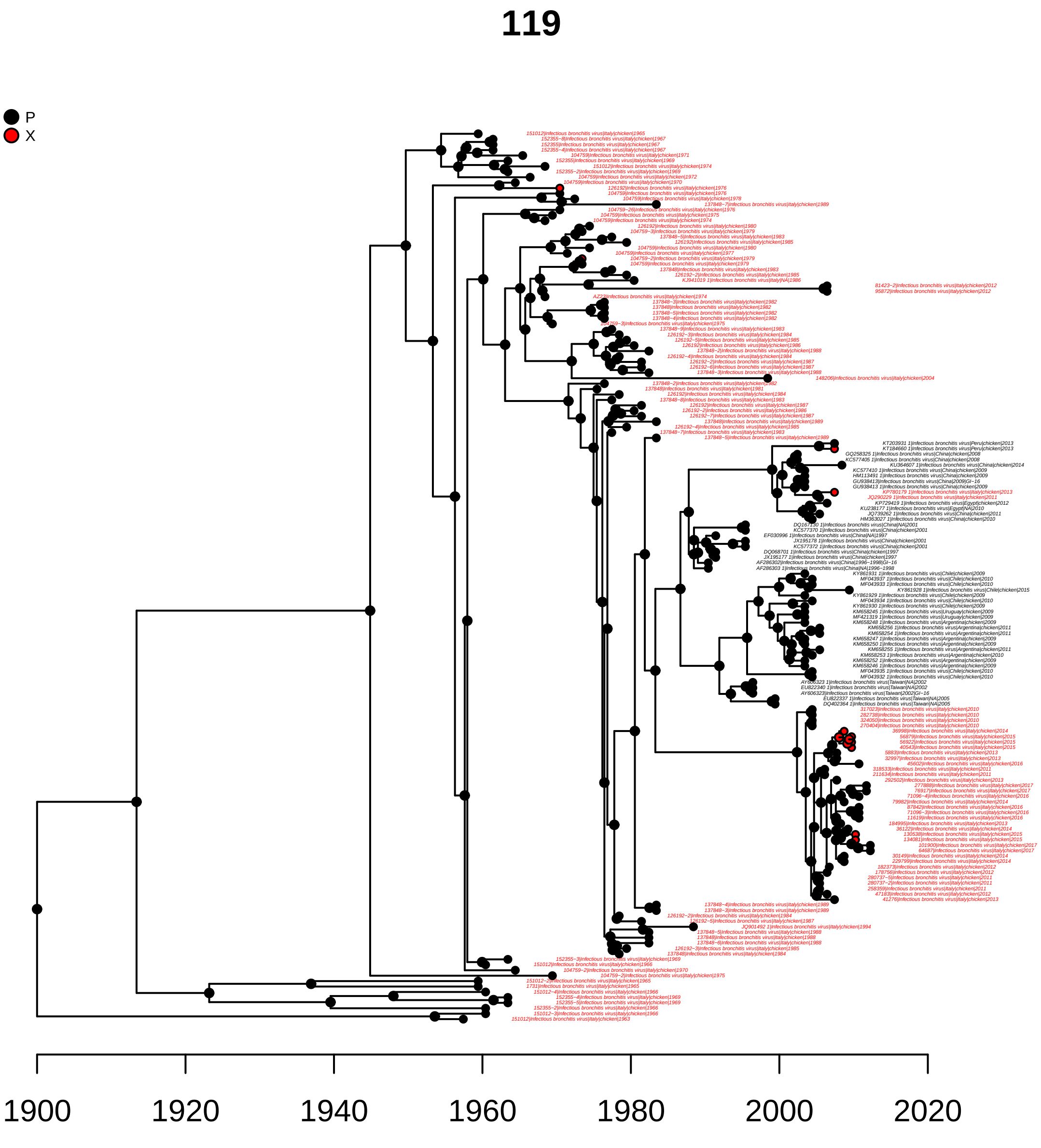

119
P
X
1900
1920
1940
1960
1980
2000
2020

120

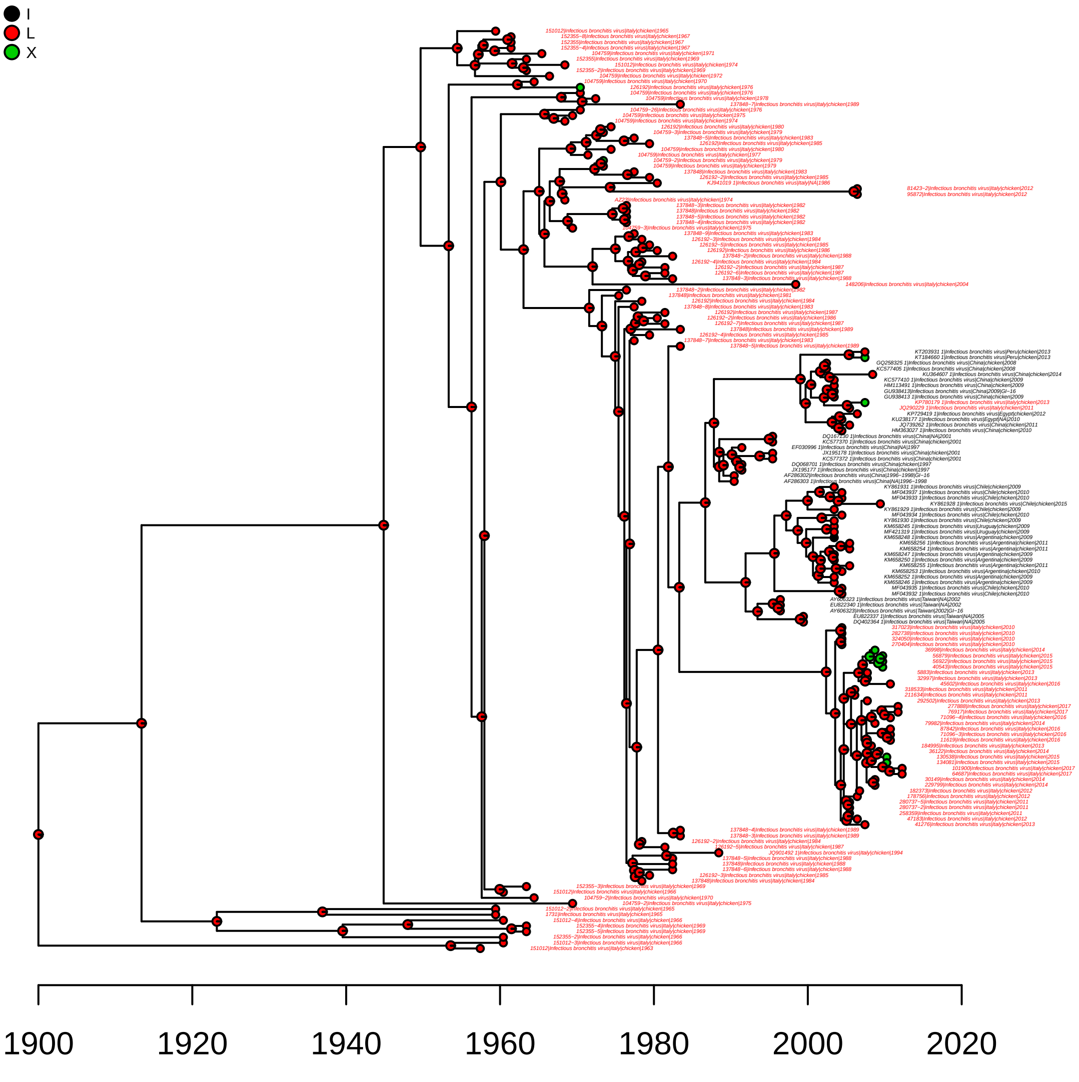

121

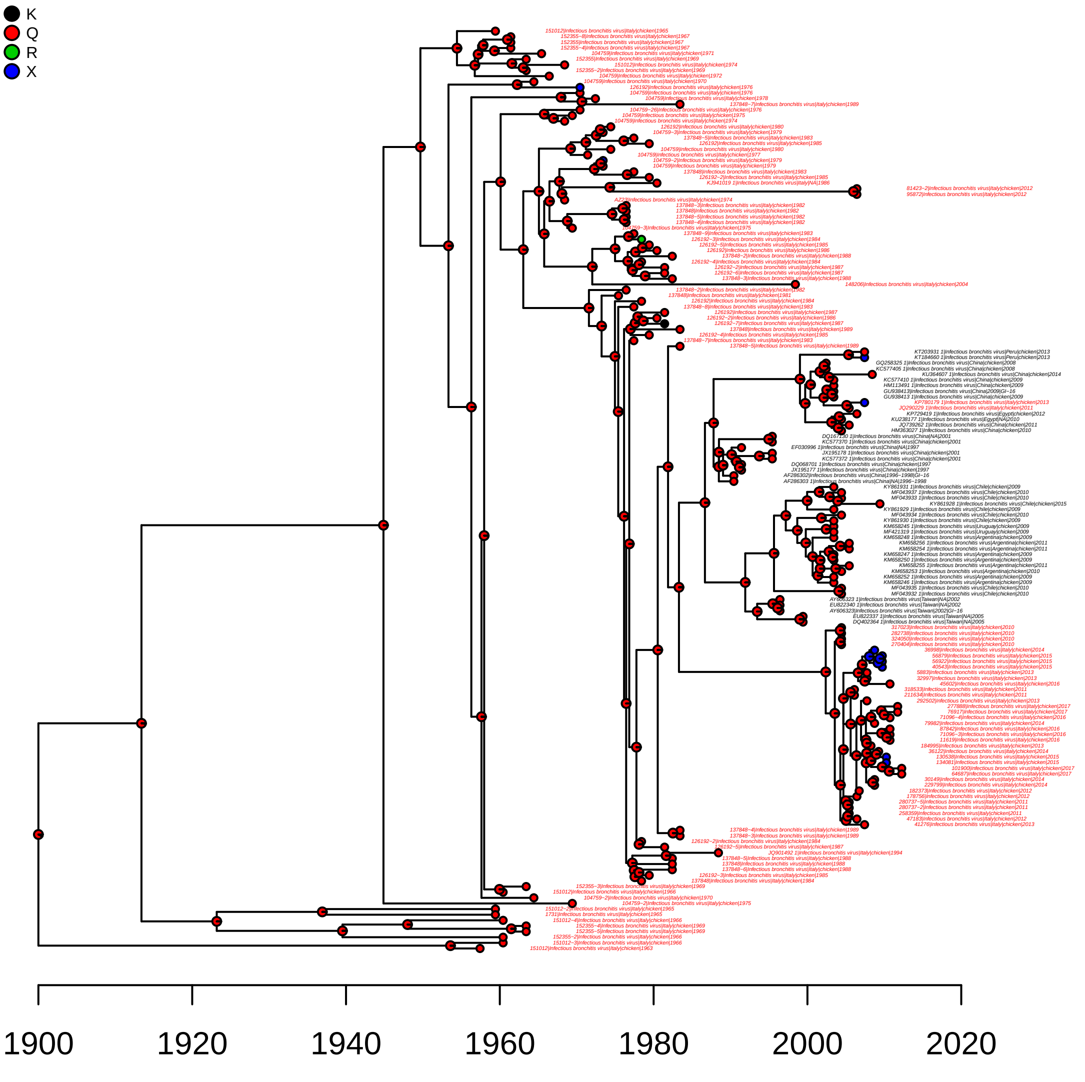

122

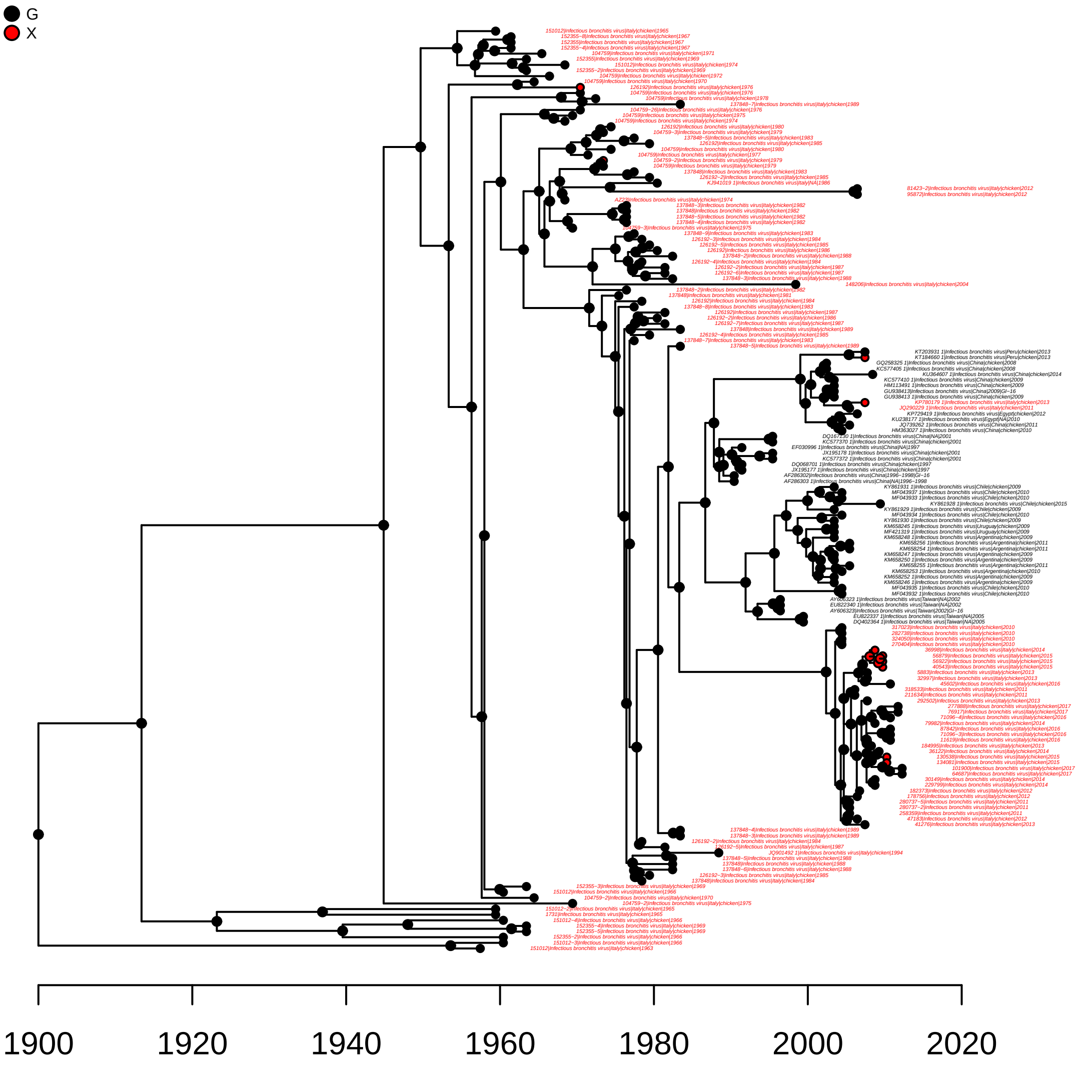

123

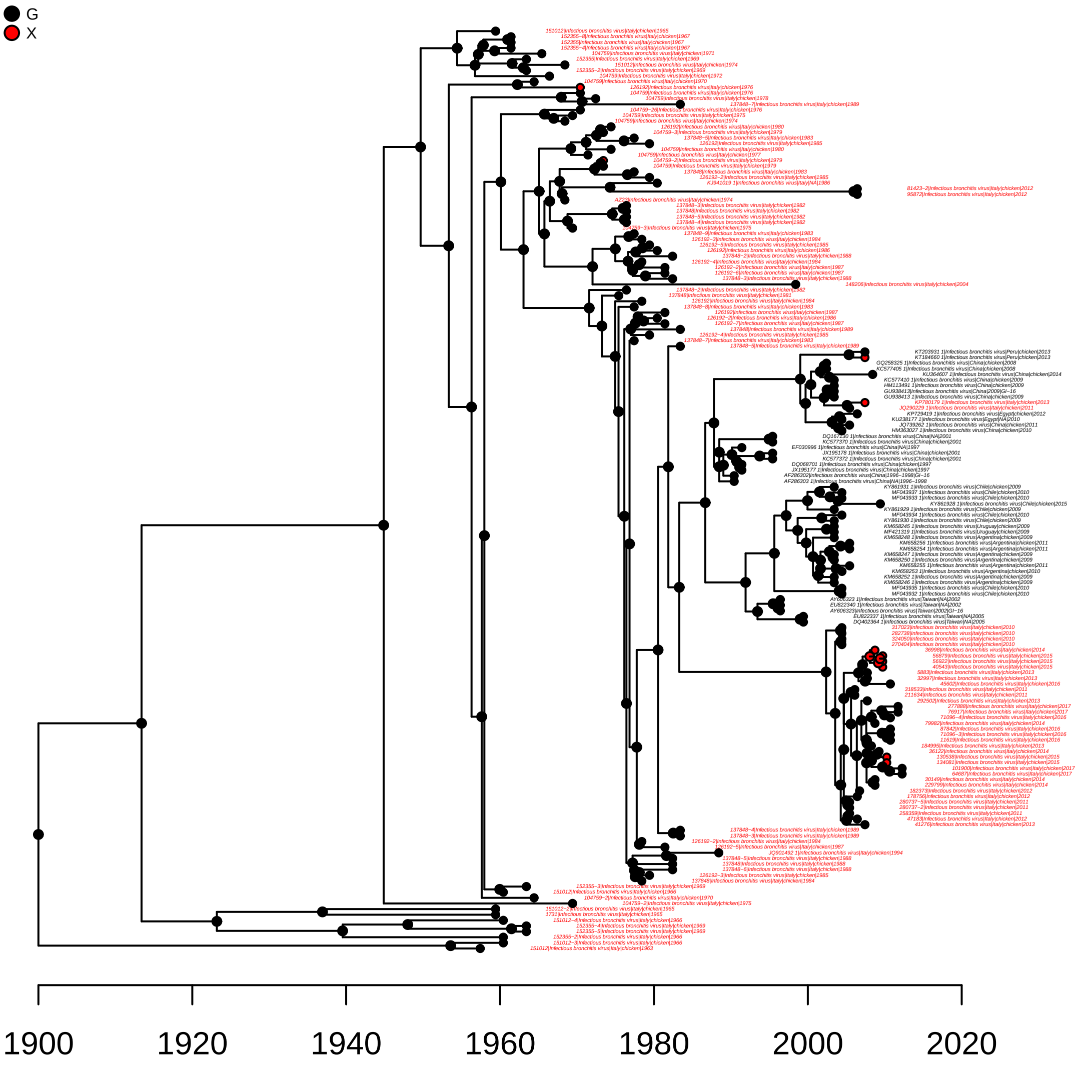

124

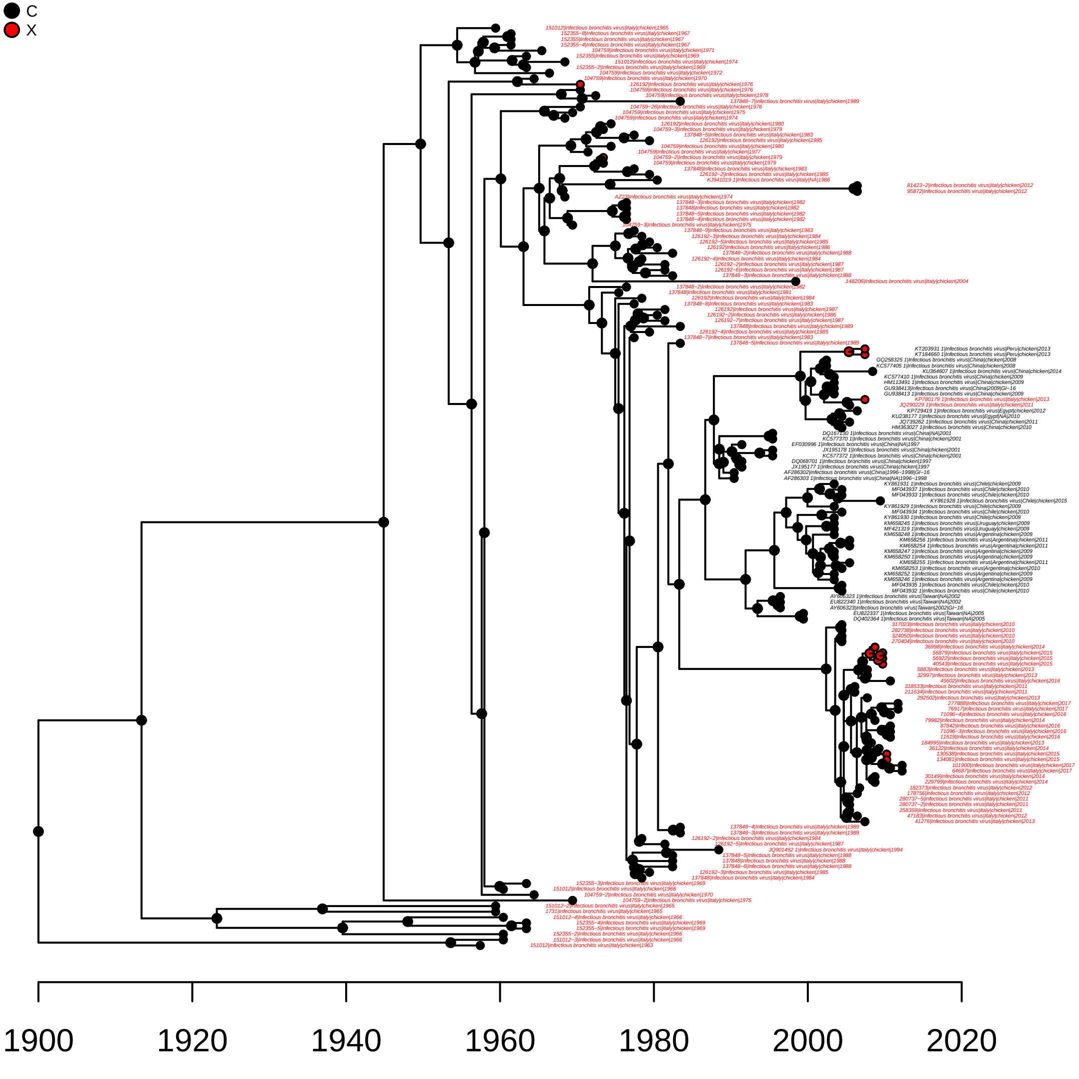

125

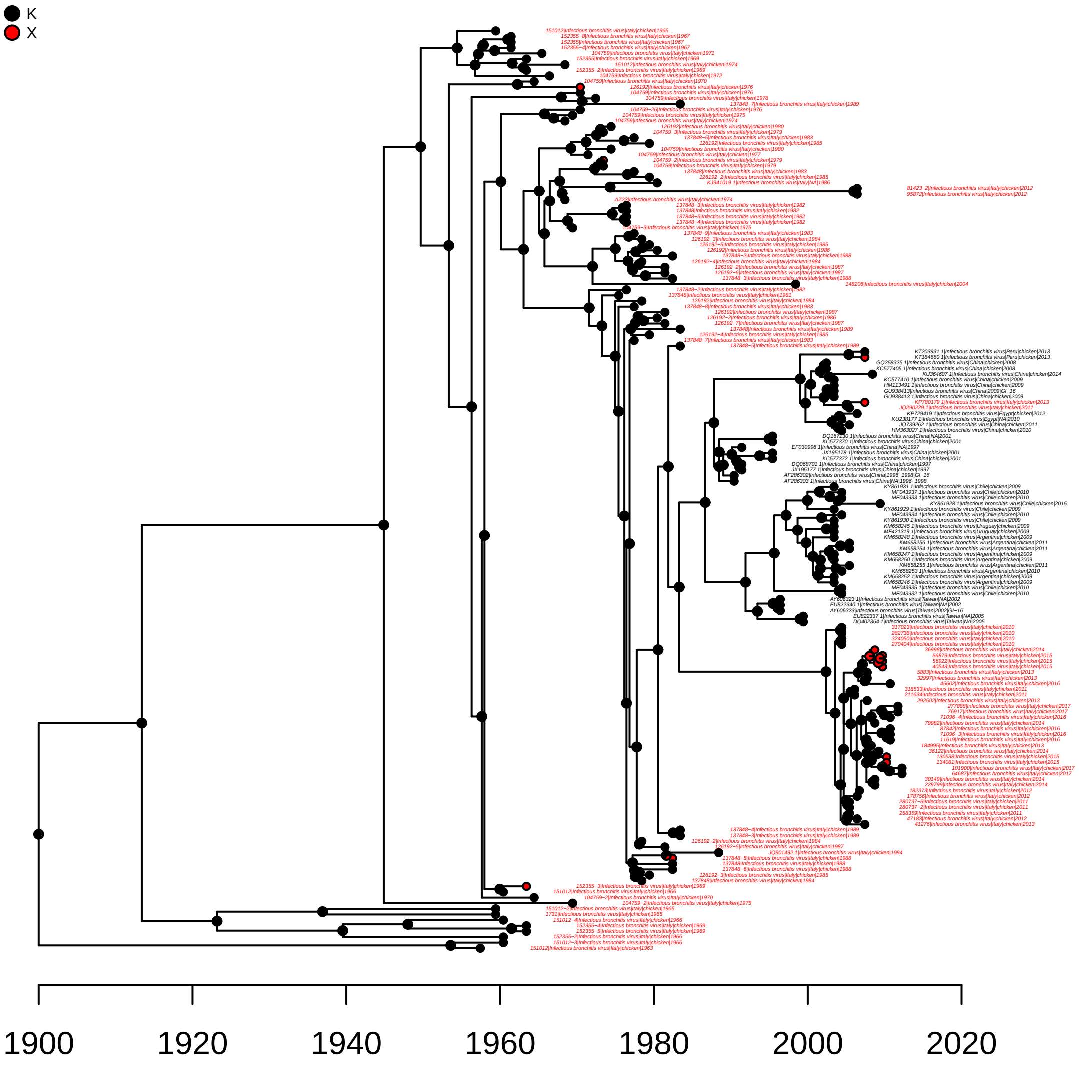

126

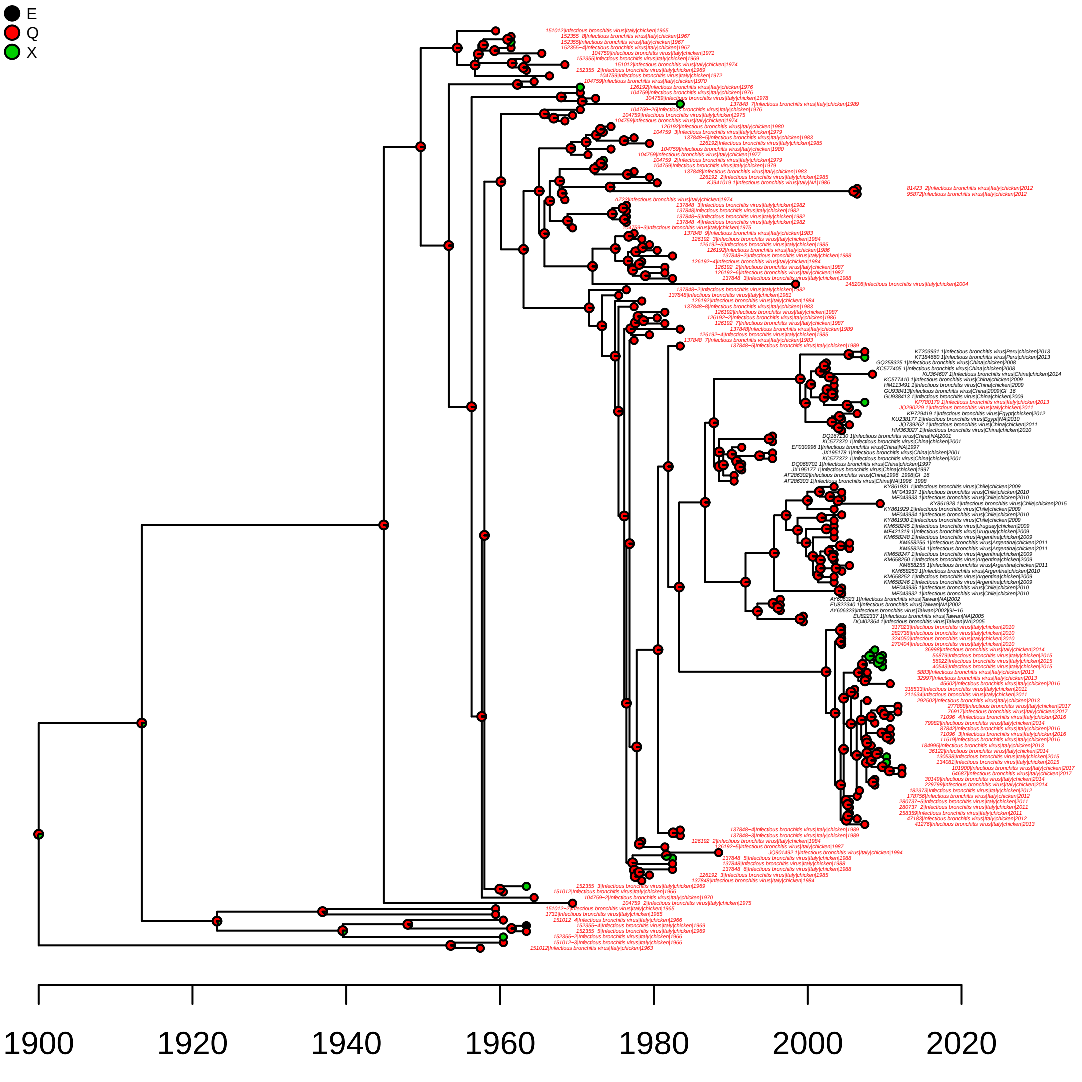

127

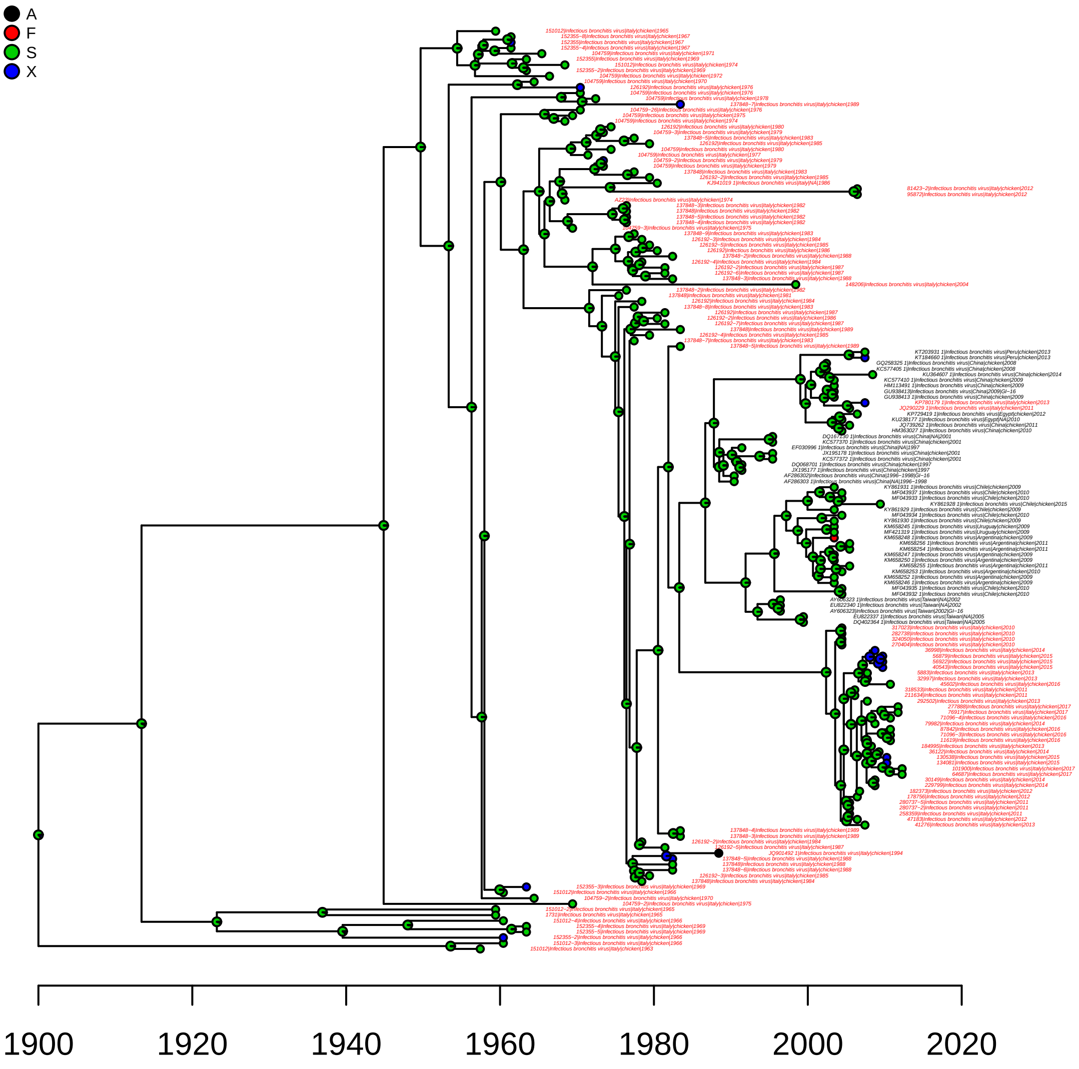

Supplement: S3 Fig — Maximum likelihood based reconstruction of amino acid ancestral state variation over time. Tips (sampled strain) amino-acids are reported as a color coded circle for each HVR3 position. The predicted amino-acids for each internal node (ancestral strains) are reported as a color coded pie-chart with slices proportional to the respective probabilities. (PDF) [file pone.0203513.s003.pdf]
